# Risk assessment of stock market manipulation through the fusion of multi-source textual and trading data: Evidence from China’s A-share market

**DOI:** 10.1371/journal.pone.0343442

**Published:** 2026-08-07

**Authors:** Yuting Luo, Jian Zhang, Changlu Zhang, Zhichao Ma

**Affiliations:** 1 College of Management Science and Engineering, Beijing Information Science and Technology University, Beijing, China; 2 Beijing Key Lab of Green Development Decision Based on Big Data, Beijing, China; Bucharest University of Economic Studies: Academia de Studii Economice din Bucuresti, ROMANIA

## Abstract

Against the backdrop of increasingly diversified and concealed forms of stock market manipulation, approaches based solely on trading data or financial indicators face growing limitations in complex and information-intensive market environments. To assess stock market manipulation risk, this study constructs a firm–month level multi-source panel dataset by retrospectively labeling violation periods at the monthly frequency based on manipulation cases sanctioned by the CSRC (China Securities Regulatory Commission). The dataset integrates corporate disclosures, investor sentiment derived from online public opinion, and market trading characteristics. A supervised learning framework that fuses textual representations and numerical features is then employed to generate manipulation risk probabilities, supporting risk ranking and tiered screening in regulatory applications. Empirical results show that the fusion model consistently outperforms single-source baselines, achieving an AUC of 0.8811 and a PR-AUC of 0.6943, along with substantial improvements in Recall@10% and Recall@20% for high-risk screening. These findings indicate that multi-source information exhibits complementary effects in manipulation risk assessment and enables effective characterization of joint anomalies along the “information disclosure–sentiment reaction–trading behavior” chain. Theoretically, this study highlights the complementary role of heterogeneous information sources, including disclosure, sentiment, and trading-related signals, in characterizing manipulation risk. In practice, it provides a feasible data-driven pathway for risk monitoring and tiered regulatory screening.

## 1. Introduction

Against the backdrop of highly informatized capital markets and increasingly complex trading mechanisms, stock market manipulation has evolved toward more concealed and strategic forms, posing persistent challenges to market fairness and regulatory effectiveness [1]. Recent regulatory evidence indicates that stock market manipulation remains a persistent concern in China’s A-share market. For example, the China Securities Regulatory Commission (CSRC) reported that it investigated 701 violation cases in 2025, including 85 cases of market manipulation, with total fines and confiscations exceeding 15 billion yuan. These findings highlight the continued prevalence and substantial economic impact of manipulation activities. Traditional manipulation typically relies on concentrated trading to directly influence the price formation process. However, with the expansion of information dissemination channels and the changing structure of investors, manipulative activities are increasingly accompanied by abnormal information disclosure, fluctuations in market sentiment, and interconnected price reactions [2,3]. Under the practical constraint of limited regulatory resources, regulators must allocate attention across a large number of market participants and prioritize observations that exhibit elevated risk levels. In this context, approaches based on single-source data provide limited support for reliable risk prioritization, motivating the need for data-driven frameworks based on observable multi-source signals.

Existing research on stock market manipulation primarily focuses on detecting trading anomalies. Early studies relied on market variables such as prices and trading volumes, employing regression analysis, statistical tests, or machine learning techniques to characterize abnormal trading patterns. Nevertheless, such approaches face limitations in capturing increasingly concealed and information-based manipulation patterns, particularly when relying on single-source data [4]. With the improvement of disclosure systems and the rise of social media, recent studies have incorporated textual information and public sentiment into analysis, showing that disclosure tone, investor attention, and online discussions are associated with stock price dynamics and risk exposure [5,6]. Natural language processing–based methods have further extended manipulation analysis into the informational domain [7,8]. Despite these advances, existing approaches typically examine different information sources separately. Differences in temporal granularity, update frequency, and data representation further hinder their integration within a unified analytical framework. As a result, current models remain limited in capturing manipulation risk as a coordinated process integrating disclosure behavior, sentiment diffusion, and market responses, and continue to focus primarily on binary classification rather than risk ranking and tiered screening.

To address these limitations, this study aims to assess stock market manipulation risk by developing a multi-source analytical framework that integrates corporate disclosures, investor sentiment, and market trading behavior. In practice, stock market manipulation involves heterogeneous strategies, including trade-based, information-based, and combined forms. Rather than identifying specific manipulation types, this study focuses on a screening-oriented risk assessment framework that captures common abnormal patterns observable across different manipulation behaviors. Methodologically, the framework combines structured numerical variables with textual features extracted using BERT (Bidirectional Encoder Representations from Transformers), a deep contextual language model, enabling collaborative modeling of heterogeneous features under a unified temporal scale. This approach facilitates the characterization of manipulation risk through the joint dynamics along the “information disclosure–sentiment reaction–trading behavior” chain and produces interpretable risk probabilities. Compared with prior research, this study offers three main contributions. First, it develops a unified multi-source framework that captures manipulation risk through the joint dynamics of information disclosure, sentiment diffusion, and market trading behavior under a consistent temporal structure. Second, it introduces a BERT–Multiple Instance Learning (BERT–MIL) architecture that models multiple announcements within the same period as a set and captures heterogeneous and unevenly distributed information signals that are often diluted in conventional semantic analysis. Third, it formulates stock market manipulation analysis as a probabilistic risk assessment problem that supports risk ranking and tiered screening in regulatory practice, and further enhances interpretability through SHAP (SHapley Additive exPlanations). The findings contribute to a deeper understanding of the observable signal structure associated with stock market manipulation risk and provide empirical support for data-driven risk assessment and regulatory decision-making in the context of regulatory technology.

## 2. Literature review

### 2.1. Risk assessment of stock market manipulation

Research on stock market manipulation has evolved from theoretical explanations of manipulation mechanisms to empirical identification using observable market anomalies. Early studies examined manipulation from the perspectives of market microstructure and information asymmetry, showing how manipulators may profit by exploiting frictions in the price formation process [3,9]. While these studies clarify the economic logic of manipulation, they provide limited guidance for constructing operational indicators for large-scale empirical screening and risk assessment.

With the increasing availability of trading data and regulatory disclosures, empirical research has increasingly operationalized manipulation as a data-driven identification problem. In the Chinese regulatory context, Liu et al. used administrative penalty cases disclosed by the CSRC (China Securities Regulatory Commission) to develop machine learning models and showed that trading intensity, price volatility, and transaction-structure variables have discriminative value for identifying manipulation [5]. Related studies have further examined classification methods, multidimensional abnormal trading indicators, and price-pattern-based approaches for detecting potential manipulation, insider trading, or atypical stock transactions [10–13]. These studies improve the empirical feasibility of manipulation analysis by translating manipulation into measurable features and classification tasks. However, their evidence is still largely organized around trading anomalies and binary detection, providing limited insight into manipulation risk as a probabilistic state shaped by disclosure behavior, sentiment diffusion, and market responses.

Building on this foundation, later studies have paid increasing attention to heterogeneity across manipulation strategies and the evolution of detection frameworks. Allen and Gorton offered an early theoretical distinction among action-based manipulation, trade-based manipulation, and information-based manipulation [3]. Siering et al. further refined this perspective by showing that different manipulation techniques may involve distinct trading paths, information channels, and market responses [14]. Recent reviews and surveys of manipulation detection emphasize multi-feature learning rather than single-rule screening [4,15]. RegTech studies also position automated risk identification as an important direction for financial supervision [16,17]. This methodological evolution reflects a practical challenge: contemporary manipulation may combine trading activity, information release, investor attention, and sentiment amplification. Models built around a single manipulation pattern or a single data source may therefore have limited generalizability across risk scenarios.

Meanwhile, the informational dimension has attracted growing attention in manipulation risk assessment. Prior studies indicate that textual tone, disclosure similarity, and financial textual signals are associated with firm risk and abnormal market behavior [6,7,18]. Recent evidence further links investor attention and public information manipulation to stock price manipulation and abnormal price reactions [19,20]. These studies suggest that manipulation risk may be reflected not only in trading outcomes, but also in information disclosure and diffusion. Yet existing research often treats trading, textual, and sentiment signals separately and remains centered on binary detection. This limitation motivates a unified multi-source framework that integrates disclosure, public sentiment, and market trading signals for probabilistic risk assessment.

### 2.2. Information dissemination and public sentiment diffusion in stock markets

With the diversification of information channels and the rise of social media, market reactions are no longer shaped only by formal disclosures and traditional news reports. The speed, tone, and pathway of information diffusion also affect how investors process signals and adjust expectations. Prior research suggests that investor responses to information shocks are not fully rational; public sentiment diffusion, emotional framing, and social transmission can shape investor decisions and market dynamics [21]. Wang et al. further show that sentiment changes in online forums are related to stock returns, indicating that investor-generated discussions can enter the price formation process [22].

Related studies further examine how sentiment embedded in news and media content affects market outcomes. Gambarelli and Muzzioli examined the relationship between news tone and stock returns, showing that positive news sentiment is associated with short-term price increases, while negative sentiment is associated with greater market volatility [8]. Obaid and Pukthuanthong further show that machine-learning-based sentiment measures extracted from news images contain information about investor sentiment and market pricing [23]. These studies establish the market relevance of sentiment, but they mainly examine sentiment as a reaction to information, rather than as a channel through which risk may be amplified.

Research on social networks further shows that information effects depend on diffusion structure. Teplova et al. reported that both information attention and sentiment dynamics within social networks influence retail investors’ trading decisions, reflecting pronounced attention-driven characteristics [24]. In manipulation contexts, this diffusion structure matters because attention bursts and concentrated discussions may reinforce abnormal price movements. Some studies have further explored the role of information manipulation within sentiment diffusion processes. Tan et al. provided empirical evidence that the spread of false information can distort investor behavior and intensify stock price fluctuations [25]. Evidence on public information manipulation and rumor verification further suggests that distorted information flows can interact with market feedback and investor reactions [26]. Together, these findings suggest that sentiment diffusion is relevant to manipulation risk when it is amplified, distorted, or strategically used.

Overall, existing research shows that information and public sentiment are important for understanding market reactions and manipulation risk. Methodologically, this literature has moved from single-source sentiment analysis toward broader perspectives that consider network effects, emotional polarization, and information dissemination. Nevertheless, prior studies remain largely focused on single-channel informational analyses and lack integrated quantitative frameworks that jointly capture sentiment diffusion and market responses, particularly in the context of manipulation risk assessment within complex financial markets.

### 2.3. Applications of multi-source data fusion in financial regulation

As financial systems become more complex and risk patterns evolve, financial regulation increasingly addresses interconnected issues such as market manipulation, financial fraud, disclosure violations, investor behavior, and systemic risk transmission. In this context, prior studies suggest that reliance on a single data source, such as trading records or financial statements, provides limited support for forward-looking and interpretable risk assessment. Multi-source data fusion has therefore become an important direction in financial regulatory technology (RegTech) [4,19].

The first stream of research expands risk assessment variable systems from single-source indicators to multi-source representations. Early financial risk assessments primarily relied on financial and trading indicators, employing regression models or structural equation modeling to capture abnormal trading activities and price distortions [27,28]. As regulatory problems become more information-intensive, transaction-level or financial indicators alone are less able to capture concealed and information-based risks. Yuan et al. showed that integrating financial indicators with unstructured information improves the stability and interpretability of financial fraud risk identification [29]. Using CSRC enforcement samples, Liu et al. further demonstrated that multidimensional feature systems outperform single trading features in manipulation detection [5]. These studies justify feature expansion, but they do not fully address how heterogeneous signals should be aligned and fused for risk ranking rather than simple classification.

The second stream of research focuses on the complementary value of textual data and the broader informational environment in regulatory contexts. Prior studies suggest that annual report texts, disclosure tone, and reporting patterns can reveal firms’ underlying risk behaviors. Through the classification of SEC (U.S. Securities and Exchange Commission) comment-letter texts, Ryans showed that regulatory documents themselves contain important risk signals [30]. Chen et al. incorporated semantic features derived from annual reports into machine learning models and improved the identification of financial distress and related risks [31]. Examining the consistency between public sentiment and textual tone, Yen et al. found that public sentiment and semantic textual features can serve as important leading indicators of financial risk [32]. These studies demonstrate that textual and information environment data capture risk signals that are not directly observable from transaction outcomes alone. However, most of this evidence is developed in financial distress, fraud, or general risk settings, and the interaction between textual signals and market-trading responses remains less developed in manipulation risk assessment.

The third stream moves toward the joint modeling of textual information, public sentiment, and market behavior for regulatory decision-making. Yao et al. demonstrated the advantages of multi-source fusion across the data, model, and decision layers, showing that integrating textual and trading data can reduce the noise of single-source signals [33]. Cao et al. proposed a human–machine collaborative analytical paradigm, emphasizing that model outputs should complement regulatory experience and expert judgment to enhance practical usability [34]. Wang and Zheng further highlighted that multi-source analysis helps characterize the links among information manipulation, institutional changes, and market responses [35]. Yet many fusion studies emphasize performance gains or decision support in broad financial-risk settings, while the role of fusion in probabilistic manipulation risk assessment and screening remains insufficiently specified.

Taken together, existing research supports the value of multi-source information in financial regulation, but important gaps remain in how heterogeneous disclosure, sentiment, and trading signals are jointly represented for manipulation ranking. Building on this literature, the present study integrates announcement texts, public sentiment, and market trading information within a unified risk assessment framework to evaluate their complementary value in probabilistic risk scoring and tiered screening.

## 3. Multi-source risk representation and variable construction

### 3.1. Conceptual basis of multi-source risk representation

In modern stock markets, price formation reflects the interaction between formal corporate disclosures, online public sentiment, and market trading responses. Information diffusion can influence investors’ beliefs and expectations, thereby affecting price adjustment, volatility, and trading activity [36]. Recent evidence further suggests that manipulation risk may increase in opaque or uncertain information environments, where investors face greater difficulty distinguishing fundamental price movements from strategically induced market signals [37]. This implies that manipulation-related risk should not be assessed solely through isolated trading outcomes, because abnormal market behavior may be accompanied by changes in disclosure patterns and information diffusion. A multi-source information perspective therefore provides the conceptual basis for representing manipulation-related risk through observable public signals and guides the construction of the firm–month risk variables used in the empirical model.

In practice, stock market manipulation may arise through heterogeneous channels. Some strategies are mainly trade-based and are reflected in abnormal price or volume movements, whereas others are more information-based and involve misleading disclosure, rumor diffusion, or sentiment amplification. In many cases, trading and information channels may also interact, forming combined manipulation patterns. These strategies differ in their underlying mechanisms and observable manifestations. Given such heterogeneity, this study does not seek to identify or classify specific manipulation strategies, but instead adopts a screening-oriented risk assessment perspective. The objective is to assess whether common observable risk patterns across heterogeneous manipulation forms can be represented through the joint dynamics of disclosure behavior, sentiment diffusion, and market responses. Accordingly, the model output should be interpreted as a firm–month manipulation-risk score for candidate review, rather than as transaction-level evidence of specific manipulative actions or actors.

Within this conceptual framework, manipulation-related risk can be represented through the complementary roles of three observable information dimensions. First, formal disclosures issued by listed firms reflect how firms release, frame, and communicate information to the market. Disclosure frequency, tone, textual complexity, and reporting patterns may influence investor interpretation and provide observable signals of the firm’s information environment [6,30,38]. Second, public sentiment generated by market participants captures investor attention, discussion intensity, and the diffusion of expectations in online information environments. Prior studies suggest that investor sentiment, rumors, and platform-based interactions are associated with market reactions and abnormal information diffusion [21,22,25]. Third, market responses capture the market-level manifestation of risk signals through returns, volatility, trading intensity, and market value changes. Evidence on public information manipulation and trading-based manipulation further indicates that such risks may be reflected in asset prices through market feedback and abnormal trading responses [20,39]. These three dimensions correspond to the main feature groups used in this study: announcement-text features, public sentiment features, and market trading features. They are not intended to support isolated variable-level hypotheses; rather, they jointly define the observable signal space for multi-source risk representation. From a dynamic perspective, manipulation risk may be reflected when unusual disclosure patterns, concentrated sentiment diffusion, and abnormal market responses co-occur within the same firm–month risk window. The following section operationalizes this conceptual framework by constructing variables that capture each of these three dimensions.

### 3.2. Construction of risk representation variables from a multi-source data perspective

#### 3.2.1. Announcement text features.

Corporate announcements are among the most formal and authoritative sources of information in financial markets. Their content directly influences investors’ assessments of firms’ operating conditions, risk levels, and future prospects, thereby playing an important leading role in the formation of manipulation risk [38]. Building on prior research, this study examines announcement characteristics from three dimensions.

First, disclosure intensity is measured by the monthly number of announcements (ann_cnt), capturing the frequency of corporate information releases during a given period. Under normal conditions, the volume of announcements tends to vary in a relatively stable manner in line with firms’ operational rhythms. An unusual surge in announcement frequency within a short period, however, may indicate attempts to shape market expectations, sustain investor attention, or mitigate the impact of unfavorable information. Disclosure intensity may therefore serve as an early signal of manipulation risk.

Second, sentiment orientation is assessed by calculating the sentiment score of announcement texts (ann_sent) to evaluate the emotional tone of corporate disclosures. Methodologically, sentiment words are identified using a Chinese financial sentiment lexicon constructed with reference to prior Chinese financial sentiment dictionaries [40]. For each announcement i , the occurrences of positive and negative words are counted and denoted as ${Pos}_{i}$ and ${Neg}_{i}\;$, respectively. Based on these counts, the announcement-level sentiment score is defined as follows:


$$\begin{array}{c} {Sent}_{i}=\frac{{Pos}_{i}-{Neg}_{i}}{{Pos}_{i}+{Neg}_{i}+\text{1}} \end{array}$$ (1)

A higher value of this indicator suggests a more positive overall tone in corporate announcements, whereas a lower value indicates the dominance of negative sentiment. Announcement tone reflects how firms convey operating conditions and risk information to investors. When sentiment persistently deviates from underlying fundamentals or exhibits unusually optimistic expressions at critical time points, it may be associated with attempts to shape market expectations and can therefore serve as a potential risk signal. In addition, financial sentiment measurement is sensitive to language and domain context, which supports the use of Chinese financial sentiment resources when analyzing Chinese financial texts [41]. In this study, the sentiment score is used as a transparent and reproducible proxy for announcement tone. Given the inherent limits of word count measures in representing contextual meaning, the sensitivity of this measurement choice is further examined in Section 4.3 using alternative sentiment measures constructed with a transformer model.

Third, announcement textual complexity is measured using a Chinese-adapted readability index (ann_fog). Readability is an important dimension of disclosure quality because complex textual expression may increase investors’ information-processing costs and reduce disclosure transparency [42]. Evidence from the Chinese market also suggests that the readability of financial disclosures contains useful information for assessing firm risk, including subsequent stock price crash risk [43]. In this study, ann_fog is used as an observable proxy for the linguistic complexity of corporate announcements within the multi-source risk representation framework.

The original Gunning Fog Index was developed for English texts and identifies complex words based on syllable structure, which is not directly applicable to Chinese. Drawing on Chinese financial-text readability studies, particularly Li et al. [44], this study constructs the index based on average sentence length and the complex-word ratio:


$$\begin{array}{c} {ann\_fog}_{i}={ASL}_{i}+{CWR}_{i} \end{array}$$ (2)

where ${ASL}_{i}\;$denotes the average sentence length, calculated as the total number of segmented words divided by the number of sentences, and ${CWR}_{i}$ represents the complex-word ratio, calculated as the proportion of segmented words containing three or more Chinese characters. A higher value of ann_fog indicates greater textual complexity and lower readability.

#### 3.2.2. Public sentiment features.

In highly networked financial markets, public sentiment has become an important source of information influencing stock price formation and volatility. Compared with formally disclosed corporate information, sentiment data more directly capture the market’s immediate reactions and the evolution of expectations. Prior studies show that investor attention and online discussion dynamics are closely related to market reactions and manipulation risk [19,21,26]. Accordingly, this study examines investors’ attention to individual stocks and the structural characteristics of market sentiment from three dimensions.

First, sentiment activity and participation breadth are measured using the monthly number of posts (post_cnt) and the number of participating users (user_cnt), reflecting the intensity of market attention and the breadth of investor participation. The monthly post count captures the overall frequency of investor discussions surrounding a given stock, while the number of users represents the scale of distinct participants involved in these discussions. Together, these indicators summarize the fundamental structure of public sentiment from both volume and participant perspectives, providing baseline information for detecting abnormal sentiment patterns.

Second, sentiment diffusion intensity and interaction depth are assessed by calculating the average number of views (click_mean) and comments (comment_mean) per post within each month. The extent to which sentiment affects the market is closely related to both the reach of information and the level of investor engagement. Average views reflect the exposure of individual posts, whereas average comments indicate the degree of investor interaction around the information. Elevated view counts typically suggest that information has reached a broad investor audience within a short period, while unusual increases in comment volume often coincide with intensified opinion divergence or expectation-driven speculation.

Third, sentiment polarity and temporal distribution are captured through the mean sentiment score (sent_mean) and the standard deviation of daily post counts (post_cnt_std_day), enabling the detection of extreme sentiment and irregular communication rhythms. Sentiment scores are computed using the same approach applied to announcement texts, ensuring measurement consistency across information sources and reflecting the overall optimism or pessimism of market sentiment. The standard deviation of daily posts measures the intra-month dispersion of sentiment activity. When discussions surge within only a few trading days, the standard deviation rises markedly, often corresponding to concentrated information releases, rapid sentiment aggregation, or unusual trading behavior. As such, this variable serves as an important complementary indicator for capturing abnormal sentiment patterns associated with manipulation risk.

#### 3.2.3. Market trading features.

Trading behavior reflects how information and investor expectations are incorporated into price movements, liquidity conditions, and capital flows. Prior studies document that market manipulation is often associated with abnormal price effects and shifts in trading activity [5,39]. In this study, trading indicators constitute the market behavior dimension of the multi-source risk representation and complement announcement and sentiment features within the “information disclosure–sentiment reaction–trading behavior” chain. Guided by this rationale, this study constructs market trading indicators from the following aspects.

First, return and price response characteristics. Stock returns (Mretwd) and cumulative abnormal returns (CAR) are used to measure realized price dynamics and abnormal deviations from broader market performance. CAR is computed as the within-stock cumulative sum of monthly abnormal returns, where monthly abnormal return equals Mretwd minus the market return weighted by circulating market capitalization in the same month. Prior studies on market manipulation document abnormal return patterns and price reversals in manipulation cases [3,5,39]. In this context, sustained excess returns may reflect the incorporation of information signals, sentiment amplification, or concentrated trading pressure into prices. Accordingly, Mretwd and CAR are included to characterize the return-based price response dimension of manipulation risk within the market trading feature group.

Second, trading activity. Turnover rate (TR) is employed to capture the frequency of stock transactions, while the ratio of trading value to circulating market capitalization (Tra/Cir_Market_Cap) measures the intensity of capital involvement, thereby improving comparability across firms of different sizes. Trading activity reflects investors’ willingness to transact and the overall strength of market participation. Elevated turnover typically signals active trading conditions and may reflect short-horizon speculative pressure or concentrated capital participation in abnormal market contexts. The ratio of trading value to circulating market capitalization helps mitigate firm-size effects and more accurately captures capital attention toward individual stocks. A pronounced increase in this indicator may reflect concentrated capital inflows over a short period, thereby serving as a potential risk signal.

Third, price volatility and stability characteristics. Volatility (Vol) is measured using the rolling standard deviation of monthly returns, while the market capitalization change rate (MCCR) is calculated as the percentage change in total market capitalization relative to the previous month. Price volatility represents a concentrated manifestation of market uncertainty; under conditions of information manipulation or sentiment-driven trading, prices may display sharp or irregular fluctuations. In this study, volatility is computed as the rolling standard deviation of Mretwd over the most recent six observed months, including the current month, with at least three valid monthly observations required. The six-month window is used as a medium-horizon volatility proxy at the monthly frequency, balancing the need to smooth transitory fluctuations while retaining recent abnormal price instability relevant to manipulation risk. Changes in market capitalization reflect the market’s reassessment of firm value. In abnormal market contexts, rapid price increases associated with sentiment diffusion or capital inflows may coincide with abnormal expansions in market capitalization, whereas subsequent reassessment may lead to sharp adjustments. Indicators of volatility and stability therefore help characterize the evolution of market risk from the perspective of price adjustment and market stability.

### 3.3. Summary of the indicator system

In summary, this study consolidates announcement-text features, investor sentiment features, and market trading features into Table 1. These three groups of indicators correspond to the three stages of the “information disclosure–sentiment reaction–trading behavior” chain and constitute the core inputs to the supervised learning model. Overall, we prioritize variables that are frequently used in prior studies, have clear financial interpretations, and remain explainable in regulatory risk assessment settings. All features are organized at a unified temporal granularity to form a multi-source feature matrix that supports comparison and fusion across data sources.

**Table 1:** Multi-source indicator system and variable definitions.

Data dimension	Variable	Indicator name	Definition/ interpretation
**Announcement text**	ann_cnt	Number of announcements	Disclosure cadence and intensity of information release
	ann_sent	Announcement sentiment score	Sentiment orientation of managerial disclosure tone
	ann_fog	Chinese-adapted textual complexity index	Textual complexity measured by average sentence length and complex-word ratio
**Public sentiment**	post_cnt	Number of posts	Investor attention and discussion intensity
	user_cnt	Number of participating users	Breadth of user participation in discussions
	click_mean	Average views per post	Degree of information exposure
	comment_mean	Average comments per post	Depth of discussion and interaction of viewpoints
	sent_mean	Mean sentiment score	Direction of market sentiment
	post_cnt_std_day	Standard deviation of daily post counts	Intra-month rhythm/dispersion of sentiment activity
**Market trading**	Mretwd	Monthly stock return	Monthly individual stock return with cash dividends reinvested
	CAR	Cumulative abnormal return	Extent of persistent deviation from overall market performance
	TR	Turnover rate	Trading frequency/intensity
	Tra/Cir_Market_Cap	Trading value/ circulating market capitalization	Intensity of capital involvement
	Vol	Volatility	Rolling standard deviation of monthly returns
	MCCR	Market capitalization change rate	Value revaluation and phase-wise run-up/pullback trajectory

## 4. Model design and empirical research

### 4.1. Model design

**Fig 1:**
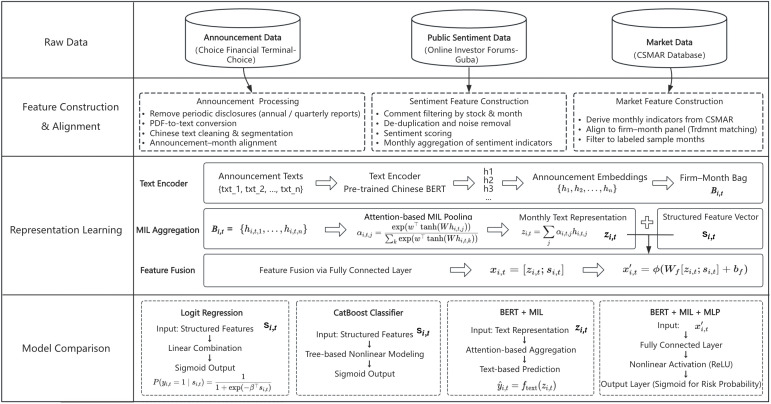
Overall modeling framework.

To systematically evaluate the applicability and effectiveness of different modeling paradigms for stock market manipulation risk assessment, this study introduces four types of models in a unified data processing and feature construction framework, progressing from simpler to more advanced approaches. The models are compared layer by layer in terms of their use of structured features, textual semantic information, and feature fusion strategies. The overall modeling framework is illustrated in Fig 1.

The model design follows a stepwise rationale of “interpretability → nonlinearity → semantic modeling → multi-source fusion.” First, a Logit regression (logistic regression, Logit) using structured features is adopted as the baseline. Second, keeping the inputs unchanged, we replace the baseline with a CatBoost classifier (Categorical Boosting, CatBoost) to examine whether nonlinearities and feature interactions among structured variables improve risk assessment performance. Third, we shift to the text modality by introducing Bidirectional Encoder Representations from Transformers (BERT) representations and combining them with Multiple Instance Learning (MIL) aggregation to assess whether announcement semantics alone can provide effective predictive power. In the final fusion step, the BERT–MIL branch aggregates announcement embeddings into a firm–month textual representation, while the structured-feature branch processes structured announcement indicators, public sentiment variables, and market trading variables in parallel. The two representations are then concatenated and passed to a Multi-Layer Perceptron (MLP) prediction layer, yielding the BERT + MIL + MLP fusion model. Through this progressive design, the study enables a systematic comparison of the incremental contributions of different information sources and modeling strategies, thereby establishing a coherent empirical pathway.

During training and testing, the main model comparison is based on stratified random splits, with 80% of the sample used for training and 20% for testing. To reduce the influence of a particular random partition, all random split experiments are repeated using five random seeds, and model performance is reported as mean ± standard deviation. The same set of random partitions is used across models to ensure comparability. Because the dataset is organized at the firm–month level, Section 4.3.3 further reports a chronological validation to assess temporal generalizability under the panel time structure. Across all models, the output is interpreted as a probabilistic risk score reflecting manipulation-related risk. This interpretation is consistent with the screening-oriented objective of the study, where the model is used to prioritize high-risk observations. Given the imbalanced and screening-oriented nature of the matched firm–month sample, model performance is assessed from two perspectives: overall discriminative ability and high-risk coverage. We use the Area Under the Receiver Operating Characteristic Curve (AUC) and the Area Under the Precision–Recall Curve (PR-AUC) to measure discrimination between risky and non-risky samples across the full dataset. In addition, Recall at Top 10% (Recall@10%) and Recall at Top 20% (Recall@20%) are introduced to evaluate how effectively the model prioritizes high-risk entities under constrained regulatory resources. Specifically, AUC captures a model’s overall ability to separate positive and negative samples across different thresholds, reflecting its global discriminative performance. PR-AUC places greater emphasis on predictive quality for the minority high-risk class, reducing evaluation bias under class imbalance. Recall@K evaluates performance from a practical screening perspective by measuring the proportion of truly sanctioned firms captured within the top K% of cases ranked by the risk score assigned by the model, thereby providing a more direct indicator of practical utility when regulatory resources are limited.

In summary, this study not only compares model performance in a statistical sense, but also emphasizes the practical effectiveness of these models in risk assessment and regulatory screening, providing a unified and interpretable basis for subsequent model comparison and empirical analysis.

#### 4.1.1. Logit regression and catboost machine learning.

This study adopts logistic regression as the baseline model to examine the performance of traditional linear methods in assessing stock market manipulation risk. Owing to its transparent structure and strong parameter interpretability, logistic regression has been widely applied in financial risk research and allows for an intuitive understanding of both the direction and relative magnitude of how risk features influence the probability of manipulation. The primary role of the Logit model in this study is not to achieve optimal predictive accuracy, but rather to provide a benchmark against which the incremental value of nonlinear modeling and textual semantic information can be evaluated.

Building on the baseline specification, we introduce the CatBoost classifier to assess the explanatory power of structured features under nonlinear relationships in stock market manipulation risk. CatBoost is an ensemble learning algorithm based on Gradient Boosting Decision Trees (GBDT) that iteratively combines multiple weak learners to automatically capture nonlinear dependencies and high-order feature interactions [45]. In real-world financial markets, trading behavior, liquidity conditions, and price dynamics often exhibit complex nonlinear patterns. CatBoost demonstrates clear advantages in handling high-dimensional features, modeling variable interactions, and learning nonlinear mappings, making it well suited to the characteristics of financial data.

Both models take the firm–month structured feature vector $S_{i,t}$ as input and output a manipulation risk probability ${\hat{y}}_{i,t}\in [\text{0},\text{1}]$, where i denotes the firm, t denotes the month, and $y_{i,t}\in \{\text{0},\text{1}\}$ denotes the corresponding binary risk label. Specifically, $y_{i,t}\;=\;\text{1}\;$indicates that firm i falls within a manipulation violation interval in month t. Announcement indicators, sentiment features, and market trading variables are jointly incorporated into the regression framework. Without introducing textual representations at this stage, the models focus on structured-data-based risk estimation, allowing us to evaluate the extent to which manipulation risk can be assessed using structured features alone. In the implementation, missing numerical values are imputed using training-set medians. Logit is estimated with standardized inputs, L2 regularization, and balanced class weights, while CatBoost uses a fixed configuration with 500 iterations, depth = 4, learning rate = 0.03, Logloss, AUC, and a training-split scale_pos_weight.

#### 4.1.2. Text modeling based on BERT and MIL.

In information-based market manipulation scenarios, manipulative behaviors are often indirectly transmitted through announcement wording, disclosure strategies, and semantic expression. To capture these signals, this study incorporates textual semantic information to examine the independent contribution of announcement texts to manipulation risk assessment. This design enables a direct evaluation of the explanatory power of textual information and provides a benchmark for the subsequent multi-source fusion model.

To this end, we employ the pre-trained language model BERT to generate vector representations of corporate announcement texts. BERT models contextual semantics through a bidirectional attention mechanism, allowing it to effectively capture nuanced semantic variations in financial language. Compared with traditional approaches based on term frequency or sentiment lexicons, BERT demonstrates clear advantages in semantic representation capability [46]. In the empirical implementation, we use Chinese RoBERTa-wwm-ext, a general-purpose Chinese pretrained model with whole-word masking, and extract the [CLS] embedding from each cleaned announcement text. Given the limited size of sanction-based labeled samples, the BERT encoder is frozen, while the MIL and fusion layers learn task-specific risk representations.

At the firm level, multiple announcements are typically released within a single month. To account for this structure, we adopt a MIL framework to model the set of announcements. Specifically, all announcements issued by firm i in month t are represented as: ${\text{B}}_{\text{i},\text{t}}=\left\{{\text{h}}_{\text{i},\text{t},\text{1}},{\text{h}}_{\text{i},\text{t},\text{2}},\ldots ,{\text{h}}_{\text{i},\text{t},\text{n}}\right\}$; where ${\text{h}}_{\text{i},\text{t},\text{j}}$ denotes the semantic vector of the j-th announcement encoded by BERT.

Because announcements differ substantially in their relevance to risk formation, we implement an attention-based MIL aggregation mechanism to construct a weighted representation of announcement-level embeddings. Each announcement is assigned a learnable importance weight:


$$\begin{array}{c} \alpha_{\text{i},\text{t},\text{j}}=\frac{\text{exp}\left({\text{w}}^{\text{T}}\text{tanh}\left(\text{W}{\text{h}}_{\text{i},\text{t},\text{j}}\right)\right)}{\sum_{\text{k}}\text{exp}\left({\text{w}}^{\text{T}}\text{tanh}\left(\text{W}{\text{h}}_{\text{i},\text{t},\text{k}}\right)\right)} \end{array}$$ (3)

Based on these weights, the firm–month textual representation is computed as:


$$\begin{array}{c} {\text{z}}_{\text{i},\text{t}}=\sum_{\text{j}}\alpha_{\text{i},\text{t},\text{j}}{\text{h}}_{\text{i},\text{t},\text{j}} \end{array}$$ (4)

This mechanism enables the model to automatically focus on announcement content that is more informative for risk assessment, thereby improving the effective utilization of textual information. The aggregated representation ${\text{z}}_{\text{i},\text{t}}$ is then used as input to a nonlinear prediction function to estimate the manipulation risk probability of the firm–month observation:


$$\begin{array}{c} {\hat{y}}_{i,t}={\text{f}}_{\text{text}}\left({\text{z}}_{\text{i},\text{t}}\right) \end{array}$$ (5)

#### 4.1.3. Multi-source fusion model: BERT + MIL + MLP.

Building on the preceding models, this study develops the final fusion model that jointly integrates textual semantic features and structured features. The model first extracts announcement-level semantic representations via BERT combined with MIL aggregation. In parallel, an MLP is used to learn nonlinear mappings of structured numerical variables, including announcement statistics, investor sentiment indicators, and market trading features. These representations are then fused at the feature level to produce the final manipulation risk estimate.

In terms of architecture, the BERT + MIL module maps multiple announcement texts released by firm i in month t into a fixed-dimensional semantic representation vector $z_{i,t}$. This vector is concatenated with the structured numerical feature vector $s_{i,t}$, which covers announcement, sentiment, and market behavior variables, yielding the joint representation:


$$\begin{array}{c} {{\text{x}}^{\prime }}_{\text{i},\text{t}}=\left[{\text{z}}_{\text{i},\text{t}};{\text{s}}_{\text{i},\text{t}}\right] \end{array}$$ (6)

The joint vector is then fed into an MLP prediction module to capture nonlinear relationships. Specifically, the fused representation first passes through fully connected layers for dimensional transformation and is combined with nonlinear activation functions to enhance the model’s capacity to represent complex patterns. The model outputs a manipulation risk probability for firm i in month t as:


$$\begin{array}{c} {\hat{y}}_{i,t}=\sigma \left({\text{f}}_{\text{MLP}}\left({{\text{x}}^{\prime }}_{\text{i},\text{t}}\right)\right) \end{array}$$ (7)

where σ (⋅) denotes the sigmoid function (Sigmoid) that maps the output to the [0,1] interval.

In the empirical implementation, the 15 structured variables are projected into a 64-dimensional ReLU-activated representation and concatenated with the 768-dimensional BERT–MIL text representation. The fused vector is passed through a 128-dimensional ReLU-activated hidden layer, followed by a sigmoid output layer, and the model is trained for 20 epochs using AdamW with a learning rate of 2e-3. We then systematically compare this fusion model with the Logit baseline, the CatBoost classifier, and the text-only BERT + MIL model, evaluating the practical value of multi-source information fusion in manipulation risk assessment in terms of predictive performance and stability.

### 4.2. Empirical analysis

#### 4.2.1. Sample and data sources.

This study focuses on listed firms in China’s A-share market and constructs a firm–month supervised learning dataset for stock market manipulation risk assessment. Violating firms are identified based on administrative penalty cases for market manipulation publicly disclosed by the CSRC between 2022 and 2024. Using the violation intervals specified in the penalty documents, manipulation behaviors are retrospectively mapped to the firm–month level to generate risk labels. Because regulatory disclosure typically lags behind the occurrence of violations, the backdated sample months are primarily distributed between 2015 and 2023, corresponding to the actual periods in which the misconduct occurred. A firm i is labeled as $Y_{i,t}=\text{1}$ if it falls within a violation interval during month t. The sanction records are used ex post to define supervised learning labels and are not used as explanatory variables in model training. The model is therefore interpreted as learning observable public-data patterns associated with retrospectively identified violation months and producing periodic manipulation-risk scores.

To ensure sample comparability, each violating firm–month observation is matched with five non-violating firm–month observations using nearest-neighbor matching within the same month and industry. Industry categories follow the CSRC classification standards, and nearest-neighbor distance is calculated based on log market capitalization. The 1:5 ratio is adopted to retain sufficient comparable control observations while keeping class imbalance at a manageable level for the supervised risk assessment task. Matching quality is assessed through covariate balance diagnostics at the matched-sample construction stage. Since month and industry are exactly matched by construction, the diagnostics focus on firm size. As shown in Panel B of Table 2, the absolute standardized mean differences are 0.0320 for log market capitalization and 0.0206 for raw market capitalization, both below the conventional 0.1 threshold, indicating good balance on the observed size-related matching covariates.

**Table 2:** Sample distribution and matching diagnostics.

Panel A. Distribution of market manipulation violations by year.				
**Year**	**Violating observations**	**Total observations**	**Violation ratio**	
**2015**	33	173	19.10%	
**2016**	25	175	14.30%	
**2017**	99	609	16.30%	
**2018**	87	540	16.10%	
**2019**	83	456	18.20%	
**2020**	89	549	16.20%	
**2021**	47	271	17.30%	
**2022**	30	138	21.70%	
**2023**	3	32	9.40%	
**Panel B. Covariate balance diagnostics for matched-sample construction.**				
**Covariate**	**Violating mean**	**Matched control mean**	**SMD**	**p-value**
**Log market capitalization**	14.8456	14.8750	-0.0320	0.4408
**Market capitalization**	4,646,140	4,783,079	-0.0206	0.6124

**Note:** The violation counts in Panel A represent firm–month observations associated with market manipulation cases administratively sanctioned by the CSRC between 2022 and 2024. These observations are retrospectively assigned to the years in which the violations actually occurred. The violation ratio refers to the proportion of violating firm–month observations relative to the full sample in each year.

**Note:** Panel B reports covariate balance diagnostics at the matched-sample construction stage. Matching is performed within the same month and industry, and the nearest-neighbor distance is calculated based on log market capitalization. Since month and industry are exactly matched by construction, the diagnostics focus on size-related covariates. SMD denotes standardized mean difference. The p-values test mean differences between violating and matched control observations.

Regarding data sources, this study integrates multi-source heterogeneous data to characterize market manipulation risk from disclosure, sentiment, and trading dimensions. Corporate announcement data are obtained from the Choice Financial Terminal (Choice) and include both periodic reports and ad hoc disclosures released during the study period, reflecting firms’ formal information disclosure activities. Investor sentiment data are collected from the Eastmoney Guba platform, a retail-oriented online forum, and are used as proxies for retail-facing public opinion dynamics, investor attention, and informal information dissemination. Market trading data are sourced from the China Stock Market and Accounting Research Database (CSMAR), covering indicators such as monthly returns, turnover rates, and market capitalization changes to describe firms’ market trading behavior.

Through this sample construction and data integration process, the study develops a multi-source panel dataset at the monthly level that combines corporate disclosures, sentiment dynamics, and market trading behavior, thereby providing a robust data foundation for the subsequent modeling and empirical analysis of stock market manipulation risk.

#### 4.2.2. Empirical results analysis.

(1) Descriptive Analysis

**Fig 2:**
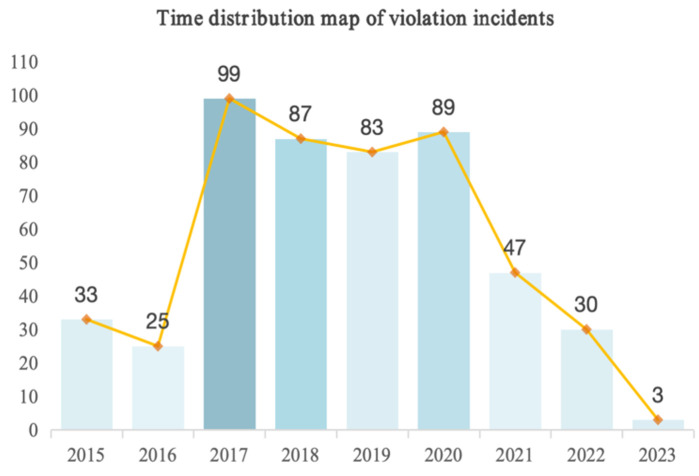
Temporal distribution of violating observations.

We begin by examining the annual distribution of violating firm–month observations, as presented in Fig 2 and Table 2. It is important to clarify that the reported violation counts are measured at the firm–month level rather than by the number of manipulation cases. The sample is constructed based on administrative penalty announcements issued by the CSRC during 2022–2024. Using the violation intervals disclosed in the penalty documents, manipulation behaviors are retrospectively mapped to the months in which they occurred. Consequently, a single case spanning multiple calendar months corresponds to multiple violating observations. From a temporal perspective, violating observations are relatively concentrated between 2017 and 2020. This concentration reflects the enforcement-based sample design rather than the market-wide incidence of manipulation. Sanctioned cases often involve persistent, stage-like conduct, with violation intervals spanning multiple months. Since the sample is defined by CSRC penalty announcements issued during 2022–2024 and backdated to the disclosed violation periods, the observed distribution represents the temporal coverage of sanctioned cases within this sample.

**Fig 3:**
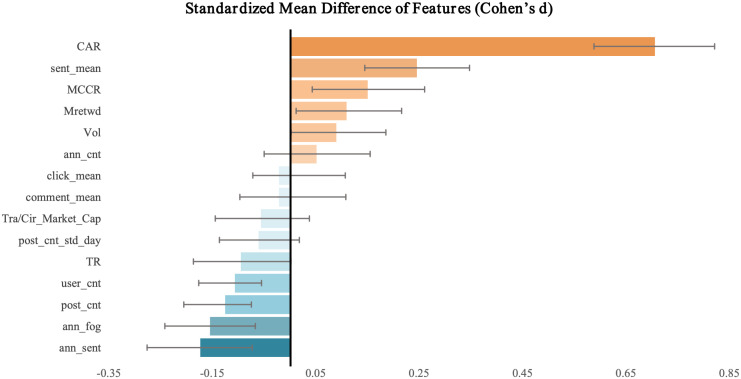
Standardized mean differences with 95% confidence intervals.

To characterize the direction, magnitude, and statistical uncertainty of group differences, Fig 3 reports standardized mean differences measured by Cohen’s d with bootstrap 95% confidence intervals. This metric removes the influence of scale and allows the 15 variables to be compared on a common basis. Positive values indicate higher feature means among violating observations, whereas negative values indicate lower means. Confidence intervals that exclude zero indicate statistically distinguishable differences between the two groups.

As shown in Fig 3, market trading variables provide the strongest univariate contrasts. CAR has the largest positive standardized difference, followed by MCCR, Mretwd, and Vol, indicating that violating observations are more strongly associated with abnormal return movements, market value changes, and price fluctuations. Announcement and sentiment variables show more moderate marginal differences. The negative differences in ann_sent and ann_fog indicate lower standardized means among violating observations, suggesting that disclosure-related signals differ across risk states even when their univariate magnitudes are smaller. This pattern implies that these disclosure and sentiment signals may emerge more prominently through interactions with trading features rather than as standalone predictors. This interpretation is further supported by the performance gains observed in the fusion model and by the SHapley Additive exPlanations (SHAP) results discussed later.

**Fig 4:**
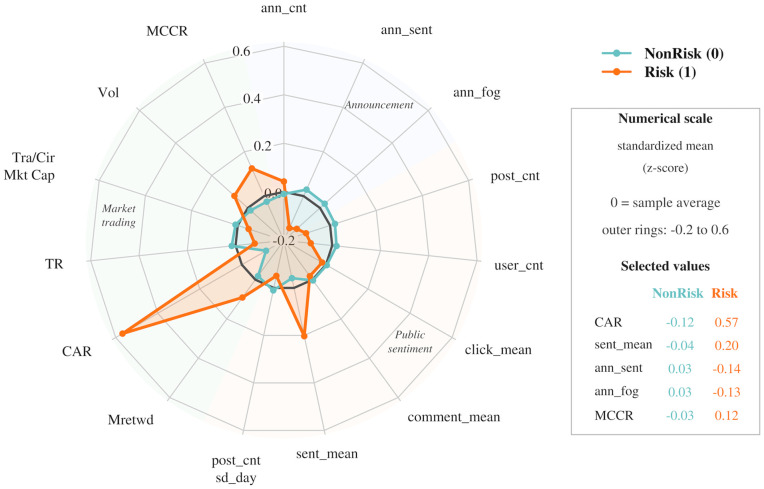
Radar profile of standardized feature means by risk state.

Fig 4 presents the standardized feature profiles of violating and non-violating observations within the multidimensional feature space. All variables are standardized on a common z-score scale, where zero denotes the sample average. Positive values indicate above-average feature levels, whereas negative values indicate below-average levels. The overall profile indicates that violating observations exhibit an outward shift along market trading dimensions, particularly around CAR, MCCR, and Vol. This pattern is consistent with the view that market manipulation is frequently accompanied by abnormal price and trading responses. By contrast, non-violating observations display a more concentrated distribution across most indicators, reflecting comparatively stable trading conditions. Differences in announcement tone, textual complexity, and public sentiment are less pronounced in magnitude, but they remain visible as part of the broader multidimensional profile. Together with the effect-size evidence in Fig 3, this pattern suggests that manipulation risk is better represented by the joint configuration of market response, disclosure characteristics, and sentiment dynamics than by a single class of variables alone.

(2) Model Comparison Analysis

To reduce dependence on a particular random partition, all random split experiments are repeated using five random seeds, and Table 3 reports model performance as mean ± standard deviation. The same random partitions are used across models, which makes the model comparisons paired at the seed level. Rather than relying only on point estimates, Table 4 further examines whether the performance differences between the final fusion model and each comparison model are statistically supported under this paired design. The relatively small standard deviations of the fusion model, particularly for AUC, PR-AUC, and Recall@20%, indicate that its performance is not highly sensitive to a particular random split.

**Table 3:** Comparative evaluation of model performance.

Model	Input	AUC	PR-AUC	Recall@10%	Recall@20%
**Logit**	Numerical features	0.6984 ± 0.0268	0.3549 ± 0.0329	0.2646 ± 0.0374	0.4202 ± 0.0332
**CatBoost**	Numerical features	0.7768 ± 0.0389	0.5130 ± 0.0562	0.3394 ± 0.0382	0.4949 ± 0.0647
**BERT + MIL**	Announcement text	0.8296 ± 0.0286	0.6464 ± 0.0464	0.4384 ± 0.0444	0.6323 ± 0.0402
**BERT + MIL + MLP**	Announcement text + numerical features	0.8811 ± 0.0063	0.6943 ± 0.0158	0.4545 ± 0.0294	0.6788 ± 0.0166

**Note:** Values are reported as mean ± SD across five random seeds. The same random partitions are used across models.

**Table 4:** Paired significance tests based on repeated random seeds.

Comparison	AUC diff.	AUC p-value	PR-AUC diff.	PR-AUC p-value	Recall@10% diff.	Recall@10% p-value	Recall@20% diff.	Recall@20% p-value
**Fusion vs Logit**	0.1827	<0.001	0.3394	<0.001	0.1899	0.0026	0.2586	<0.001
**Fusion vs CatBoost**	0.1043	0.0028	0.1813	0.0018	0.1152	0.0079	0.1838	0.0023
**Fusion vs BERT+MIL**	0.0515	0.0091	0.0478	0.0388	0.0162	0.4382	0.0465	0.0303

**Note:** This table reports seed-level paired t-tests based on the same five repeated random-seed experiments used in Table 3. For each metric, the difference is calculated as the performance of the final fusion model minus that of the comparison model under the same seed. The reported diff. is the average paired difference, and the p-value tests whether the mean paired difference differs from zero.

The results reveal a clear progression in risk assessment performance as the model input moves from structured numerical variables to announcement semantics and then to multi-source fusion. As shown in Table 3, the Logit model serves as an interpretable baseline, but its relatively low PR-AUC and Recall@K indicate limited ability to prioritize the minority high-risk observations. Under the same structured-feature input, CatBoost improves the average performance, suggesting that nonlinear relationships and feature interactions among the numerical variables contain additional risk information that cannot be fully captured by a linear specification.

The BERT + MIL model uses announcement texts as the sole input and does not rely on the 15 manually constructed numerical indicators. Its performance indicates that raw announcement texts contain risk-relevant semantic information that is not fully represented by aggregate textual indicators such as announcement frequency, lexicon-based tone, or readability. This result is consistent with the role of corporate disclosures as a formal information channel through which firms communicate events, uncertainty, and risk-related information to the market. The MIL structure is also appropriate for the firm–month setting because multiple announcements may be released within the same month and may differ substantially in their relevance to risk assessment. By treating these announcements as an information set, the model can aggregate unevenly informative textual signals at the firm–month level.

Building on this foundation, the final BERT + MIL + MLP model combines the announcement-text representation with the 15 structured numerical indicators and achieves the highest average performance across all four metrics. The paired tests provide statistical support for the improvements over the two structured-feature models, suggesting that these gains are unlikely to be driven solely by random split variation. Relative to the pure-text BERT + MIL model, the evidence is more nuanced. The fusion model shows statistically significant improvements in AUC, PR-AUC, and Recall@20%, whereas the improvement in Recall@10% is not statistically significant. This pattern suggests that multi-source fusion mainly improves overall ranking quality and broader high-risk coverage, while its additional contribution is less pronounced within the most restrictive Top 10% screening threshold.

Overall, the model comparison supports the complementary value of combining raw announcement texts with structured disclosure, sentiment, and trading indicators. Announcement texts provide semantic information about how firms disclose and organize risk-related information, whereas structured sentiment and trading variables describe public attention, information diffusion, and market response. Their integration yields a more informative risk ranking for the screening-oriented objective of this study.

To complement the aggregate performance metrics, we further examine the screening utility of the final fusion model under capacity-based review thresholds. Test observations are ranked by risk scores assigned by the model, and the top 5%, 10%, and 20% are treated as candidate review sets. This analysis reports the number of firm–month observations selected for review, the number of true and false positives, precision, recall, and the false discovery rate. In this way, the model output is evaluated in terms of the practical trade-off among review burden, risk coverage, and false positive burden.

Table 5 shows that the screening threshold materially affects the balance between risk coverage and false positive burden. A stricter Top 5% threshold yields high precision and a low false discovery rate, whereas expanding the review set to Top 20% substantially improves recall but also increases the false discovery rate. The Top 10% threshold provides an intermediate setting, capturing nearly half of the true risk observations while keeping the false discovery rate below 25%. These results provide a quantitative basis for review prioritization under limited regulatory resources using the risk scores assigned by the model.

**Table 5:** Screening utility under capacity-based review thresholds.

Screening threshold	Reviewed observations	True positives	False positives	Precision	Recall	False discovery rate
**Top 5%**	29	26.4 ± 0.9	2.6 ± 0.9	0.9103 ± 0.0308	0.2667 ± 0.0090	0.0897 ± 0.0308
**Top 10%**	59	45.0 ± 2.9	14.0 ± 2.9	0.7627 ± 0.0494	0.4545 ± 0.0294	0.2373 ± 0.0494
**Top 20%**	118	67.2 ± 1.6	50.8 ± 1.6	0.5695 ± 0.0139	0.6788 ± 0.0166	0.4305 ± 0.0139

**Note:** Except for reviewed observations, values are mean ± SD across five random seeds. Reviewed observations are firm–month observations. False discovery rate = false positives/ reviewed observations.

(3) SHAP Interpretability Analysis

**Fig 5:**
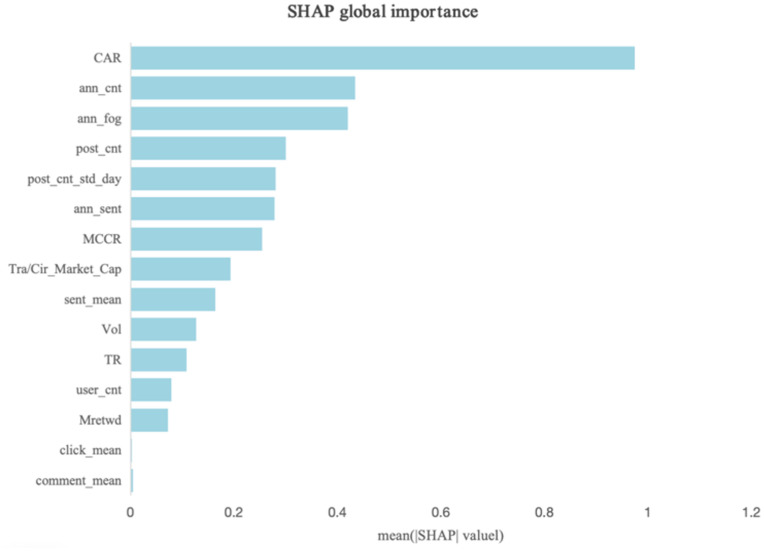
Global feature importance based on SHAP.

**Fig 6:**
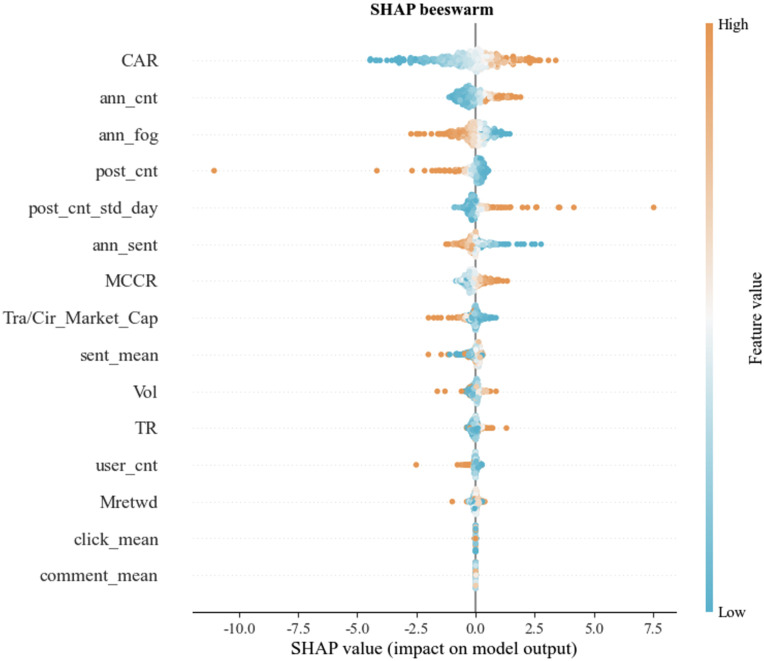
SHAP feature effects on manipulation risk.

**Fig 7:**
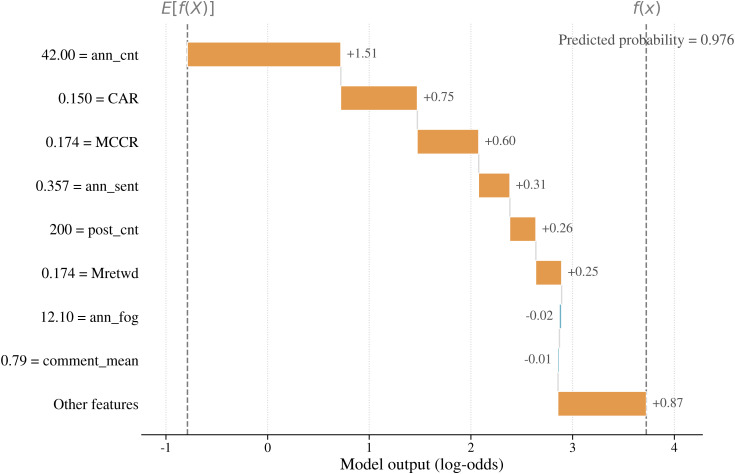
Local SHAP explanation for a high-risk observation.

To examine how the structured numerical variables contribute to the final fusion model, this study applies SHapley Additive exPlanations (SHAP) to interpret the numerical branch of the BERT + MIL + MLP model. Fig 5 reports the global feature importance based on the mean absolute SHAP value; Fig 6 illustrates the direction and heterogeneity of feature effects; and Fig 7 provides a local explanation for a representative high-risk observation.

As shown in Fig 5, the CAR variable ranks first in the global importance results, indicating that abnormal return deviations provide the strongest structured signal for the model’s risk scoring. This finding is consistent with the role of price responses in market manipulation cases, where abnormal returns often represent an observable market-level manifestation of risk. Announcement volume (ann_cnt) and announcement textual complexity (ann_fog) rank immediately after CAR, suggesting that formal disclosure behavior provides substantial information in addition to market trading variables. Sentiment-related indicators, including posting volume (post_cnt) and the daily variability of posting activity (post_cnt_std_day), also appear among the important features, suggesting that the model assigns greater importance to the intensity and temporal clustering of investor attention than to passive information exposure. These results indicate that the fusion model captures not only abnormal price movements, but also variation in corporate disclosure behavior and the concentration of investor attention.

To further examine whether the fusion model changes the contribution pattern of structured variables, we compare normalized SHAP importance shares between CatBoost and the structured numerical branch of the fusion model. The comparison is restricted to the 15 shared structured variables, because the BERT textual representation cannot be directly decomposed into financially interpretable variables. Market trading features remain the largest structured feature group in both models, although their normalized share decreases from 0.504 in CatBoost to 0.469 in the fusion model. Announcement features show the largest relative increase, rising from 0.178 to 0.307, while public sentiment features decrease from 0.318 to 0.224. This redistribution suggests that the fusion model does not simply reproduce the CatBoost importance structure. Instead, after announcement-text representations are incorporated, structured disclosure variables gain relative importance, whereas part of the contribution previously assigned to sentiment and trading variables is absorbed into a more balanced multi-source representation.

Fig 6 further illustrates the directionality and heterogeneity of feature effects. Along the CAR dimension, observations with high CAR values are mainly distributed on the positive SHAP side, indicating that larger abnormal return deviations tend to increase the risk score assigned by the model. By contrast, announcement textual complexity (ann_fog) and announcement sentiment (ann_sent) show more conditional patterns. Higher values of these disclosure-derived indicators are more often associated with negative SHAP contributions, but their distributions are not strictly one-sided and the effects can vary across observations. This suggests that textual complexity and announcement tone should not be interpreted as mechanical risk amplifiers or risk reducers. Their contributions depend on whether changes in readability or tone coincide with abnormal trading activity and sentiment diffusion. Sentiment variables also display nonlinear effects, with extreme observations contributing more strongly to the risk score in some cases. This pattern indicates that abrupt shifts in information diffusion may serve as risk-relevant cues in the model’s assessment.

Fig 7 illustrates this logic at the observation level. The selected true-positive firm–month observation receives a high predicted risk probability of 0.976. Its local explanation shows that the risk score is raised by features from multiple dimensions, including announcement volume, CAR, market capitalization change, announcement sentiment, posting volume, monthly return, and trading value relative to circulating market capitalization. At the same time, several variables contribute in the opposite direction, indicating that the final risk score results from the balance of reinforcing and offsetting signals rather than from a single feature. This local explanation helps translate the global SHAP results into an observation-level risk profile and illustrates how the model can support review prioritization in a screening context.

Overall, the SHAP results provide three implications for manipulation risk assessment. First, price response variables remain important, but the model’s risk assessment output is not limited to a single market indicator. Second, disclosure behavior and sentiment diffusion provide complementary information when they evolve jointly with market trading patterns. Third, the local explanation shows how a high-risk score can be traced back to a structured combination of feature contributions. From a regulatory perspective, this suggests that risk screening should not rely only on price anomalies, but should also monitor whether abnormal returns coincide with unusual disclosure intensity, shifts in disclosure tone and readability, and concentrated investor attention. Given the high importance of CAR in the SHAP results, Section 4.3.2 further examines the robustness of the fusion model after excluding CAR from the structured numerical feature set.

### 4.3. Robustness and additional validation

This section evaluates whether the main findings are sensitive to key measurement and specification choices. Specifically, we examine the robustness of the sentiment measurement, the contribution of CAR to the fusion model, and the temporal validity of the proposed framework.

#### 4.3.1. Alternative sentiment measurement.

The sentiment score in Equation (1) is constructed from a Chinese financial sentiment lexicon and serves as an interpretable proxy for announcement tone. Measures based on lexicons are transparent and reproducible, but their representation of contextual meaning depends on dictionary coverage and word usage. To assess whether the empirical results are sensitive to this measurement choice, we construct alternative sentiment variables using a Chinese transformer sentiment classifier motivated by the FinBERT framework [47].

The classifier produces sentiment class probabilities, and the alternative sentiment score is defined as P(positive) − P(negative). The resulting sentiment scores are aggregated to the firm–month level and used to replace the original announcement sentiment variable (ann_sent) and investor forum sentiment variable (sent_mean). All remaining structured variables, as well as the sample, labels, announcement texts, and fusion model architecture, remain unchanged. The replacement affects only the structured sentiment variables and does not alter the announcement representation module of the fusion model.

As reported in Table 6, the fusion model retains comparable performance under the alternative sentiment specification. Across five random seeds, the alternative specification achieves an average AUC of 0.8813, a PR-AUC of 0.6853, Recall@10% of 0.4505, and Recall@20% of 0.6889. These results are close to those of the main specification reported in Table 3, indicating that the empirical findings are not materially driven by the specific lexicon-based construction of the sentiment variables.

**Table 6:** Robustness check using alternative sentiment measurement.

Specification	Sentiment measurement	AUC	PR-AUC	Recall@10%	Recall@20%
**Main specification**	Chinese financial sentiment lexicon	0.8811 ± 0.0063	0.6943 ± 0.0158	0.4545 ± 0.0294	0.6788 ± 0.0166
**Alternative specification**	Chinese transformer sentiment classifier	0.8813 ± 0.0088	0.6853 ± 0.0591	0.4505 ± 0.0477	0.6889 ± 0.0330

**Note:** The main specification is repeated from Table 3 for reference. In the alternative specification, ann_sent and sent_mean are replaced by sentiment measures generated using a Chinese transformer sentiment classifier. Other structured variables, labels, announcement texts, and fusion model architecture remain unchanged.

#### 4.3.2. Feature ablation of CAR.

The SHAP analysis identifies cumulative abnormal returns (CAR) as the most influential structured trading feature. To assess the incremental role of this price response variable, we conduct a feature ablation by removing CAR from the structured numerical feature set. The sample, labels, announcement texts, remaining structured variables, and model architecture are kept unchanged.

Table 7 reports the results of the CAR ablation analysis. After CAR is removed, model performance declines across all evaluation metrics. The average AUC decreases from 0.8811 to 0.8370, PR-AUC decreases from 0.6943 to 0.5743, Recall@10% decreases from 0.4545 to 0.3818, and Recall@20% decreases from 0.6788 to 0.6121. These results indicate that CAR provides substantial information for manipulation risk assessment and constitutes an important component of the market trading feature group.

**Table 7:** Feature ablation analysis excluding CAR.

Specification	Feature setting	AUC	PR-AUC	Recall@10%	Recall@20%
**Main specification**	Full feature set	0.8811 ± 0.0063	0.6943 ± 0.0158	0.4545 ± 0.0294	0.6788 ± 0.0166
**CAR excluded specification**	CAR removed from structured numerical features	0.8370 ± 0.0236	0.5743 ± 0.0591	0.3818 ± 0.0522	0.6121 ± 0.0382

**Note:** The main specification is repeated from Table 3 for reference. In the CAR exclusion specification, cumulative abnormal returns are removed from the structured numerical feature set. The sample, labels, announcement texts, remaining structured variables, and fusion model architecture remain unchanged.

Despite this decline, the CAR exclusion specification continues to show risk ranking capacity, with an AUC of 0.8370 and a Recall@20% of 0.6121. This suggests that announcement semantics, disclosure characteristics, sentiment variables, and the remaining trading indicators still contain useful screening information after CAR is removed. Therefore, the full model should be interpreted as a multi-source risk assessment framework in which abnormal return information improves model performance, while textual, sentiment, and other trading signals also contribute to the final risk assessment output.

#### 4.3.3. Temporal validation.

To complement the repeated random split evaluation with a chronological design, this study further conducts a temporal validation. The models are trained on observations from 2015 to 2020, validated on observations from 2021, and evaluated on observations from 2022 to 2023. By preserving the calendar order of the sample, this design assesses the temporal generalizability of the risk assessment framework.

Table 8 reports the temporal validation results. The fusion model achieves the highest AUC and PR-AUC, with values of 0.8903 and 0.7096, respectively. It also obtains the highest Recall@20% of 0.7273, while its Recall@10% is equal to that of the Logit model. Although CatBoost attains a competitive AUC, its lower PR-AUC and Recall@K indicate weaker performance in prioritizing observations with elevated risk. The BERT + MIL model using only announcement text performs less favorably under the chronological split, suggesting that announcement semantics alone are more sensitive to temporal changes than the fused representation.

**Table 8:** Temporal validation results.

Model	Input	AUC	PR-AUC	Recall@10%	Recall@20%
**Logit**	Numerical features	0.8308	0.5767	0.3939	0.6364
**CatBoost**	Numerical features	0.8693	0.5452	0.2727	0.5758
**BERT + MIL**	Announcement text	0.7144	0.4261	0.2121	0.3333
**BERT + MIL + MLP**	Announcement text + numerical features	0.8903	0.7096	0.3939	0.7273

**Note:** The chronological split uses 2015–2020 as the training period, 2021 as the validation period, and 2022–2023 as the test period. Metrics are reported on the temporal test period.

Overall, the temporal validation provides complementary evidence for the stability of the multi-source framework. While the repeated random split results evaluate average model performance under alternative sample partitions, the chronological split examines whether the learned risk patterns remain informative when the evaluation period moves forward in time. The results show that combining announcement semantics, public sentiment, and market trading signals preserves effective risk ranking ability in this temporal setting. Thus, the chronological validation should be interpreted as a complementary assessment of temporal generalizability, whereas the repeated random split results in Table 3 are reported as an average-performance benchmark for model comparison.

## 5. Discussion

### 5.1. Conceptual and theoretical implications

The results support a multi-source conceptualization of stock market manipulation risk at the firm–month level. Manipulation-related risk is not fully captured by isolated trading anomalies, nor is it adequately represented by disclosure or sentiment indicators alone. Rather, elevated risk appears as a configuration of observable public signals spanning formal disclosure behavior, public sentiment diffusion, and market trading responses. This perspective moves the analysis beyond single-source abnormality detection and toward a structured representation of risk across multiple information channels.

This view is especially relevant for screening-oriented regulatory settings. Manipulation strategies may differ substantially in their transaction-level mechanisms and observable manifestations, and trade-based, information-based, and combined manipulation patterns are unlikely to generate identical empirical signatures. A single indicator is therefore unlikely to capture their common risk features. By organizing disclosure, sentiment, and trading variables within a unified firm–month framework, this study treats manipulation-related risk as a probabilistic and multidimensional state rather than as the deterministic outcome of one causal channel. The relevant question is whether multiple public signals jointly form a risk profile that warrants further review.

At the conceptual level, the study frames manipulation-risk assessment as a problem of public-signal representation. This does not diminish the importance of transaction-level evidence for enforcement decisions. Instead, it clarifies the complementary role of multi-source risk scoring in the earlier stages of regulatory monitoring. The proposed framework connects the information-based logic of disclosure, sentiment diffusion, and market response with the regulatory need to prioritize attention under limited review capacity.

### 5.2. Methodological and empirical implications

Methodologically, the results show the value of combining textual representations with structured numerical indicators. The comparison among Logit, CatBoost, BERT + MIL, and the final BERT + MIL + MLP model indicates that different information representations capture different aspects of manipulation-related risk. Structured-feature models provide interpretable signals from disclosure characteristics, public sentiment, and market trading variables, whereas the text-only BERT + MIL model captures semantic information embedded in corporate announcements. The stronger performance of the fusion model suggests that announcement semantics and structured indicators are complementary rather than redundant for firm–month risk ranking.

The paired significance tests further refine this interpretation. The fusion model shows consistent improvements over the structured-feature baselines across AUC, PR-AUC, Recall@10%, and Recall@20%. Compared with the text-only model, the fusion model shows clearer gains in AUC, PR-AUC, and Recall@20% than in the most restrictive Recall@10% setting. This pattern suggests that multi-source fusion contributes more strongly to overall ranking quality and broader high-risk coverage than to incremental gains within the narrowest review capacity. This is consistent with the screening objective of the study, where model utility depends not only on identifying the very highest-risk observations, but also on improving prioritization across different review capacities.

The additional validation analyses further clarify the robustness and interpretation of the framework. The alternative sentiment measurement indicates that the main findings are not materially driven by the lexicon-based construction of sentiment variables. The CAR ablation is particularly informative because CAR is the strongest price-response variable in the structured feature set. Although removing CAR reduces model performance, the model still achieves an AUC of 0.8370 and a Recall@20% of 0.6121, indicating that the framework retains meaningful risk-ranking capacity even when the most influential abnormal-return signal is excluded. This result suggests that the model is not reducible to a simple price-momentum or abnormal-return detector. The chronological validation further shows that the multi-source framework remains informative in later sample periods, although temporal performance differences may reflect changes in market conditions, information environments, and regulatory enforcement patterns over time. In this context, regulatory enforcement patterns refer to changes in the CSRC’s enforcement priorities, enforcement intensity, and the timing or level of detail in public disclosures of enforcement cases. Because the outcome labels are derived from ex post CSRC penalty documents, such institutional changes may affect the composition and observability of sanctioned cases used for label construction, rather than indicating that the model directly measures regulatory activity.

### 5.3. Regulatory implications and boundary conditions

The results also indicate how multi-source risk scores can support risk-based financial supervision. In regulatory practice, supervisory resources are limited and cannot be allocated uniformly across all listed firms and time periods. A firm–month risk score can serve as a front-end screening tool by converting heterogeneous public signals into a ranked list of observations for further review. The screening utility analysis shows how different review thresholds correspond to different combinations of review burden, risk coverage, precision, and false discovery rates. In this sense, the model helps make the trade-off between coverage and review burden explicit, rather than replacing regulatory investigation.

The model output should be interpreted as a candidate-review signal rather than as direct evidence of manipulation. A high predicted risk score indicates that a firm–month observation exhibits a combination of disclosure, sentiment, and trading patterns similar to those observed in sanctioned manipulation periods. Such a signal can help allocate regulatory attention, but it should be assessed together with firm-specific events, industry conditions, market-wide shocks, contextual information, and expert judgment. This distinction is important because legitimate information shocks or sector-level fluctuations may also generate abnormal returns, sentiment bursts, and trading activity. Practical use of the model should therefore emphasize tiered review and human verification rather than automatic enforcement decisions.

Several boundary conditions should be recognized. First, the labels are constructed from ex post CSRC penalty documents, so the model learns patterns associated with detected and sanctioned manipulation cases rather than the full population of manipulation activities. Second, the firm–month structure is suitable for periodic screening but may smooth short-horizon manipulation episodes that occur within days or around specific events. Third, the model does not incorporate transaction-level order or trade records and therefore cannot identify specific manipulative actions or actors. These limitations do not undermine the screening-oriented objective of the study, but they define the appropriate scope of interpretation. Future research could extend the framework by incorporating higher-frequency trading records, event-level annotations, investor-structure information, and cross-market evidence to further evaluate the generalizability and operational value of multi-source manipulation-risk assessment.

## 6. Conclusion

This study develops a multi-source framework for firm–month stock market manipulation risk assessment by integrating corporate announcement texts, online public sentiment indicators, and market trading variables. Based on CSRC-sanctioned manipulation cases retrospectively mapped to monthly violation intervals, the study evaluates whether heterogeneous public signals can support probabilistic risk ranking and tiered regulatory screening. The empirical results show that the BERT + MIL + MLP fusion model outperforms structured-feature and text-only baselines in overall discrimination and high-risk coverage. Additional analyses based on alternative sentiment measurement, CAR ablation, and chronological validation further support the robustness and screening utility of the proposed framework.

Taken together, the findings suggest that manipulation-related risk is better represented as a configuration of observable public signals than as a single abnormal indicator. The proposed framework provides a data-driven basis for transforming disclosure, sentiment, and trading information into firm–month risk scores for candidate review. These scores should be interpreted as screening signals rather than transaction-level evidence of manipulation and should be used together with contextual verification and regulatory judgment. Future work can extend this screening-oriented framework using higher-frequency, transaction-level, and cross-market data.

## Supporting information

S1 Data Processed relevant firm–month numerical dataset used in the empirical analysis. This ZIP file contains relevant data.xlsx, which includes the processed firm–month level numerical variables constructed from corporate disclosure, public sentiment, and market trading dimensions. Restricted raw data from third-party providers are not included.
(ZIP)
