# Supplementary material for: Risk assessment of stock market manipulation through the fusion of multi-source textual and trading data: Evidence from China’s A-share market
Source: PLoS One. 2026 Aug 7;21(8):e0343442. doi: 10.1371/journal.pone.0343442 (PMC13450784; doi:10.1371/journal.pone.0343442)
Supplement: Supplementary file 2 [file pone.0343442.s004.docx]

**Response to Reviewers**

Manuscript number: PONE-D-26-06350

Title: Risk assessment of stock market manipulation through the fusion of multi-source textual and trading data: Evidence from China’s A-share market

**Dear** **Editor and Reviewers,**

Thank you very much for the opportunity to revise our manuscript entitled “Risk assessment of stock market manipulation through the fusion of multi-source textual and trading data: Evidence from China’s A-share market” (PONE-D-26-06350). We sincerely appreciate the editor’s and reviewers’ careful evaluation, constructive comments, and valuable suggestions.

We have treated these comments as an important opportunity to improve the manuscript. In the revised version, we have made substantial revisions to the literature review, theoretical framework, variable construction rationale, empirical methodology, model implementation details, robustness analyses, interpretability analysis, practical implications, and limitations. These revisions have helped us clarify the scope of the study, strengthen the connection between theory and empirical design, and present the proposed framework more cautiously as a screening-oriented risk assessment tool rather than as a transaction-level manipulation detection system.

We have responded to each comment point by point below. For clarity, the reviewers’ comments, our responses, and the corresponding amendments in the manuscript are presented separately. We have also marked the revised parts in the manuscript using colored text to facilitate review.

We are grateful for the time and effort that the editor and reviewers have devoted to evaluating our work. We hope that the revised manuscript and the accompanying responses adequately address the concerns raised, and we respectfully submit the revised manuscript for further consideration.

**Response to Editor:**

Thank you for the editorial guidance and for the opportunity to revise our manuscript. We have carefully addressed the editorial requirements and the reviewers’ comments in the revised manuscript and in this point-by-point response letter.

First, we have revised the manuscript substantially in response to the methodological and theoretical concerns raised during review. In particular, we strengthened the literature review, clarified the theoretical positioning of the study, refined the variable construction rationale, added methodological details for reproducibility, expanded the robustness and validation analyses, improved the SHAP-based interpretability analysis, and clarified the practical scope and limitations of the proposed framework.

Second, regarding data availability, we have provided the processed relevant data file, relevant data.xlsx, as supporting information. This file contains the firm–month level numerical variables constructed and used in the empirical analysis, including the 15 structured features derived from corporate disclosure, public sentiment, and market trading dimensions. We provide these processed variables to improve the transparency of the empirical analysis to the extent permitted.

At the same time, the raw data used in this study cannot be publicly redistributed. The raw corporate announcement files and related disclosure data were obtained from Choice Financial Terminal (East Money Information Co., Ltd.) (official website: https://choice.eastmoney.com/). The market trading data were sourced from the CSMAR Database (GTA Information Technology Co., Ltd.) (official website: https://data.csmar.com/). The online public opinion data, including raw Eastmoney Guba posts, were collected from Eastmoney Guba (East Money Information Co., Ltd.) (official website: https://guba.eastmoney.com/). All these data are subject to the access conditions and redistribution restrictions of the original data providers. Qualified researchers may contact the respective providers via the above official websites to inquire about data access.

Third, regarding code availability, we respectfully clarify that the complete project code cannot be publicly released due to project confidentiality requirements. The codebase contains project-specific workflows and system-development components associated with the funded regulatory technology project. We understand the journal’s emphasis on reproducibility. To improve transparency as much as possible within these constraints, we have added detailed methodological descriptions in the revised manuscript, including the construction of CAR and volatility, the Chinese-adapted textual complexity measure, the BERT model used, the BERT encoder setting, the MLP architecture, optimizer, learning rate, number of epochs, class-weighting strategy, repeated random-split design, paired significance tests, chronological validation, and robustness checks.

Fourth, regarding the role of the funding project, the project supported data acquisition and provided the broader regulatory application context. The authors independently conducted the empirical analysis, interpreted the results, prepared the manuscript, and made the decision to submit the work for publication. The potential future use of the research results for system development does not affect the interpretation of the empirical findings reported in the manuscript.

Fifth, regarding the Supporting Information file, the processed dataset submitted in the previous round is retained in this revised submission. Because this file is provided to support data availability rather than as a separately discussed analytical result, we do not introduce an additional in-text discussion of the file. We have added a Supporting Information caption at the end of the manuscript to describe the file clearly and consistently.

Finally, we have revised the manuscript to clarify the practical scope of the study. The proposed model is not presented as an automatic enforcement tool or transaction-level manipulation detector. Instead, it is positioned as a screening-oriented risk assessment framework that produces probabilistic risk scores for candidate review and prioritization. We have also clarified the limitations related to retrospective regulatory labels, false positive risk, lack of transaction-level order data, and generalizability beyond China’s A-share market.

We hope that these revisions and clarifications address the editorial requirements and improve the transparency, rigor, and practical interpretability of the manuscript.

Response to Reviewer 1:	2

Response to Reviewer 2:	20

Response to Reviewer 3:	58

Response to Reviewer 1:

**Comment 1:**

*In the introduction although it recognizes that with the increasing complexity of stock manipulation issues you get too generic to have so many conversations about these issues. And you should definitely explain different manipulation types (e.g. trade-based vs. information-based) and to position your research in that perspective.*

**Response 1:**

Thank you for this important and constructive suggestion. We agree that the original Introduction discussed the increasing complexity of stock market manipulation in a relatively general manner and did not sufficiently position the study with respect to different manipulation types.

In the revised manuscript, we have clarified this issue in both the Introduction and Section 3.1. Specifically, following the reviewer’s suggestion, we now explain that stock market manipulation may involve trade-based forms, information-based forms, and combined forms in which trading behavior and information dissemination interact. This revision helps position the study more clearly within the manipulation literature and makes clear that different manipulation forms may involve distinct mechanisms and observable manifestations.

At the same time, we have clarified the scope of the present study. The objective is not to identify or classify specific manipulation types. Instead, this study adopts a screening-oriented risk assessment perspective and focuses on capturing common abnormal patterns observable across heterogeneous manipulation behaviors. In particular, the proposed framework examines whether manipulation risk can be reflected in the joint dynamics of disclosure behavior, sentiment diffusion, and market responses.

This positioning is consistent with practical regulatory settings, where the specific manipulation type is often unknown ex ante and supervisors need to prioritize high-risk observations based on accessible signals. We have revised the Introduction and Section 3.1 accordingly to make the research scope and positioning clearer.

**Amendments in the Manuscript:**

In practice, stock market manipulation involves heterogeneous strategies, including trade-based, information-based, and combined forms. Rather than identifying specific manipulation types, this study focuses on a screening-oriented risk assessment framework that captures common abnormal patterns observable across different manipulation behaviors. **(Page 3, Section 1, lines 53-56)**

In practice, stock market manipulation may operate through different channels. Some strategies rely primarily on trading behavior, such as abnormal price or volume movements, whereas others rely more heavily on information channels, such as misleading disclosure, rumor diffusion, or sentiment amplification. In many cases, trading and information channels may also interact, forming combined manipulation patterns. These strategies differ in their underlying mechanisms and observable manifestations. Rather than focusing on identifying or classifying specific manipulation strategies, this study adopts a screening-oriented perspective and aims to develop a unified risk assessment framework. The objective is to capture common abnormal patterns that are observable across heterogeneous manipulation forms, particularly in the joint dynamics of disclosure behavior, sentiment diffusion, and market responses. Based on this perspective, the present study examines whether manipulation risk can be systematically captured by observable joint deviations across disclosure behavior, sentiment diffusion, and market responses. This study does not aim to identify specific manipulative transactions or actors at the transaction level, but instead focuses on assessing manipulation risk based on observable multi-source signals. **(Page 8, Section 3.1, lines 182-192)**

**Comment 2:**

*Your claimed contribution of “multi-source data fusion” fails to distinguish from that already considered in the literature. Similar approaches have been widely examined in financial analytics and regulatory technology. It’s important that you understand on a more concrete level you need to come for new features to be brought in your framework.*

**Response 2:**

Thank you for this important and constructive comment. We agree that multi-source data fusion has been widely examined in financial analytics and regulatory technology, and that the contribution of this study should not be framed as the general use of multi-source fusion itself.

In the revised manuscript, we have clarified the contribution more concretely. The main contribution lies in adapting multi-source representation to the specific task of stock market manipulation risk assessment in China’s A-share market. Rather than treating “multi-source fusion” as a broad methodological label, the revised manuscript specifies how heterogeneous public-data signals are organized for this task: corporate disclosure behavior, public sentiment diffusion, and market trading responses are jointly represented as observable dimensions of manipulation risk.

We also clarified the concrete feature construction. The framework incorporates announcement-text features, public sentiment features, and market trading features, while the final model further combines these structured variables with BERT–MIL representations of announcement texts. This design allows us to evaluate the incremental value of structured indicators, announcement semantics, and their fusion within the same screening-oriented risk assessment task.

We also revised the literature review to distinguish our framework from prior multi-source applications. Many existing multi-source studies emphasize the general value of heterogeneous data in financial risk analysis or regulatory decision support. In contrast, this study focuses on probabilistic manipulation risk scoring and tiered screening, where the objective is to rank firm–month observations by manipulation risk rather than merely to classify events. These revisions make the contribution more specific and avoid presenting multi-source fusion as a generic novelty.

**Amendments in the Manuscript:**

In the Introduction, we revised the contribution statement to specify the task-specific role of multi-source information:

Compared with prior research, this study offers three main contributions. First, it develops a unified multi-source framework that captures manipulation risk through the joint dynamics of information disclosure, sentiment diffusion, and market trading behavior under a consistent temporal structure. Second, it introduces a BERT–MIL architecture that models multiple announcements within the same period as a set and captures heterogeneous and unevenly distributed information signals that are often diluted in conventional semantic analysis. Third, it formulates stock market manipulation analysis as a probabilistic risk assessment problem that supports risk ranking and tiered screening in regulatory practice, and further enhances interpretability through SHAP (SHapley Additive exPlanations). **(Page 3-4, Section 1, lines 60–67)**

In Section 2.3, we further clarified the methodological gap in existing multi-source fusion studies:

Taken together, existing research supports the value of multi-source information in financial regulation, but important gaps remain in how heterogeneous disclosure, sentiment, and trading signals are jointly represented for manipulation ranking. Building on this literature, the present study integrates announcement texts, public sentiment, and market trading information within a unified risk assessment framework to evaluate their complementary value in probabilistic risk scoring and tiered screening. **(Page 7-8, Section 2.3, lines 165–169)**

In Section 4.1, we clarified how the empirical design evaluates the incremental contribution of different information sources and modeling strategies:

The model design follows a stepwise rationale of “interpretability → nonlinearity → semantic modeling → multi-source fusion.” First, a Logit regression (logistic regression, Logit) using structured features is adopted as the baseline. Second, keeping the inputs unchanged, we replace the baseline with a CatBoost classifier (Categorical Boosting, CatBoost) to examine whether nonlinearities and feature interactions among structured variables improve risk assessment performance. Third, we shift to the text modality by introducing Bidirectional Encoder Representations from Transformers (BERT) representations and combining them with Multiple Instance Learning (MIL) aggregation to assess whether announcement semantics alone can provide effective predictive power. In the final fusion step, the BERT–MIL branch aggregates announcement embeddings into a firm–month textual representation, while the structured-feature branch processes structured announcement indicators, public sentiment variables, and market trading variables in parallel. The two representations are then concatenated and passed to a Multi-Layer Perceptron (MLP) prediction layer, yielding the BERT + MIL + MLP fusion model. **(Page 15, Section 4.1, lines 325–334)**

**Comment 3:**

*Equation (1), used for sentiment scoring, depends on a rather superficial lexicon -based approach. Since NLP with more sophisticated frameworks (for instance transformer-based models) is now the most popular, this seems outdated. A stronger argument or better comparison with a more advanced technique must be included here.*

**Response 3:**

Thank you for this important and helpful comment. We agree that a sentiment score based solely on word counts has limitations in capturing contextual meaning, especially when applied to corporate announcements that often contain standardized and strategically framed language.

In the revised manuscript, we have clarified the role of Eq (1) in the overall modeling framework. The lexicon-based sentiment score is retained as an interpretable structured proxy for announcement tone, rather than as the sole representation of textual information. This design is intended to provide a transparent and reproducible firm-month numerical indicator. It is also complementary to the BERT + MIL text branch in the final fusion model, which directly learns contextual semantic representations from announcement texts. Therefore, the lexicon measure and the BERT + MIL module serve different roles: the former captures an interpretable tone dimension, while the latter captures deeper semantic information that cannot be reduced to word counts.

To directly address the concern regarding the limited contextual capacity of the lexicon-based approach, we added a robustness check using alternative sentiment measures generated by a Chinese transformer sentiment classifier motivated by the FinBERT framework. In this test, the original announcement sentiment variable (ann_sent) and investor forum sentiment variable (sent_mean) are replaced by transformer-generated sentiment measures. The remaining structured variables, sample, labels, announcement texts, and fusion model architecture remain unchanged. The replacement therefore affects only the structured sentiment variables and does not alter the announcement representation module of the fusion model.

The robustness results show that the fusion model retains comparable performance under this alternative sentiment specification. After replacing the two sentiment variables, the model achieves an average AUC of 0.8813, a PR-AUC of 0.6853, Recall@10% of 0.4505, and Recall@20% of 0.6889. These results are close to the main specification reported in Table 3, suggesting that the empirical findings are not dependent on the lexicon-based sentiment construction. We have revised Section 3.2.1 and added Section 4.3.1 accordingly.

**Amendments in the Manuscript:**

In Section 3.2.1, we revised the explanation of the sentiment score in Eq (1) to clarify the role and limitation of the lexicon-based sentiment measure:

Methodologically, sentiment words are identified using a Chinese financial sentiment lexicon constructed with reference to prior Chinese financial sentiment dictionaries [40].

……

In addition, financial sentiment measurement is sensitive to language and domain context, which supports the use of Chinese financial sentiment resources when analyzing Chinese financial texts [41]. In this study, the sentiment score is used as a transparent and reproducible proxy for announcement tone. Given the inherent limits of word count measures in representing contextual meaning, the sensitivity of this measurement choice is further examined in Section 4.3 using alternative sentiment measures constructed with a transformer model. **(Page 10, Section 3.2.1, lines 220–232)**

**Additional references:**

40. Jiang F, Meng L, Tang G. Media Textual Sentiment and Chinese Stock Return Predictability. China Economic Quarterly. 2021;21(4):1323–1344. doi:10.13821/j.cnki.ceq.2021.04.10.

41. Du Z, Huang AG, Wermers R, Wu W. Language and domain specificity: A Chinese financial sentiment dictionary. Review of Finance. 2022;26: 673–719. doi:10.1093/rof/rfab036

We also added Section 4.3.1 to provide a robustness comparison based on an alternative sentiment measurement constructed using a Chinese transformer sentiment classifier:

4.3.1	Alternative Sentiment Measurement

The sentiment score in Eq (1) is constructed from a Chinese financial sentiment lexicon and serves as an interpretable proxy for announcement tone. Measures based on lexicons are transparent and reproducible, but their representation of contextual meaning depends on dictionary coverage and word usage. To assess whether the empirical results are sensitive to this measurement choice, we construct alternative sentiment variables using a Chinese transformer sentiment classifier motivated by the FinBERT framework [47].

The classifier produces sentiment class probabilities, and the alternative sentiment score is defined as P(positive) − P(negative). The resulting sentiment scores are aggregated to the firm–month level and used to replace the original announcement sentiment variable (ann_sent) and investor forum sentiment variable (sent_mean). All remaining structured variables, as well as the sample, labels, announcement texts, and fusion model architecture, remain unchanged. The replacement affects only the structured sentiment variables and does not alter the announcement representation module of the fusion model.

As reported in Table 6, the fusion model retains comparable performance under the alternative sentiment specification. Across five random seeds, the alternative specification achieves an average AUC of 0.8813, a PR-AUC of 0.6853, Recall@10% of 0.4505, and Recall@20% of 0.6889. These results are close to those of the main specification reported in Table 3, indicating that the empirical findings are not materially driven by the specific lexicon-based construction of the sentiment variables. **(Pages 27–28, Section 4.3.1, lines 623–641)**

Table 6 was also added to report the robustness results under the alternative sentiment measurement.

**Table 6. Robustness** **check using alternative sentiment measurement.**

| **Specification** | **Sentiment measurement** | **AUC** | **PR-AUC** | **Recall@10%** | **Recall@20%** |
| --- | --- | --- | --- | --- | --- |
| **Main specification** | Chinese financial sentiment lexicon | 0.8811±0.0063 | 0.6943±0.0158 | 0.4545±0.0294 | 0.6788±0.0166 |
| **Alternative specification** | Chinese transformer sentiment classifier | 0.8813±0.0088 | 0.6853±0.0591 | 0.4505±0.0477 | 0.6889±0.0330 |

**Note:** The main specification is repeated from Table 3 for reference. In the alternative specification, ann_sent and sent_mean are replaced by sentiment measures generated using a Chinese transformer sentiment classifier. Other structured variables, labels, announcement texts, and fusion model architecture remain unchanged.

**Additional reference****:**

47. Huang AH, Wang H, Yang Y. FinBERT: A large language model for extracting information from financial text. Contemporary Accounting Research. 2023;40(2):806–841. doi:10.1111/1911-3846.12832.

**Comment 4:**

*Equation (2), the Fog Index, is presented as a proxy for the manipulation risk in terms of textual complexity. Nevertheless, the argument for readability related to manipulation seems not to have developed effectively. An empirical agreement with readability should be made for such a strong theoretical motivation.*

**Response 4:**

Thank you for this important comment. We agree that the original manuscript did not sufficiently explain why announcement readability is relevant to manipulation risk assessment. We also recognize that the previous wording may have implied a stronger interpretation than the empirical design can support.

In the revised manuscript, we have refined the theoretical rationale for including readability. Rather than presenting the readability index as a direct proxy for manipulation behavior, we now explain it through the disclosure-quality perspective. Specifically, less readable disclosure may increase investors’ information-processing costs and reduce disclosure transparency. In this sense, readability captures how information is communicated to the market, which is relevant to the information environment in which manipulation risk may emerge.

We have also added supporting evidence from prior empirical studies in the Chinese market suggesting that the readability of financial disclosures contains useful information for assessing firm risk, including subsequent stock price crash risk. This revision helps connect the readability measure to the Chinese financial disclosure context, while avoiding an overstatement that textual complexity itself directly indicates manipulation.

In addition, we revised the empirical interpretation of ann_fog in the SHAP analysis. The results show that announcement textual complexity is among the important structured variables in the fusion model, but its effect is conditional rather than mechanical. We therefore interpret ann_fog as one component of the multi-source risk representation framework, whose contribution depends on whether disclosure complexity coincides with abnormal trading activity and sentiment diffusion.

Accordingly, the revised manuscript now defines ann_fog as an observable proxy for the linguistic complexity of corporate announcements within the multi-source risk representation framework. We have also softened stronger expressions that might imply a direct link between complex disclosure and manipulation, and replaced them with a more cautious interpretation focused on textual complexity, information-processing costs, and disclosure transparency.

**Amendments in the Manuscript:**

Third, announcement textual complexity is measured using a Chinese-adapted readability index (ann_fog). Readability is an important dimension of disclosure quality because complex textual expression may increase investors’ information-processing costs and reduce disclosure transparency [42]. Evidence from the Chinese market also suggests that the readability of financial disclosures contains useful information for assessing firm risk, including subsequent stock price crash risk [43]. In this study, ann_fog is used as an observable proxy for the linguistic complexity of corporate announcements within the multi-source risk representation framework. **(Page 10, Section 3.2.1, lines 233-238)**

**Additional references:**

42. Bushee BJ, Gow ID, Taylor DJ. Linguistic complexity in firm disclosures: Obfuscation or information? Journal of Accounting Research. 2018;56(1): 85-121. doi:10.1111/1475-679X.12179

43. Zhang X, Wang B, Liu G. Readability of financial reports and stock price crash risk. Finance Research Letters. 2025;86: 108489. doi:10.1016/j.frl.2025.108489

In the SHAP analysis, we further clarified the empirical role of ann_fog:

Announcement volume (ann_cnt) and announcement textual complexity (ann_fog) rank immediately after CAR, suggesting that formal disclosure behavior provides substantial information in addition to market trading variables. **(Page 26, Section 4.2.2, lines 578-579)**

……

By contrast, announcement textual complexity (ann_fog) and announcement sentiment (ann_sent) show more conditional patterns. Higher values of these disclosure-derived indicators are more often associated with negative SHAP contributions, but their distributions are not strictly one-sided and the effects can vary across observations. This suggests that textual complexity and announcement tone should not be interpreted as mechanical risk amplifiers or risk reducers. Their contributions depend on whether changes in readability or tone coincide with abnormal trading activity and sentiment diffusion. (**Page 26, Section 4.2.2, lines 596-601)**

**Comment 5:**

*In Table we use several variables (eg, announcement sentiment, post count) without any good theoretical basis. The evidence for each of these factors should be established within prior studies (e.g.,) or clearly justified for their relevance to manipulation detection.*

**Response 5:**

Thank you for this important and helpful comment. We agree that the original manuscript did not sufficiently explain the theoretical basis for the variables listed in Table 1. This may have made the logic of the indicator system less explicit.

In the revised manuscript, we have strengthened the theoretical justification of the full variable system in Section 3.1 and revised the variable construction discussion in Section 3.2. Specifically, we clarify that the variables in Table 1 are organized according to three theoretically motivated information dimensions: corporate disclosure behavior, public sentiment diffusion, and market trading responses. These dimensions correspond to the observable channels through which manipulation risk may be reflected in public data.

We also revised the explanation of each feature group to make its financial interpretation and risk relevance clearer. Announcement-text features capture disclosure intensity, managerial tone, and textual complexity; public sentiment features capture investor attention, participation, information exposure, interaction depth, sentiment polarity, and temporal concentration; and market trading features capture return responses, abnormal return accumulation, trading activity, capital involvement, volatility, and market-value changes. In this way, the variables are not treated as isolated predictors, but as observable signals whose relevance is supported by prior studies and by their roles within the multi-source risk representation framework.

This revision provides a clearer theoretical basis for Table 1 and explains why the selected variables are relevant to screening-oriented manipulation risk assessment. It also improves the connection between the theoretical discussion in Section 3.1, the variable construction in Section 3.2, and the indicator system summarized in Table 1.

**Amendments in the Manuscript:**

In Section 3.1, we added a theoretical explanation linking the variable system to three information dimensions:

The theoretical basis of this proposition lies in the complementary roles of three information dimensions. First, formal disclosures issued by listed firms may reflect how firms shape, frame, or communicate information to the market. Disclosure timing, tone, readability, and reporting patterns can influence investor interpretation and may reveal abnormal information presentation associated with elevated risk [6,30,38]. Second, public sentiment generated by market participants captures investor attention, discussion intensity, and the diffusion of expectations in online information environments. Prior studies suggest that investor sentiment, rumors, and managerial responses on interactive platforms can affect market reactions and amplify abnormal information diffusion [21,22,25]. Third, market responses capture the realization of risk signals through returns, volatility, trading intensity, and market value changes. Existing evidence shows that public information manipulation and trading-based manipulation can affect asset prices through feedback mechanisms and abnormal trading responses [20,39]. These three dimensions correspond to the main feature groups used in this study: announcement-text features, public sentiment features, and market trading features. They do not represent isolated hypotheses for individual variables, but jointly define a risk representation framework. From a dynamic perspective, manipulation risk may be reflected when disclosure patterns, sentiment diffusion, and market responses reinforce one another within the same risk process. This multi-source perspective therefore provides the theoretical basis for the variable system constructed in the following section. **(Page 9, Section 3.1, lines 193–206)**

In Section 3.2, we further revised the variable construction subsections to clarify the financial interpretation of each feature group and its relevance to manipulation risk assessment. Specifically, Section 3.2.1 explains the roles of disclosure intensity, announcement tone, and textual complexity in representing corporate disclosure behavior; Section 3.2.2 explains public sentiment variables from the perspectives of investor attention, participation breadth, information exposure, interaction depth, sentiment polarity, and temporal concentration; and Section 3.2.3 explains market trading variables from the perspectives of return response, abnormal return accumulation, trading activity, capital involvement, volatility, and market-value changes. These revisions are reflected in the detailed variable construction discussion in Sections 3.2.1–3.2.3 before the summary table. **(Pages 9–13, Sections 3.2.1–3.2.3, lines 209–306)**

**Comment 6:**

*The overall framework is shown in Figure but the interaction between modules is not well-defined. It is not clear whether textual feature and trading feature are processed separately and fused together or separately throughout the pipeline and subsequently combined in any way.*

**Response 6:**

Thank you for this helpful comment. We agree that the original description of the modeling framework did not make the interaction between the text module and the structured-feature module sufficiently explicit.

In the revised manuscript, we have clarified the fusion process in the text accompanying Figure 1. Specifically, the final fusion model follows a two-branch design. The BERT–MIL branch aggregates announcement embeddings into a firm–month textual representation, while the structured-feature branch processes structured announcement indicators, public sentiment variables, and market trading variables in parallel. These two representations are then concatenated and passed to the MLP prediction layer.

This revision clarifies that textual and structured features are first processed separately to learn branch-specific representations, and are then combined through feature-level fusion before the final risk score is generated.

**Amendments in the Manuscript:**

In the final fusion step, the BERT–MIL branch aggregates announcement embeddings into a firm–month textual representation, while the structured-feature branch processes structured announcement indicators, public sentiment variables, and market trading variables in parallel. The two representations are then concatenated and passed to a Multi-Layer Perceptron (MLP) prediction layer, yielding the BERT + MIL + MLP fusion model. Through this progressive design, the study enables a systematic comparison of the incremental contributions of different information sources and modeling strategies, thereby establishing a coherent empirical pathway. **(Page 15, Section 4.1, lines 331-336)**

**Comment 7:**

*In Section 4.1.1 we employ CatBoost to be a baseline model. Unfortunately we do not add details of hyperparameter tuning or validation strategy. It does not lead us to conclude that model comparisons are fair from the statistical perspective.*

**Response 7:**

Thank you for this important comment. We agree that the original manuscript did not provide enough information on the CatBoost implementation, which could make the baseline comparison appear insufficiently specified.

In the revised manuscript, we have clarified that CatBoost is used as a fixed nonlinear ensemble baseline using structured features rather than as a model selected through separate validation-based tuning. The CatBoost configuration is now explicitly reported in Section 4.1.1: 500 iterations, depth = 4, learning rate = 0.03, Logloss as the loss function, AUC as the evaluation metric, and scale_pos_weight calculated from the training split. This fixed configuration is applied consistently across the repeated random splits. This fixed-baseline design is used to keep the comparison transparent and comparable across model families. The purpose of the comparison is to evaluate the incremental contribution of nonlinear structured-feature modeling, textual semantics, and multi-source fusion under the same data partitions, rather than to maximize each model through separate model-specific hyperparameter searches.

We have also clarified the statistical basis for model comparison. All models are evaluated under the same five stratified random partitions, and the same train/test split is used for all models under each seed. Therefore, the reported comparisons are based on matched experimental conditions rather than different random partitions. In addition, Table 3 reports mean ± standard deviation across the five seeds, and Table 4 further provides paired significance tests at the seed level. These revisions make the comparison more transparent and statistically grounded.

**Amendments in the Manuscript:**

During training and testing, the main model comparison is based on stratified random splits, with 80% of the sample used for training and 20% for testing. To reduce the influence of a particular random partition, all random split experiments are repeated using five random seeds, and model performance is reported as mean ± standard deviation. The same set of random partitions is used across models to ensure comparability. **(Page 15, Section 4.1, lines 337–340)**

In the implementation, missing numerical values are imputed using training-set medians. Logit is estimated with standardized inputs, L2 regularization, and balanced class weights, while CatBoost uses a fixed configuration with 500 iterations, depth = 4, learning rate = 0.03, Logloss, AUC, and a training-split scale_pos_weight. **(Page 17, Section 4.1.1, lines 376–379)**

The same random partitions are used across models, which makes the model comparisons paired at the seed level. Rather than relying only on point estimates, Table 4 further examines whether the performance differences between the final fusion model and each comparison model are statistically supported under this paired design. **(Page 23, Section 4.2.2, lines 517–520)**

**Comment 8:**

*Table 3 reports the model performance but no statistical significance tests are given. As no formal testing is provided (be it paired t-tests or bootstrapping procedures) these improvements are not yet obvious.*

**Response 8:**

Thank you for this important comment. We agree that the original manuscript reported model performance without formal statistical tests, which made it difficult to assess whether the observed improvements were statistically supported under repeated data splits rather than reflecting variation from a particular random partition.

In the revised manuscript, we have added a new Table 4 to report paired significance tests for model performance differences. Specifically, we use the same five repeated random-seed experiments reported in Table 3. For each metric, we first calculate the performance difference between the final BERT + MIL + MLP fusion model and each comparison model under the same random seed. We then conduct seed-level paired t-tests on these paired differences. The reported difference is the average paired difference, and the p-value tests whether the mean paired difference differs from zero.

The revised results provide additional statistical evidence for the model comparisons. The paired tests show that the fusion model’s improvements over Logit and CatBoost are statistically supported across the evaluation metrics. For the comparison with the pure-text BERT + MIL model, we have revised the interpretation more cautiously: the fusion model shows statistically significant improvements in AUC, PR-AUC, and Recall@20%, whereas the improvement in Recall@10% is not statistically significant. We therefore avoid claiming uniform improvement across all screening metrics and instead interpret the added value of multi-source fusion as mainly improving overall ranking quality and broader high-risk coverage.

**Amendments in the Manuscript:**

**Table 3. Comparative Evaluation of Model Performance.**

| **Model** | **Input** | **AUC** | **PR-AUC** | **Recall@10%** | **Recall@20%** |
| --- | --- | --- | --- | --- | --- |
| **Logit** | Numerical features | 0.6984±0.0268 | 0.3549±0.0329 | 0.2646±0.0374 | 0.4202±0.0332 |
| **CatBoost** | Numerical features | 0.7768±0.0389 | 0.5130±0.0562 | 0.3394±0.0382 | 0.4949±0.0647 |
| **BERT + MIL** | Announcement text | 0.8296±0.0286 | 0.6464±0.0464 | 0.4384±0.0444 | 0.6323±0.0402 |
| **BERT + MIL + MLP** | Announcement text + numerical features | 0.8811±0.0063 | 0.6943±0.0158 | 0.4545±0.0294 | 0.6788±0.0166 |

**Note:** Values are reported as mean ± SD across five random seeds. The same random partitions are used across models.

**Table 4. Paired significance tests based on repeated random seeds.**

| **Comparison** | **AUC diff.** | **AUC p-value** | **PR-AUC diff.** | **PR-AUC p-value** | **Recall@10% diff.** | **Recall@10% p-value** | **Recall@20% diff.** | **Recall@20% p-value** |
| --- | --- | --- | --- | --- | --- | --- | --- | --- |
| **Fusion vs Logit** | 0.1827 | <0.001 | 0.3394 | <0.001 | 0.1899 | 0.0026 | 0.2586 | <0.001 |
| **Fusion vs CatBoost** | 0.1043 | 0.0028 | 0.1813 | 0.0018 | 0.1152 | 0.0079 | 0.1838 | 0.0023 |
| **Fusion vs BERT+MIL** | 0.0515 | 0.0091 | 0.0478 | 0.0388 | 0.0162 | 0.4382 | 0.0465 | 0.0303 |

**Note:** This table reports seed-level paired t-tests based on the same five repeated random-seed experiments used in Table 3. For each metric, the difference is calculated as the performance of the final fusion model minus that of the comparison model under the same seed. The reported diff. is the average paired difference, and the p-value tests whether the mean paired difference differs from zero.

In Section 4.2.2, we revised the interpretation of the model comparison results as follows:

To reduce dependence on a particular random partition, all random split experiments are repeated using five random seeds, and Table 3 reports model performance as mean ± standard deviation. The same random partitions are used across models, which makes the model comparisons paired at the seed level. Rather than relying only on point estimates, Table 4 further examines whether the performance differences between the final fusion model and each comparison model are statistically supported under this paired design. The relatively small standard deviations of the fusion model, particularly for AUC, PR-AUC, and Recall@20%, indicate that its performance is not highly sensitive to a particular random split. **(Page 23, Section 4.2.2, lines 516–521)**

……

Building on this foundation, the final BERT + MIL + MLP model combines the announcement-text representation with the 15 structured numerical indicators and achieves the highest average performance across all four metrics. The paired tests provide statistical support for the improvements over the two structured-feature models, suggesting that these gains are unlikely to be driven solely by random split variation. Relative to the pure-text BERT + MIL model, the evidence is more nuanced. The fusion model shows statistically significant improvements in AUC, PR-AUC, and Recall@20%, whereas the improvement in Recall@10% is not statistically significant. This pattern suggests that multi-source fusion mainly improves overall ranking quality and broader high-risk coverage, while its additional contribution is less pronounced within the most restrictive Top 10% screening threshold. **(Page 24, Section 4.2.2, lines 536–543)**

**Comment 9:**

*Fig. 4 (radar chart) does not display nicely labeled axes and numerical scales; this drastically reduces interpretation. The figure should have value and to have more quantitative meaning.*

**Response 9:**

Thank you for this helpful comment. We agree that the original Figure 4 did not provide sufficiently clear numerical references, which limited its interpretability and quantitative meaning.

In the revised manuscript, we have redesigned Figure 4 to improve its readability and quantitative interpretation. Specifically, we added clearly labeled radial numerical scales, clarified that all variables are expressed as standardized feature means on a common z-score scale, and included a side table reporting selected standardized mean values for representative variables. We also refined the axis labels and organized the variables into announcement, public sentiment, and market trading dimensions to make the multidimensional feature profile easier to interpret.

We have also revised the accompanying text to clarify the interpretation of Figure 4. Figure 3 quantifies variable-level group differences using Cohen’s d and 95% confidence intervals, whereas Figure 4 presents the standardized feature profiles of violating and non-violating observations across multiple information dimensions. These revisions strengthen the quantitative meaning of Figure 4 and clarify its complementary role in the descriptive analysis.

**Amendments in the Manuscript:**

**Fig 4. Radar profile of standardized feature means by risk state.**

Figure 4 presents the standardized feature profiles of violating and non-violating observations within the multidimensional feature space. All variables are standardized on a common z-score scale, where zero denotes the sample average. Positive values indicate above-average feature levels, whereas negative values indicate below-average levels. The overall profile indicates that violating observations exhibit an outward shift along market trading dimensions, particularly around CAR, MCCR, and Vol. This pattern is consistent with the view that market manipulation is frequently accompanied by abnormal price and trading responses. By contrast, non-violating observations display a more concentrated distribution across most indicators, reflecting comparatively stable trading conditions. Differences in announcement tone, textual complexity, and public sentiment are less pronounced in magnitude, but they remain visible as part of the broader multidimensional profile. Together with the effect-size evidence in Figure 3, this pattern suggests that manipulation risk is better represented by the joint configuration of market response, disclosure characteristics, and sentiment dynamics than by a single class of variables alone. **(Page 22, Section 4.2.2, lines 497-506)**

**Comment 10:**

*The 1:5 matching ratio used in sample construction isn't justified. Explain on class imbalance and model performance reasons.*

**Response 10:**

Thank you for this helpful comment. We agree that the original manuscript did not sufficiently justify the choice of the 1:5 matching ratio.

In the revised manuscript, we have clarified that the 1:5 ratio was adopted as a practical compromise between sample comparability and the requirements of supervised model training. Since manipulation-related observations are relatively rare, using too few matched controls may provide an insufficient representation of comparable non-violating firm–month observations and limit the information available for model learning. At the same time, including an excessively large number of controls may enlarge the majority class too much and make model training and evaluation overly dominated by the majority class. Therefore, the 1:5 ratio was used to retain sufficient comparable control observations for supervised model training while preventing the matched control group from becoming excessively large.

We have also expanded the description of the matching procedure and added covariate balance diagnostics. Specifically, each violating firm-month observation is matched with five non-violating firm-month observations using nearest-neighbor matching within the same month and industry, with matching distance calculated based on log market capitalization. The balance diagnostics reported in Panel B of Table 2 show that the absolute standardized mean differences are 0.0320 for log market capitalization and 0.0206 for raw market capitalization, both below the conventional 0.1 threshold. These results indicate satisfactory balance in the observed size-related matching covariates.

**Amendments in the Manuscript:**

To ensure sample comparability, each violating firm–month observation is matched with five non-violating firm–month observations using nearest-neighbor matching within the same month and industry. Industry categories follow the CSRC classification standards, and nearest-neighbor distance is calculated based on log market capitalization. The 1:5 ratio is adopted to retain sufficient comparable control observations while keeping class imbalance at a manageable level for the supervised risk assessment task. Matching quality is assessed through covariate balance diagnostics at the matched-sample construction stage. Since month and industry are exactly matched by construction, the diagnostics focus on firm size. As shown in Panel B of Table 2, the absolute standardized mean differences are 0.0320 for log market capitalization and 0.0206 for raw market capitalization, both below the conventional 0.1 threshold, indicating good balance on the observed size-related matching covariates. **(Pages 19-20, Section 4.2.1, lines 434-442)**

We also added Panel B to Table 2 to report covariate balance diagnostics for the matched sample:

**Panel B. Covariate balance diagnostics for matched-sample construction.**

| **Covariate** | **Violating mean** | **Matched control mean** | **SMD** | **p-value** |
| --- | --- | --- | --- | --- |
| **Log market capitalization** | 14.8456 | 14.8750 | -0.0320 | 0.4408 |
| **Market capitalization** | 4,646,140 | 4,783,079 | -0.0206 | 0.6124 |

**Note:** Panel B reports covariate balance diagnostics at the matched-sample construction stage. Matching is performed within the same month and industry, and the nearest-neighbor distance is calculated based on log market capitalization. Since month and industry are exactly matched by construction, the diagnostics focus on size-related covariates. SMD denotes standardized mean difference. The p-values test mean differences between violating and matched control observations.

**Comment 11:**

*The use of regulatory penalties as labels can create a degree of look-ahead bias, even to the extent violations are identified ex post. This issue should be acknowledged and addressed.*

**Response 11:**

Thank you for this important comment. We agree that labels based on regulatory penalty cases are retrospective by nature and that this issue should be explicitly clarified in the empirical design.

In the revised manuscript, we have clarified the role of regulatory sanction records in the label construction process. The sanction records are used ex post to define supervised learning labels based on the violation intervals disclosed in the penalty documents. They are not used as explanatory variables in model training. No penalty announcement date, enforcement outcome, investigation information, or post-period regulatory information is included as model input. In other words, the enforcement outcome is used to identify historically sanctioned violation months, but later enforcement information is not included as predictive information.

We have also clarified the interpretation of the modeling task. The model is not intended to provide real-time or transaction-level evidence of manipulation. Rather, it is designed as a screening-oriented risk assessment model that learns observable public-data patterns associated with retrospectively identified violation months and produces periodic manipulation-risk scores. This interpretation is consistent with the firm–month structure of the dataset and with the use of publicly observable disclosure, sentiment, and market trading signals.

This revision acknowledges the retrospective nature of the regulatory labels while clarifying that the empirical design does not use later enforcement outcomes as input features. It also helps readers interpret the model output as a probabilistic risk score for screening and prioritization, rather than as direct evidence of manipulation behavior.

**Amendments in the Manuscript:**

In Section 4.2.1, we clarified the retrospective nature of the labeling strategy and the role of regulatory sanction records:

The sanction records are used ex post to define supervised learning labels and are not used as explanatory variables in model training. The model is therefore interpreted as learning observable public-data patterns associated with retrospectively identified violation months and producing periodic manipulation-risk scores. **(Page 19, Section 4.2.1, lines 431-433)**

This clarification is also consistent with the revised description of the study’s scope in Section 3.1:

This study does not aim to identify specific manipulative transactions or actors at the transaction level, but instead focuses on assessing manipulation risk based on observable multi-source signals. **(Page 8, Section 3.1, lines 191-192)**

The model-output interpretation is further clarified in Section 4.1:

Across all models, the output is interpreted as a probabilistic risk score reflecting manipulation-related risk. This interpretation is consistent with the screening-oriented objective of the study, where the model is used to prioritize high-risk observations. **(Page 15, Section 4.1, lines 341–343)**

In the Conclusion, we further acknowledged the limitation of labels constructed from ex post regulatory penalty documents:

Despite these findings, the current model has several limitations. First, constrained by public data availability and labels constructed from ex post regulatory penalty documents, the model does not incorporate transaction-level order or trade information and therefore primarily captures statistical associations rather than directly identifying specific manipulative actions. Accordingly, the model output should be interpreted as a probabilistic risk score for candidate review and prioritization, and should be considered together with regulatory judgment and contextual verification. Legitimate information shocks, market-wide events, or sector-level fluctuations may also generate abnormal returns, sentiment bursts, and trading fluctuations, thereby increasing false positive risk in screening applications. **(Page 31, Section 5, lines 704-710)**

**Comment 12:**

*According to Section 4.1.3, we cannot precisely characterize the architecture of the fusion model. The MLP structure (number of layers, number of neurons, activation functions) must be defined in a compact way in order for reproducibility to be ensured.*

**Response 12:**

Thank you for this important comment. We agree that the original description of the fusion model was too conceptual and did not provide sufficient architectural details for reproducibility.

In the revised manuscript, we have added a compact implementation description in Section 4.1.3. Specifically, we now clarify that the BERT–MIL branch produces a 768-dimensional text representation, while the structured numerical branch maps the 15 numerical variables into a 64-dimensional representation. These two representations are concatenated and passed through a 128-dimensional hidden layer before the final sigmoid output. We also specify the main activation function and training setting: the numerical branch uses ReLU activation, the fusion prediction layer uses a 128-dimensional hidden representation before the final output, and the model is trained using AdamW with a learning rate of 2e-3.

We also revised the previous general description of the MLP to match the implemented model more accurately. This revision makes the architecture of the final BERT + MIL + MLP fusion model more transparent and reproducible.

**Amendments in the Manuscript:**

In the empirical implementation, the 15 structured variables are projected into a 64-dimensional ReLU-activated representation and concatenated with the 768-dimensional BERT–MIL text representation. The fused vector is passed through a 128-dimensional ReLU-activated hidden layer, followed by a sigmoid output layer, and the model is trained for 20 epochs using AdamW with a learning rate of 2e-3. **(Page 19, Section 4.1.3, lines 417-420)**

**Comment 13:**

*SHAP analysis (Figure 5) finds CAR to be the most important variable. CAR is a factor that directly receives price changes so the model is, in reality, generating price anomalies and not only manipulating them so much. We say that this could potentially be a problem.*

**Response 13:**

Thank you for this important and thoughtful comment. We agree that the high SHAP importance of cumulative abnormal returns (CAR) raises a legitimate concern. CAR directly reflects realized abnormal price movements, and therefore it should not be interpreted as transaction-level evidence of manipulation or as a stand-alone leading indicator. We also agree that, if the model relied only on CAR, the framework would mainly capture price anomalies rather than provide a multi-source assessment of manipulation risk.

In the revised manuscript, we have addressed this issue in two ways. First, we revised the interpretation of the SHAP results to clarify the role of CAR. CAR is now described as a price response variable that captures the market-level manifestation of risk, rather than as direct evidence of manipulative behavior. At the same time, we emphasize that the explanatory structure of the model is not confined to CAR: announcement volume, announcement textual complexity, and sentiment-related variables also rank among the important features, indicating that disclosure behavior and investor attention provide additional information beyond market trading variables.

Second, to directly examine whether the model is driven primarily by CAR, we added a feature ablation analysis in Section 4.3.2 by removing CAR from the structured numerical feature set. The sample, labels, announcement texts, remaining structured variables, and fusion model architecture are kept unchanged. The results show that removing CAR leads to a decline in performance, which indicates that CAR contains substantial information for manipulation risk assessment. Specifically, the average AUC decreases from 0.8811 to 0.8370, PR-AUC decreases from 0.6943 to 0.5743, Recall@10% decreases from 0.4545 to 0.3818, and Recall@20% decreases from 0.6788 to 0.6121. These results indicate that CAR is an important market response signal.

However, the CAR exclusion specification still retains meaningful risk ranking capacity, with an AUC of 0.8370 and a Recall@20% of 0.6121. This suggests that announcement semantics, disclosure characteristics, sentiment variables, and the remaining trading indicators continue to provide useful screening information after CAR is removed. Therefore, the revised results support a more cautious interpretation: CAR improves model performance, but the risk assessment output is not reducible to accumulated abnormal returns alone.

Accordingly, we have revised the SHAP discussion and added Section 4.3.2 and Table 7 to report the CAR ablation analysis.

**Amendments in the Manuscript:**

In the SHAP interpretation section, we revised the discussion of CAR to avoid interpreting it as direct evidence of manipulation:

As shown in Figure 5, the CAR variable ranks first in the global importance results, indicating that abnormal return deviations provide the strongest structured signal for the model’s risk scoring. This finding is consistent with the role of price responses in market manipulation cases, where abnormal returns often represent an observable market-level manifestation of risk. Announcement volume (ann_cnt) and announcement textual complexity (ann_fog) rank immediately after CAR, suggesting that formal disclosure behavior provides substantial information in addition to market trading variables. Sentiment-related indicators, including posting volume (post_cnt) and the daily variability of posting activity (post_cnt_std_day), also appear among the important features, suggesting that the model assigns greater importance to the intensity and temporal clustering of investor attention than to passive information exposure. These results indicate that the fusion model captures not only abnormal price movements, but also variation in corporate disclosure behavior and the concentration of investor attention. **(Page 26, Section 4.2.2, lines 575–583)**

We also added Section 4.3.2 to examine the contribution of CAR through a feature ablation analysis:

4.3.2	Feature Ablation of CAR

The SHAP analysis identifies cumulative abnormal returns (CAR) as the most influential structured trading feature. To assess the incremental role of this price response variable, we conduct a feature ablation by removing CAR from the structured numerical feature set. The sample, labels, announcement texts, remaining structured variables, and model architecture are kept unchanged.

Table 7 reports the results of the CAR ablation analysis. After CAR is removed, model performance declines across all evaluation metrics. The average AUC decreases from 0.8811 to 0.8370, PR-AUC decreases from 0.6943 to 0.5743, Recall@10% decreases from 0.4545 to 0.3818, and Recall@20% decreases from 0.6788 to 0.6121. These results indicate that CAR provides substantial information for manipulation risk assessment and constitutes an important component of the market trading feature group.

Despite this decline, the CAR exclusion specification continues to show risk ranking capacity, with an AUC of 0.8370 and a Recall@20% of 0.6121. This suggests that announcement semantics, disclosure characteristics, sentiment variables, and the remaining trading indicators still contain useful screening information after CAR is removed. Therefore, the full model should be interpreted as a multi-source risk assessment framework in which abnormal return information improves model performance, while textual, sentiment, and other trading signals also contribute to the final risk assessment output. **(Pages 28-29, Section 4.3.2, lines 643–657)**

We further added Table 7 to report the feature ablation results excluding CAR.

**Table 7. Feature ablation analysis excluding CAR.**

| **Specification** | **Feature setting** | **AUC** | **PR-AUC** | **Recall@10%** | **Recall@20%** |
| --- | --- | --- | --- | --- | --- |
| **Main specification** | Full feature set | 0.8811±0.0063 | 0.6943±0.0158 | 0.4545±0.0294 | 0.6788±0.0166 |
| **CAR excluded specification** | CAR removed from structured numerical features | 0.8370±0.0236 | 0.5743±0.0591 | 0.3818±0.0522 | 0.6121±0.0382 |

**Note:** The main specification is repeated from Table 3 for reference. In the CAR exclusion specification, cumulative abnormal returns are removed from the structured numerical feature set. The sample, labels, announcement texts, remaining structured variables, and fusion model architecture remain unchanged.

**Comment 14:**

*The evaluation metric in Table 3 does not measure variability (e.g. standard deviation or confidence intervals). Without them, the stability of the results are meaningless.*

**Response 14:**

Thank you for this important comment. We agree that reporting only point estimates in the original version was insufficient for assessing the stability of model performance across repeated experiments.

In the revised manuscript, we have updated Table 3 to report all evaluation metrics as mean ± standard deviation across five repeated random-seed experiments. The same random partitions are used across models to ensure comparability. This revision allows readers to evaluate both the average predictive performance and the variability of each model across repeated splits.

We have also revised the discussion in Section 4.2.2 to explicitly interpret the standard deviations. In particular, we note that the final BERT + MIL + MLP fusion model shows relatively small standard deviations, especially for AUC, PR-AUC, and Recall@20%, suggesting that its performance is less sensitive to a particular random split. These revisions provide a clearer basis for assessing the stability of the reported results.

**Amendments in the Manuscript:**

**Table 3. Comparative Evaluation of Model Performance.**

| **Model** | **Input** | **AUC** | **PR-AUC** | **Recall@10%** | **Recall@20%** |
| --- | --- | --- | --- | --- | --- |
| **Logit** | Numerical features | 0.6984±0.0268 | 0.3549±0.0329 | 0.2646±0.0374 | 0.4202±0.0332 |
| **CatBoost** | Numerical features | 0.7768±0.0389 | 0.5130±0.0562 | 0.3394±0.0382 | 0.4949±0.0647 |
| **BERT + MIL** | Announcement text | 0.8296±0.0286 | 0.6464±0.0464 | 0.4384±0.0444 | 0.6323±0.0402 |
| **BERT + MIL + MLP** | Announcement text + numerical features | 0.8811±0.0063 | 0.6943±0.0158 | 0.4545±0.0294 | 0.6788±0.0166 |

**Note:** Values are reported as mean ± SD across five random seeds. The same random partitions are used across models.

To reduce dependence on a particular random partition, all random split experiments are repeated using five random seeds, and Table 3 reports model performance as mean ± standard deviation. The same random partitions are used across models, which makes the model comparisons paired at the seed level. Rather than relying only on point estimates, Table 4 further examines whether the performance differences between the final fusion model and each comparison model are statistically supported under this paired design. The relatively small standard deviations of the fusion model, particularly for AUC, PR-AUC, and Recall@20%, indicate that its performance is not highly sensitive to a particular random split. **(Page 23, Section 4.2.2, lines 516–521)**

**Comment 15:**

*The discussion section is short and relatively generalizing and not sufficient in detail of the literature. You need to be more critical in describing and assessing your observations with reference to other studies and to better understand the benefits and dangers in theory and regulatory practice.*

**Response 15:**

Thank you for this important comment. We appreciate the reviewer’s concern that the manuscript should more critically relate the empirical findings to prior studies and should more clearly address both the benefits and the potential risks of the proposed framework in theory and regulatory practice.

In the revised manuscript, we have strengthened the discussion in two ways. First, we revised the literature review and theoretical sections to position the study more clearly relative to prior work. The revised manuscript clarifies that existing manipulation studies are often centered on trading anomalies or binary detection, whereas this study focuses on firm–month manipulation risk assessment and risk ranking based on observable multi-source signals. We also explain how announcement semantics, public sentiment, and market trading variables provide complementary information rather than functioning as isolated predictors.

Second, we expanded the concluding discussion to clarify both the benefits and the risks of the proposed framework. The theoretical value of the study is now framed as extending manipulation analysis from a single-source identification perspective to a multi-source risk representation perspective. At the same time, we added a more explicit discussion of the limitations and potential risks in regulatory practice. In particular, we clarify that the model captures statistical associations rather than causal mechanisms, and that high risk scores should not be interpreted as direct evidence of manipulation. Legitimate information shocks, market-wide events, or sector-level fluctuations may also generate abnormal returns, sentiment bursts, and trading fluctuations, thereby increasing false positive risk in screening applications.

These revisions make the discussion more critical and balanced. The revised manuscript presents the model as a screening-oriented risk prioritization tool that can support regulatory review under resource constraints, while clearly acknowledging the need for contextual verification and regulatory judgment.

**Amendments in the Manuscript:**

We revised Sections 2.1–2.3 to provide a more critical discussion of prior manipulation detection, sentiment analysis, and multi-source financial regulation studies. The revised literature review now distinguishes trading-anomaly and binary-detection studies from the screening-oriented risk assessment task of this study, and clarifies the complementary roles of disclosure, public sentiment, and trading signals. **(Pages 4–8, Sections 2.1–2.3, lines 71–169)**

We also expanded the concluding discussion to clarify the theoretical contribution, regulatory value, and practical limitations of the proposed framework:

From a theoretical standpoint, this study extends stock market manipulation analysis from a single-source identification perspective to a multi-source risk representation perspective. The empirical evidence shows that announcement semantics contain risk-relevant information beyond manually constructed numerical indicators, while structured sentiment and trading variables provide complementary signals about attention diffusion and market response. From a practical perspective, the value of the framework lies in converting heterogeneous public signals into a ranked list of firm–month observations for further review. The screening utility analysis further links the risk scores to capacity-based review thresholds, showing how the Top 5%, Top 10%, and Top 20% candidate review sets differ in review burden, risk coverage, and false positive burden. These findings provide an operational basis for risk-based regulatory review, in which limited supervisory resources can be allocated to firm–month observations with higher relative risk scores under predefined review capacity constraints. **(Page 31, Section 5, lines 695–703)**

……

Accordingly, the model output should be interpreted as a probabilistic risk score for candidate review and prioritization, and should be considered together with regulatory judgment and contextual verification. Legitimate information shocks, market-wide events, or sector-level fluctuations may also generate abnormal returns, sentiment bursts, and trading fluctuations, thereby increasing false positive risk in screening applications. **(Page 31, Section 5, lines 707–710)**

**Comment 16:**

*The manuscript is lacking greater clarity, especially on practical applications. In particular: with regulation, market participants can see a role of your model in real monitoring systems? This idea is currently still unappreciated.*

**Response 16:**

Thank you for this practical comment. We agree that the original manuscript did not explain clearly enough how the proposed model could be used in a real regulatory monitoring system.

In the revised manuscript, we have clarified that the model is not intended to replace regulatory investigation or serve as an automatic enforcement decision tool. Instead, its practical role is to support front-end risk screening and review prioritization. In a regulatory monitoring workflow, publicly observable firm–month information, including corporate disclosures, retail-facing public opinion signals, and market trading indicators, can be integrated to generate a probabilistic manipulation risk score for each firm–month observation. These scores can then be used to rank firms and construct candidate review lists under limited supervisory resources. In such a workflow, the model does not make enforcement decisions; it updates a ranked list of candidate firm–month observations for further regulatory review.

To make this practical role more concrete, we added a screening utility analysis based on capacity-based review thresholds. The Top 5%, Top 10%, and Top 20% highest-risk observations are treated as candidate review sets, and we report the corresponding review burden, true positives, false positives, precision, recall, and false discovery rate. This analysis shows how the model can support tiered screening by quantifying the trade-off between risk coverage and false positive burden. We also clarified the boundary of practical application. High risk scores should be interpreted as signals for further review rather than direct evidence of manipulation. In practice, they should be combined with regulatory judgment, contextual verification, market-wide information, sector-level events, and firm-specific news before any supervisory conclusion is made. These revisions make the role of the model in regulatory monitoring systems clearer and more operational.

**Amendments in the Manuscript:**

In Section 4.1, we clarified the interpretation of the model output in a regulatory screening setting:

Across all models, the output is interpreted as a probabilistic risk score reflecting manipulation-related risk. This interpretation is consistent with the screening-oriented objective of the study, where the model is used to prioritize high-risk observations. **(Page 15, Section 4.1, lines 341–343)**

In Section 4.2.2, we added a screening utility analysis under capacity-based review thresholds. Table 5 reports the number of reviewed observations, true positives, false positives, precision, recall, and false discovery rate under Top 5%, Top 10%, and Top 20% review thresholds. This analysis provides a quantitative assessment of the trade-off between review burden, risk coverage, and false positive burden. **(Pages 24-25, Section 4.2.2, lines 549–563)**

To complement the aggregate performance metrics, we further examine the screening utility of the final fusion model under capacity-based review thresholds. Test observations are ranked by risk scores assigned by the model, and the top 5%, 10%, and 20% are treated as candidate review sets. This analysis reports the number of firm–month observations selected for review, the number of true and false positives, precision, recall, and the false discovery rate. In this way, the model output is evaluated in terms of the practical trade-off among review burden, risk coverage, and false positive burden. **(Pages 24-25, Section 4.2.2, lines 549–553)**

**Table 5. Screening utility under capacity-based review thresholds**

| **Screening threshold** | **Reviewed observations** | **True positives** | **False positives** | **Precision** | **Recall** | **False discovery rate** |
| --- | --- | --- | --- | --- | --- | --- |
| **Top 5%** | 29 | 26.4 ± 0.9 | 2.6 ± 0.9 | 0.9103 ± 0.0308 | 0.2667 ± 0.0090 | 0.0897 ± 0.0308 |
| **Top 10%** | 59 | 45.0 ± 2.9 | 14.0 ± 2.9 | 0.7627 ± 0.0494 | 0.4545 ± 0.0294 | 0.2373 ± 0.0494 |
| **Top 20%** | 118 | 67.2 ± 1.6 | 50.8 ± 1.6 | 0.5695 ± 0.0139 | 0.6788 ± 0.0166 | 0.4305 ± 0.0139 |

**Note:** Except for reviewed observations, values are mean ± SD across five random seeds. Reviewed observations are firm–month observations. False discovery rate = false positives / reviewed observations.

Table 5 shows that the screening threshold materially affects the balance between risk coverage and false positive burden. A stricter Top 5% threshold yields high precision and a low false discovery rate, whereas expanding the review set to Top 20% substantially improves recall but also increases the false discovery rate. The Top 10% threshold provides an intermediate setting, capturing nearly half of the true risk observations while keeping the false discovery rate below 25%. These results provide a quantitative basis for review prioritization under limited regulatory resources using the risk scores assigned by the model. **(Page 25, Section 4.2.2, lines 558–563)**

Response to Reviewer 2:

**Comment 1:**

*The subject of the study is original and valuable. However, it can be clearly stated that the study suffers from a significant lack of literature review. A research of this level should have incorporated far more sources and provided a much more in-depth discussion. In fact, due to the scarcity of the literature, the discussion section has been entirely overlooked and remains unwritten. The conclusion section is too brief, and the need for a proper 'implications' section is evident. Frankly speaking, these deficiencies are sufficient for the manuscript to receive a major revision. I believe the study must be revised so that a more thorough evaluation can be conducted in the second round.*

**Response 1:**

We sincerely appreciate the reviewer’s frank and constructive assessment of the manuscript and the recognition of the potential value of the research topic. We also fully understand the serious concern that the original version did not provide a sufficiently developed literature review, discussion, and implications.

We treated this comment as a fundamental issue concerning the overall positioning and presentation of the manuscript, rather than as a minor textual revision. In response, we made substantial revisions throughout the manuscript. The revised version strengthens the literature review, clarifies the theoretical positioning of the study, improves the justification of the multi-source variable system, makes the model design and empirical evaluation more transparent, and expands the discussion of regulatory applicability, false positive risk, causal limitations, and generalizability.

A central aim of the revision was to make the contribution more precise. The manuscript now positions the study as a screening-oriented firm–month manipulation risk assessment framework based on publicly observable disclosure, sentiment, and trading signals. Accordingly, the model output is interpreted as a probabilistic risk score for risk ranking and candidate review, rather than as transaction-level evidence of manipulation.

Because the reviewer’s overall assessment is further developed in the detailed comments below, we respond to each issue individually and indicate the corresponding revisions in the manuscript. We hope that the revised manuscript more clearly demonstrates the theoretical motivation, empirical rigor, and practical boundaries of the proposed framework.

**Comment 2:**

*Manipulation is stated as the subject of the study, but the type of manipulation is not specified: what specific violations were committed, and what manipulation strategies are used? For example, a pump and dump strategy or a strategy of sequentially accumulating positions over several months are significantly different from one another and, therefore, will exhibit different indicators of changes in trading performance. A single model cannot effectively detect all types of manipulation simultaneously.*

**Response 2:**

Thank you for this important and insightful comment. We fully agree that different manipulation strategies, such as pump-and-dump behavior and gradual position accumulation, may exhibit heterogeneous behavioral patterns and distinct trading dynamics.

In the revised manuscript, we have clarified that the objective of this study is not to identify or classify specific manipulation strategies. We agree with the reviewer that a single firm–month model should not be interpreted as a mechanism-specific detector for all manipulation strategies. Instead, this study focuses on developing a unified risk assessment framework that captures common observable risk signals that may appear across heterogeneous manipulation behaviors, as reflected in regulatory sanction cases involving different forms of manipulation. Although different manipulation mechanisms may vary at the transaction level, they may still generate observable joint deviations across disclosure behavior, sentiment dynamics, and market responses.

This design is motivated by practical regulatory scenarios, where manipulation types are typically unknown ex ante, and front-end screening requires a generalizable mechanism for prioritizing high-risk observations based on accessible signals rather than predefined behavioral categories. We also explicitly acknowledge that this approach abstracts from the heterogeneity of underlying manipulation mechanisms and therefore focuses on risk representation rather than mechanism-specific identification.

We have revised the manuscript accordingly in Section 1 and Section 3.1 to clarify the scope of the study, explicitly acknowledge the heterogeneity of manipulation strategies, and position the proposed framework as a screening-oriented approach.

**Amendments in the Manuscript:**

In practice, stock market manipulation involves heterogeneous strategies, including trade-based, information-based, and combined forms. Rather than identifying specific manipulation types, this study focuses on a screening-oriented risk assessment framework that captures common abnormal patterns observable across different manipulation behaviors. **(Page 3, Section 1, lines 53-56)**

In practice, stock market manipulation may operate through different channels. Some strategies rely primarily on trading behavior, such as abnormal price or volume movements, whereas others rely more heavily on information channels, such as misleading disclosure, rumor diffusion, or sentiment amplification. In many cases, trading and information channels may also interact, forming combined manipulation patterns. These strategies differ in their underlying mechanisms and observable manifestations. Rather than focusing on identifying or classifying specific manipulation strategies, this study adopts a screening-oriented perspective and aims to develop a unified risk assessment framework. The objective is to capture common abnormal patterns that are observable across heterogeneous manipulation forms, particularly in the joint dynamics of disclosure behavior, sentiment diffusion, and market responses. Based on this perspective, the present study examines whether manipulation risk can be systematically captured by observable joint deviations across disclosure behavior, sentiment diffusion, and market responses. This study does not aim to identify specific manipulative transactions or actors at the transaction level, but instead focuses on assessing manipulation risk based on observable multi-source signals. **(Page 8, Section 3.1, lines 182-192)**

**Comment 3:**

*The most important and fundamental point if we're going to discuss manipulation is that stock price manipulation involves very specific transactions and actions by an individual or group of individuals. The current methodology doesn't address this at all, and this important nuance is overlooked. The authors examine stock prices, not individual transactions, which are what constitute manipulative trading.*

**Response 3:**

Thank you for this important and fundamental comment. We fully agree that stock price manipulation, in its strict definition, involves specific trading actions carried out by individuals or groups at the transaction level.

In the revised manuscript, we have clarified that the objective of this study is not to directly detect manipulative transactions or identify specific actors. Instead, the study adopts a screening-oriented perspective and focuses on assessing manipulation risk based on observable multi-source public signals, including disclosure behavior, sentiment dynamics, and market responses. This distinction reflects two complementary research perspectives. Transaction-level studies aim to identify specific manipulative trading behaviors using detailed order or trade data, whereas the present study focuses on whether publicly observable firm–month signals can support risk ranking and candidate review.

We also acknowledge this as a limitation of the current empirical design. Because transaction-level order and trade information is not incorporated, the model should not be interpreted as providing direct evidence of manipulation behavior. Its output is a probabilistic risk score for screening and prioritization, which should be considered together with regulatory judgment and contextual verification.

We have revised the manuscript accordingly in Section 3.1 and the Conclusion to clarify the scope and limitations of the proposed framework.

**Amendments in the Manuscript:**

This study does not aim to identify specific manipulative transactions or actors at the transaction level, but instead focuses on assessing manipulation risk based on observable multi-source signals. **(Page 8, Section 3.1, lines 191-192)**

In the Conclusion, we further clarified this limitation:

First, constrained by public data availability and labels constructed from ex post regulatory penalty documents, the model does not incorporate transaction-level order or trade information and therefore primarily captures statistical associations rather than directly identifying specific manipulative actions. Accordingly, the model output should be interpreted as a probabilistic risk score for candidate review and prioritization, and should be considered together with regulatory judgment and contextual verification. **(Page 31, Section 5, lines 704–708)**

**Comment 4:**

*To reliably identify manipulation, it's necessary to have access to the order and trade register, research them, and learn to identify specific suspicious transactions performed by specific individuals. If this isn't available in the work, then one can say something like, "With a probability of XX%, during a certain observation period, there were suspicious changes in the exchange characteristics of a specific asset, accompanied by increased sentiment on social media and other factors."*

**Response 4:**

Thank you for this valuable comment. We fully agree that transaction-level order and trade data are essential for directly identifying specific manipulative actions.

In this study, however, the objective is to assess manipulation risk based on observable multi-source signals, including disclosure behavior, sentiment dynamics, and market responses. The model output is therefore interpreted as a probabilistic risk score reflecting manipulation-related risk. Specifically, it indicates the extent to which a firm–month observation exhibits suspicious public-signal patterns associated with elevated manipulation risk. This perspective is consistent with a screening-oriented approach, where the model is used to prioritize high-risk observations for further investigation.

We have clarified this interpretation in Section 4.1 when describing the model output, and further discussed its implications in Section 5.

**Amendments in the Manuscript:**

Across all models, the output is interpreted as a probabilistic risk score reflecting manipulation-related risk. This interpretation is consistent with the screening-oriented objective of the study, where the model is used to prioritize high-risk observations. **(Page 15, Section 4.1, lines 341–343)**

First, constrained by public data availability and labels constructed from ex post regulatory penalty documents, the model does not incorporate transaction-level order or trade information and therefore primarily captures statistical associations rather than directly identifying specific manipulative actions. Accordingly, the model output should be interpreted as a probabilistic risk score for candidate review and prioritization, and should be considered together with regulatory judgment and contextual verification. **(Page 31, Section 5, lines 704–708)**

**Comment 5:**

*A section with formal research hypotheses is missing. For a study using supervised learning, the research questions should at least be formulated as testable propositions.*

**Response 5:**

Thank you for this insightful comment. We agree that the research question should be formulated in a testable manner.

In the revised manuscript, we have clarified the central proposition of the study in Section 3.1. Because this study is designed as a supervised risk assessment task rather than a causal hypothesis-testing study, we do not formulate directional causal hypotheses in the traditional H1/H2 format. Instead, we formulate the research objective as a testable proposition: whether manipulation risk can be systematically captured by observable joint deviations across disclosure behavior, sentiment diffusion, and market responses.

This proposition is directly evaluated in the empirical analysis. Specifically, the supervised learning framework tests whether multi-source feature representations improve manipulation-risk ranking and high-risk coverage relative to structured-feature and text-only baselines. The evaluation metrics, including AUC, PR-AUC, Recall@10%, Recall@20%, and the capacity-based screening utility analysis, provide empirical evidence for this proposition.

We have clarified this point in Section 3.1 and aligned the empirical evaluation with this testable proposition.

**Amendments in the Manuscript:**

Based on this perspective, the present study examines whether manipulation risk can be systematically captured by observable joint deviations across disclosure behavior, sentiment diffusion, and market responses. **(Page 8, Section 3.1, lines 189–191)**

**Comment 6:**

*Robustness checks are missing: changing the training period, alternative label binarization thresholds, excluding certain industries. Without these, it is difficult to assess the generalizability of the results.*

**Response 6:**

Thank you for this important methodological comment. We understand the reviewer’s concern that the robustness of the empirical results should be examined more carefully.

In the revised manuscript, we added several complementary robustness and validation analyses. First, we introduced an alternative sentiment measurement using a Chinese transformer sentiment classifier to replace the original lexicon-based announcement sentiment and investor sentiment variables. The results remain close to the main specification, indicating that the findings are not driven by the specific sentiment measurement method.

Second, we added a feature ablation analysis excluding CAR from the structured numerical features. This test clarifies the contribution of CAR and shows that, although CAR improves model performance, the fusion model still retains meaningful risk ranking capacity after CAR is removed.

Third, we added a chronological validation in which the models are trained on 2015–2020 observations, validated on 2021 observations, and evaluated on 2022–2023 observations. This test assesses whether the model remains informative when the evaluation period moves forward in time.

Regarding alternative label thresholds, the labels in this study are not generated by a numerical cutoff, but are constructed from violation intervals disclosed in CSRC administrative penalty documents and retrospectively mapped to the firm–month level. Therefore, changing a label threshold is not directly applicable to the present labeling strategy. Overall, these revisions provide additional evidence on measurement robustness, feature dependence, and temporal generalizability.

**Amendments in the Manuscript:**

In Section 4.3, we added three robustness and validation analyses: alternative sentiment measurement, CAR feature ablation, and temporal validation. These analyses examine whether the main findings are sensitive to sentiment measurement, dependence on CAR, and temporal sample partitioning. **(Pages 27–30, Section 4.3.1–4.3.3, lines 619-683)**

**Comment 7:**

*The assertion that traditional methods "have become insufficient" is formulated declaratively (lines 11–12 in parentheses here and below), without supporting specific references. This thesis requires substantiation in the abstract—either a reference or a brief indication of the scale of the problem (e.g., the volume of identified manipulations or investor losses).*

**Response 7:**

Thank you for this insightful comment. We agree that the original statement in the abstract was formulated too declaratively and did not provide sufficient support.

In the revised manuscript, we have addressed this concern in two ways. First, we have rewritten the opening sentence of the abstract to adopt a more cautious and academically grounded expression, avoiding an unsupported claim about the insufficiency of traditional methods. Second, we have strengthened the empirical motivation of the study in the Introduction by incorporating recent regulatory evidence from China’s A-share market, including enforcement statistics and the associated economic impact of manipulation-related violations. This addition provides concrete context for the scale and practical relevance of the problem.

These revisions provide a clearer empirical basis for the research motivation by linking the abstract statement with supporting regulatory evidence presented in the Introduction.

**Amendments in the Manuscript:**

Against the backdrop of increasingly diversified and concealed forms of stock market manipulation, approaches based solely on trading data or financial indicators face growing limitations in complex and information-intensive market environments. **(Page 2, Abstract, lines 11-13)**

Recent regulatory evidence indicates that stock market manipulation remains a persistent concern in China’s A-share market. For example, the China Securities Regulatory Commission (CSRC) reported that it investigated 701 violation cases in 2025, including 85 cases of market manipulation, with total fines and confiscations exceeding 15 billion yuan. These findings highlight the continued prevalence and substantial economic impact of manipulation activities. **(Page 2, Section 1, lines 29–33)**

**Comment 8:**

*The model's superiority in terms of AUC, PR-AUC, and Recall@10%/20% metrics is claimed, but specific numerical values are not provided. This is a significant drawback—the reader cannot assess the practical significance of the authors' contribution.*

**Response 8:**

Thank you for this comment. We agree that the practical significance of the model should be more clearly reflected in the abstract. In the revised manuscript, we have added representative quantitative results, including AUC and PR-AUC, and explicitly clarified the improvements in Recall@10% and Recall@20% for high-risk screening. Detailed results remain presented in Table 3 in the main text.

**Amendments in the Manuscript:**

Empirical results show that the fusion model consistently outperforms single-source baselines, achieving an AUC of 0.8811 and a PR-AUC of 0.6943, along with substantial improvements in Recall@10% and Recall@20% for high-risk screening. **(Page 2, Abstract, lines 18-20)**

**Comment 9:**

*The term "multi-source coupling nature" (21) is the author's own neologism, which has not gained currency in the literature. It is necessary to either provide a clear definition or replace the term with a standard one.*

**Response 9:**

Thank you for this helpful comment. We agree that the term “multi-source coupling nature” was not sufficiently standard and may have caused ambiguity.

In the revised manuscript, we have replaced this expression with a more precise and commonly used formulation. Specifically, we now describe the theoretical implication as the complementary role of heterogeneous information sources, including disclosure, sentiment, and trading-related signals, in characterizing manipulation risk. This revision avoids the use of a non-standard term and improves consistency with the conceptual framework developed in the manuscript.

**Amendments in the Manuscript:**

Theoretically, this study highlights the complementary role of heterogeneous information sources, including disclosure, sentiment, and trading-related signals, in characterizing manipulation risk. In practice, it provides a feasible data-driven pathway for risk monitoring and tiered regulatory screening. **(Page 2, Abstract, lines 22-24)**

**Comment 10:**

*The introduction too quickly moves from the problem statement to the gaps in the literature, bypassing the rationale for the practical significance of the study for regulators (26–33). The question remains unanswered: how great is the damage from manipulation in the Class A share market? Without this, the motivation for the study appears declarative.*

**Response 10:**

Thank you for this important comment. We agree that the practical significance of the study for regulators should be more clearly articulated.

In the revised manuscript, we have strengthened the motivation in the Introduction by incorporating recent regulatory evidence from China’s A-share market. Specifically, we refer to recent enforcement data released by the CSRC, which highlights the continued occurrence and economic impact of manipulation-related violations. This addition provides empirical context for the relevance and urgency of the research problem. Additionally, we have clarified the practical constraint faced by regulators, namely that supervisory resources are limited and cannot be applied uniformly across all market participants. This makes effective risk prioritization essential for front-end screening. We therefore emphasize that approaches based on single-source data provide limited support for reliable risk prioritization in complex and information-intensive environments, which motivates the need for a multi-source, data-driven framework.

These revisions have been incorporated in the first paragraph of the Introduction.

**Amendments in the Manuscript:**

Against the backdrop of highly informatized capital markets and increasingly complex trading mechanisms, stock market manipulation has evolved toward more concealed and strategic forms, posing persistent challenges to market fairness and regulatory effectiveness [1]. Recent regulatory evidence indicates that stock market manipulation remains a persistent concern in China’s A-share market. For example, the China Securities Regulatory Commission (CSRC) reported that it investigated 701 violation cases in 2025, including 85 cases of market manipulation, with total fines and confiscations exceeding 15 billion yuan. These findings highlight the continued prevalence and substantial economic impact of manipulation activities. Traditional manipulation typically relies on concentrated trading to directly influence the price formation process. However, with the expansion of information dissemination channels and the changing structure of investors, manipulative activities are increasingly accompanied by abnormal information disclosure, fluctuations in market sentiment, and interconnected price reactions [2,3]. Under the practical constraint of limited regulatory resources, regulators must allocate attention across a large number of market participants and prioritize observations that exhibit elevated risk levels. In this context, approaches based on single-source data provide limited support for reliable risk prioritization, motivating the need for data-driven frameworks based on observable multi-source signals. **(Page 2-3, Section 1, lines 27–39)**

**Comment 11:**

*The description of existing work in the introduction (lines 35–46) duplicates the content of Section 2 (literature review). This creates structural redundancy. It is recommended to limit the introduction to the problem statement and the gap, moving the detailed literature review to the appropriate section.*

**Response 11:**

Thank you for this valuable comment. We agree that the original version of the Introduction contained elements that overlapped with the detailed literature review in Section 2, which may have led to structural redundancy.

In the revised manuscript, we have refined the Introduction to focus more clearly on the problem context and the research gap, while reducing descriptive elements of prior studies that are more appropriately presented in the literature review. Specifically, the revised paragraph provides a concise synthesis of prior work to motivate the research question and highlight the key limitation, namely that current approaches largely examine different information sources separately and lack a unified framework to capture manipulation risk as a coordinated process across disclosure behavior, sentiment diffusion, and market responses.

This revision improves the distinction between the Introduction and Section 2, ensuring that the Introduction emphasizes research motivation and gap identification, while the detailed literature review is presented in Section 2.

**Amendments in the Manuscript:**

Existing research on stock market manipulation primarily focuses on detecting trading anomalies. Early studies relied on market variables such as prices and trading volumes, employing regression analysis, statistical tests, or machine learning techniques to characterize abnormal trading patterns. Nevertheless, such approaches face limitations in capturing increasingly concealed and information-based manipulation patterns, particularly when relying on single-source data [4]. With the improvement of disclosure systems and the rise of social media, recent studies have incorporated textual information and public sentiment into analysis, showing that disclosure tone, investor attention, and online discussions are associated with stock price dynamics and risk exposure [5,6]. Natural language processing–based methods have further extended manipulation analysis into the informational domain [7,8]. Despite these advances, existing approaches typically examine different information sources separately. Differences in temporal granularity, update frequency, and data representation further hinder their integration within a unified analytical framework. As a result, current models remain limited in capturing manipulation risk as a coordinated process integrating disclosure behavior, sentiment diffusion, and market responses, and continue to focus primarily on binary classification rather than risk ranking and tiered screening. **(Page 3, Section 1, lines 40–51)**

**Comment 12:**

*The three stated "contributions" (lines 53–60) are not clearly stated. In particular, the second contribution ("incorporates deep semantic features") is applicable to any work using BERT. It is necessary to specify the novelty: how exactly does this architecture outperform its predecessors when applied to the task of manipulation detection?*

**Response 12:**

Thank you for this important comment. We agree that the original version of the manuscript did not sufficiently clarify the specific contributions of the study, especially the novelty of the proposed modeling design.

In the revised manuscript, we have refined the contribution statements in the Introduction to make the novelty and research positioning more explicit. Specifically, we have clarified the contributions from three aspects: the multi-source risk assessment framework, the BERT–MIL architecture for announcement modeling, and the probabilistic risk scoring perspective for regulatory screening.

First, we emphasize that the study develops a unified multi-source framework that captures manipulation risk through the joint dynamics of information disclosure, sentiment diffusion, and market trading behavior under a consistent temporal structure. Second, we have clarified that the methodological contribution lies in introducing a BERT–MIL architecture, which models multiple announcements within the same period as a set and captures heterogeneous and unevenly distributed information signals that may be attenuated under conventional document-level aggregation, thereby improving the representation of sparse but risk-relevant textual cues. Third, we revise the contribution statement to highlight that the study formulates stock market manipulation analysis as a probabilistic risk assessment problem, supporting risk ranking and tiered screening in regulatory practice, while further enhancing interpretability through SHAP.

**Amendments in the Manuscript:**

Compared with prior research, this study offers three main contributions. First, it develops a unified multi-source framework that captures manipulation risk through the joint dynamics of information disclosure, sentiment diffusion, and market trading behavior under a consistent temporal structure. Second, it introduces a BERT–MIL architecture that models multiple announcements within the same period as a set and captures heterogeneous and unevenly distributed information signals that are often diluted in conventional semantic analysis. Third, it formulates stock market manipulation analysis as a probabilistic risk assessment problem that supports risk ranking and tiered screening in regulatory practice, and further enhances interpretability through SHAP (SHapley Additive exPlanations). **(Pages 3-4, Section 1, lines 60–67)**

**Comment 13:**

*In the literature review, the authors mechanically list works in each subsection (62–90) without providing a critical discussion. There is no comparison of competing approaches, no indication of contradictions between the results of different authors, and no explanation of why existing solutions are insufficient for the given context (Chinese Market A).*

**Response 13:**

Thank you for this important and constructive comment. We agree that the original Section 2.1 was too close to a sequential summary of prior studies and did not sufficiently compare different approaches to stock market manipulation risk assessment.

In the revised manuscript, we revised Section 2.1 to provide a more analytical discussion. The revised section now develops the literature along four connected lines: theoretical explanations of manipulation mechanisms, data-driven identification based on trading anomalies, heterogeneity across manipulation strategies, and the growing role of information-related signals. This structure allows us to compare the scope and limitations of different approaches rather than simply listing previous studies.

Specifically, we clarify that early studies based on market microstructure and information asymmetry explain the economic logic of manipulation, but provide limited guidance for constructing operational indicators for large-scale screening. Data-driven and classification-based studies improve empirical detectability, especially in the Chinese regulatory context using CSRC enforcement cases, but they remain largely organized around trading anomalies and binary detection. More recent studies on manipulation heterogeneity, AI-based detection, and RegTech further indicate that contemporary manipulation may combine trading activity, information release, investor attention, and sentiment amplification. This creates a methodological limitation for approaches based on a single manipulation pattern or a single data source.

We also strengthened the connection to China’s A-share market context. Because manipulation risk in this setting may be reflected not only in trading outcomes but also in disclosure behavior and information diffusion, existing single-source or binary-detection approaches are insufficient for the screening-oriented objective of this study. The revised Section 2.1 therefore provides a clearer rationale for integrating disclosure, public sentiment, and market trading signals within a probabilistic multi-source risk assessment framework.

**Amendments in the Manuscript:**

2.1	Risk Assessment of Stock Market Manipulation

Research on stock market manipulation has evolved from theoretical explanations of manipulation mechanisms to empirical identification using observable market anomalies. Early studies examined manipulation from the perspectives of market microstructure and information asymmetry, showing how manipulators may profit by exploiting frictions in the price formation process [3,9]. While these studies clarify the economic logic of manipulation, they provide limited guidance for constructing operational indicators for large-scale empirical screening and risk assessment.

With the increasing availability of trading data and regulatory disclosures, empirical research has increasingly operationalized manipulation as a data-driven identification problem. In the Chinese regulatory context, Liu et al. used administrative penalty cases disclosed by the CSRC (China Securities Regulatory Commission) to develop machine learning models and showed that trading intensity, price volatility, and transaction-structure variables have discriminative value for identifying manipulation [5]. Related studies have further examined classification methods, multidimensional abnormal trading indicators, and price-pattern-based approaches for detecting potential manipulation, insider trading, or atypical stock transactions [10-13]. These studies improve the empirical feasibility of manipulation analysis by translating manipulation into measurable features and classification tasks. However, their evidence is still largely organized around trading anomalies and binary detection, providing limited insight into manipulation risk as a probabilistic state shaped by disclosure behavior, sentiment diffusion, and market responses.

Building on this foundation, later studies have paid increasing attention to heterogeneity across manipulation strategies and the evolution of detection frameworks. Allen and Gorton offered an early theoretical distinction among action-based manipulation, trade-based manipulation, and information-based manipulation [3]. Siering et al. further refined this perspective by showing that different manipulation techniques may involve distinct trading paths, information channels, and market responses [14]. Recent reviews and surveys of manipulation detection emphasize multi-feature learning rather than single-rule screening [4,15]. RegTech studies also position automated risk identification as an important direction for financial supervision [16,17]. This methodological evolution reflects a practical challenge: contemporary manipulation may combine trading activity, information release, investor attention, and sentiment amplification. Models built around a single manipulation pattern or a single data source may therefore have limited generalizability across risk scenarios.

Meanwhile, the informational dimension has attracted growing attention in manipulation risk assessment. Prior studies indicate that textual tone, disclosure similarity, and financial textual signals are associated with firm risk and abnormal market behavior [6,7,18]. Recent evidence further links investor attention and public information manipulation to stock price manipulation and abnormal price reactions [19,20]. These studies suggest that manipulation risk may be reflected not only in trading outcomes, but also in information disclosure and diffusion. Yet existing research often treats trading, textual, and sentiment signals separately and remains centered on binary detection. This limitation motivates a unified multi-source framework that integrates disclosure, public sentiment, and market trading signals for probabilistic risk assessment. **(Pages 4-5, Section 2.1, lines 71–102)**

**Comment 14:**

*Citing Allen & Gorton (1992)—a work more than 30 years old—as a key theoretical framework without discussing more recent theoretical developments diminishes the relevance of the review (77–83). It is necessary to demonstrate how theoretical perspectives have evolved.*

**Response 14:**

Thank you for this important and helpful comment. We agree that Allen and Gorton (1992) should not be presented in isolation, and that the literature review could better situate this early theoretical contribution within the subsequent development of manipulation research.

In the revised manuscript, we have revised Section 2.1 to clarify the evolution of theoretical and methodological perspectives on stock market manipulation. Allen and Gorton (1992) is now positioned as an early theoretical study that distinguishes among action-based, trade-based, and information-based manipulation. We then extend the discussion to later studies that refine this typological perspective and emphasize the heterogeneity of manipulation strategies, including differences in trading paths, information channels, and market responses.

We also incorporated more recent studies on AI-based manipulation detection and regulatory technology. These studies indicate that manipulation detection has moved beyond single-rule or single-pattern screening toward multi-feature learning and automated risk identification. This revision allows the literature review to present a clearer progression from early theoretical explanations to contemporary data-driven and RegTech-oriented approaches.

The revised discussion further connects this evolution to the motivation of our study. Because contemporary manipulation may combine trading activity, information release, investor attention, and sentiment amplification, models based on a single manipulation pattern or a single data source may have limited generalizability across risk scenarios. This provides a stronger basis for our multi-source, screening-oriented risk assessment framework.

**Amendments in the Manuscript:**

Building on this foundation, later studies have paid increasing attention to heterogeneity across manipulation strategies and the evolution of detection frameworks. Allen and Gorton offered an early theoretical distinction among action-based manipulation, trade-based manipulation, and information-based manipulation [3]. Siering et al. further refined this perspective by showing that different manipulation techniques may involve distinct trading paths, information channels, and market responses [14]. Recent reviews and surveys of manipulation detection emphasize multi-feature learning rather than single-rule screening [4,15]. RegTech studies also position automated risk identification as an important direction for financial supervision [16,17]. This methodological evolution reflects a practical challenge: contemporary manipulation may combine trading activity, information release, investor attention, and sentiment amplification. Models built around a single manipulation pattern or a single data source may therefore have limited generalizability across risk scenarios. **(Page 4-5, Section 2.1, lines 87–95)**

**Comment 15:**

*Section 2.2 (91–122) is devoted to the diffusion of public sentiment, but its connection to the main objective of the study (assessing the risk of manipulation) is weak. The authors cite studies on the prediction of returns and volatility (97–99, 102–103), but do not explain why these mechanisms are relevant specifically for detecting manipulative behavior, rather than simply information shocks.*

**Response 15:**

Thank you for this insightful comment. We agree that the original discussion in Section 2.2 did not sufficiently explain why public sentiment diffusion is relevant to manipulation risk assessment, rather than only to general market reactions to information shocks.

In the revised manuscript, we have strengthened the logical connection between sentiment diffusion and manipulation risk. We clarify that public sentiment is not treated as direct evidence of manipulation. Instead, it is regarded as an information-diffusion signal that may become risk-relevant when investor attention, emotional framing, or online discussions are amplified, distorted, or unusually concentrated. This distinction is important because the objective of this study is not to infer manipulation from sentiment alone, but to assess whether sentiment dynamics provide complementary information when examined together with disclosure behavior and market responses.

We also revised Section 2.2 to distinguish between two strands of literature. The first shows that news sentiment, media-based sentiment, and online forum sentiment are associated with market outcomes such as returns and volatility. The second is more directly related to manipulation risk, showing that rumor diffusion, public information manipulation, and distorted information flows can interact with investor reactions and market feedback. By making this distinction explicit, the revised section explains why sentiment diffusion mechanisms are relevant to manipulation risk assessment rather than merely to ordinary information shocks.

Finally, we revised the closing paragraph of Section 2.2 to emphasize that existing studies often examine sentiment, disclosure, and market responses separately. This revision supports our use of public sentiment as an information-diffusion signal that complements disclosure behavior and market responses within the multi-source risk assessment framework.

**Amendments in the Manuscript:**

2.2	Information Dissemination and Public Sentiment Diffusion in Stock Markets

With the diversification of information channels and the rise of social media, market reactions are no longer shaped only by formal disclosures and traditional news reports. The speed, tone, and pathway of information diffusion also affect how investors process signals and adjust expectations. Prior research suggests that investor responses to information shocks are not fully rational; public sentiment diffusion, emotional framing, and social transmission can shape investor decisions and market dynamics [21]. Wang et al. further show that sentiment changes in online forums are related to stock returns, indicating that investor-generated discussions can enter the price formation process [22].

Related studies further examine how sentiment embedded in news and media content affects market outcomes. Gambarelli and Muzzioli examined the relationship between news tone and stock returns, showing that positive news sentiment is associated with short-term price increases, while negative sentiment is associated with greater market volatility [8]. Obaid and Pukthuanthong further show that machine-learning-based sentiment measures extracted from news images contain information about investor sentiment and market pricing [23]. These studies establish the market relevance of sentiment, but they mainly examine sentiment as a reaction to information, rather than as a channel through which risk may be amplified.

Research on social networks further shows that information effects depend on diffusion structure. Teplova et al. reported that both information attention and sentiment dynamics within social networks influence retail investors’ trading decisions, reflecting pronounced attention-driven characteristics [24]. In manipulation contexts, this diffusion structure matters because attention bursts and concentrated discussions may reinforce abnormal price movements. Some studies have further explored the role of information manipulation within sentiment diffusion processes. Tan et al. provided empirical evidence that the spread of false information can distort investor behavior and intensify stock price fluctuations [25]. Evidence on public information manipulation and rumor verification further suggests that distorted information flows can interact with market feedback and investor reactions [26]. Together, these findings suggest that sentiment diffusion is relevant to manipulation risk when it is amplified, distorted, or strategically used.

Overall, existing research shows that information and public sentiment are important for understanding market reactions and manipulation risk. Methodologically, this literature has moved from single-source sentiment analysis toward broader perspectives that consider network effects, emotional polarization, and information dissemination. Nevertheless, prior studies remain largely focused on single-channel informational analyses and lack integrated quantitative frameworks that jointly capture sentiment diffusion and market responses, particularly in the context of manipulation risk assessment within complex financial markets. **(Pages 5-6, Section 2.2, lines 103–131)**

**Comment 16:**

*Section 2.3, on multi-source data fusion (123–159), is primarily descriptive. It lacks a critique of the methodological approaches used in the cited studies. The authors should clearly indicate which methodological limitations of previous studies this article addresses.*

**Response 16:**

Thank you for this valuable comment. We agree that the original discussion of multi-source data fusion was more descriptive than analytical and did not sufficiently explain the methodological gap that motivates our framework.

In the revised manuscript, Section 2.3 has been reorganized into three related streams: the expansion of risk assessment variables from single-source indicators to multi-source representations; the complementary value of textual data and the broader information environment; and the joint modeling of textual information, public sentiment, and market behavior for regulatory decision-making.

We also added a clearer discussion of the limitations of existing studies. The revised section clarifies that feature expansion alone does not fully address how heterogeneous signals should be aligned and fused for risk ranking rather than simple classification. It also notes that much of the evidence on textual and information-environment data is developed in financial distress, fraud, or general risk settings, while the interaction between textual signals and market-trading responses remains less developed in manipulation risk assessment. Finally, we clarify that many fusion studies emphasize performance gains or general decision support, whereas the role of fusion in probabilistic manipulation risk assessment and screening remains insufficiently specified.

These revisions allow the literature review to better explain why our framework is needed. We clarified that the contribution lies not in adding data sources per se, but in jointly representing disclosure, public sentiment, and market trading signals for manipulation risk ranking and tiered screening.

**Amendments in the Manuscript:**

2.3	Applications of Multi-Source Data Fusion in Financial Regulation

As financial systems become more complex and risk patterns evolve, financial regulation increasingly addresses interconnected issues such as market manipulation, financial fraud, disclosure violations, investor behavior, and systemic risk transmission. In this context, prior studies suggest that reliance on a single data source, such as trading records or financial statements, provides limited support for forward-looking and interpretable risk assessment. Multi-source data fusion has therefore become an important direction in financial regulatory technology (RegTech) [4,19].

The first stream of research expands risk assessment variable systems from single-source indicators to multi-source representations. Early financial risk assessments primarily relied on financial and trading indicators, employing regression models or structural equation modeling to capture abnormal trading activities and price distortions [27,28]. As regulatory problems become more information-intensive, transaction-level or financial indicators alone are less able to capture concealed and information-based risks. Yuan et al. showed that integrating financial indicators with unstructured information improves the stability and interpretability of financial fraud risk identification [29]. Using CSRC enforcement samples, Liu et al. further demonstrated that multidimensional feature systems outperform single trading features in manipulation detection [5]. These studies justify feature expansion, but they do not fully address how heterogeneous signals should be aligned and fused for risk ranking rather than simple classification.

The second stream of research focuses on the complementary value of textual data and the broader informational environment in regulatory contexts. Prior studies suggest that annual report texts, disclosure tone, and reporting patterns can reveal firms’ underlying risk behaviors. Through the classification of SEC (U.S. Securities and Exchange Commission) comment-letter texts, Ryans showed that regulatory documents themselves contain important risk signals [30]. Chen et al. incorporated semantic features derived from annual reports into machine learning models and improved the identification of financial distress and related risks [31]. Examining the consistency between public sentiment and textual tone, Yen et al. found that public sentiment and semantic textual features can serve as important leading indicators of financial risk [32]. These studies demonstrate that textual and information environment data capture risk signals that are not directly observable from transaction outcomes alone. However, most of this evidence is developed in financial distress, fraud, or general risk settings, and the interaction between textual signals and market-trading responses remains less developed in manipulation risk assessment.

The third stream moves toward the joint modeling of textual information, public sentiment, and market behavior for regulatory decision-making. Yao et al. demonstrated the advantages of multi-source fusion across the data, model, and decision layers, showing that integrating textual and trading data can reduce the noise of single-source signals [33]. Cao et al. proposed a human–machine collaborative analytical paradigm, emphasizing that model outputs should complement regulatory experience and expert judgment to enhance practical usability [34]. Wang and Zheng further highlighted that multi-source analysis helps characterize the links among information manipulation, institutional changes, and market responses [35]. Yet many fusion studies emphasize performance gains or decision support in broad financial-risk settings, while the role of fusion in probabilistic manipulation risk assessment and screening remains insufficiently specified.

Taken together, existing research supports the value of multi-source information in financial regulation, but important gaps remain in how heterogeneous disclosure, sentiment, and trading signals are jointly represented for manipulation ranking. Building on this literature, the present study integrates announcement texts, public sentiment, and market trading information within a unified risk assessment framework to evaluate their complementary value in probabilistic risk scoring and tiered screening. **(Pages 6-8, Section 2.3, lines 132–169)**

**Comment 17:**

*Section 3.1 purports to provide a theoretical justification (162–186), but in reality represents a narrative description of the manipulation process without a formal model or testable hypotheses. If the study is empirical in nature, it is necessary to explicitly formulate at least testable working hypotheses (H1, H2, etc.) for each group of variables.*

**Response 17:**

Thank you for this constructive comment. We agree that the original version of Section 3.1 was primarily descriptive and could be strengthened in terms of theoretical grounding.

In the revised manuscript, we have substantially revised Section 3.1 to make its theoretical role clearer. First, we reformulated the research objective as a testable proposition by examining whether manipulation risk can be systematically captured by observable joint deviations across disclosure behavior, sentiment diffusion, and market responses. Second, we clarified the conceptual basis of the explanatory variables by linking the three feature groups to three corresponding information dimensions: corporate disclosures, public sentiment, and market responses.

This formulation is consistent with the supervised learning framework adopted in this study. Instead of specifying directional hypotheses for each individual variable, the revised section evaluates whether these feature groups jointly provide a measurable representation of manipulation risk. To strengthen the theoretical grounding, we also incorporated additional literature on information environments, investor attention, rumor diffusion, and market feedback mechanisms.

We have revised Section 3.1 accordingly and clarified how the theoretical discussion supports the variable system constructed in Section 3.2.

**Amendments in the Manuscript:**

Based on this perspective, the present study examines whether manipulation risk can be systematically captured by observable joint deviations across disclosure behavior, sentiment diffusion, and market responses. **(Page 8, Section 3.1, lines 189–191)**

The theoretical basis of this proposition lies in the complementary roles of three information dimensions. First, formal disclosures issued by listed firms may reflect how firms shape, frame, or communicate information to the market. Disclosure timing, tone, readability, and reporting patterns can influence investor interpretation and may reveal abnormal information presentation associated with elevated risk [6,30,38]. Second, public sentiment generated by market participants captures investor attention, discussion intensity, and the diffusion of expectations in online information environments. Prior studies suggest that investor sentiment, rumors, and managerial responses on interactive platforms can affect market reactions and amplify abnormal information diffusion [21,22,25]. Third, market responses capture the realization of risk signals through returns, volatility, trading intensity, and market value changes. Existing evidence shows that public information manipulation and trading-based manipulation can affect asset prices through feedback mechanisms and abnormal trading responses [20,39]. These three dimensions correspond to the main feature groups used in this study: announcement-text features, public sentiment features, and market trading features. They do not represent isolated hypotheses for individual variables, but jointly define a risk representation framework. From a dynamic perspective, manipulation risk may be reflected when disclosure patterns, sentiment diffusion, and market responses reinforce one another within the same risk process. This multi-source perspective therefore provides the theoretical basis for the variable system constructed in the following section. **(Page 9, Section 3.1, lines 193–206)**

**Comment 18:**

*The ad sentiment index (formula 1) is calculated using the CFSD dictionary (199–208).*

*However, the authors do not justify the choice of this dictionary, do not compare it with alternatives (for example, financially-oriented versions of LIWC or specialized dictionaries for the Chinese stock market), and do not discuss its limitations when applied to corporate announcements.*

**Response 18:**

Thank you for this important and constructive comment. We agree that the original manuscript did not sufficiently justify the use of the sentiment dictionary, nor did it adequately discuss the limitations of the dictionary-based sentiment index when applied to corporate announcements.

In the revised manuscript, we have clarified the role of the sentiment dictionary in the overall modeling framework. The lexicon-based sentiment score in Eq (1) is retained as an interpretable structured proxy for announcement tone, rather than as a substitute for deep semantic modeling. This proxy is useful for constructing firm-month numerical indicators and is complementary to the BERT + MIL text branch in the final fusion model, which learns contextual semantic representations directly from announcement texts. In this sense, the lexicon measure captures an interpretable tone dimension, while the BERT + MIL module captures richer semantic information that cannot be reduced to word counts.

We have also strengthened the justification for using a Chinese financial sentiment lexicon. Specifically, we now explain that financial sentiment measurement is sensitive to language and domain context, which supports the use of Chinese financial sentiment resources rather than general-purpose dictionaries when analyzing Chinese financial texts. At the same time, we explicitly acknowledge the limitations of word count measures. Their representation of contextual meaning depends on dictionary coverage and word usage, and they may not fully capture context, implicit tone, or nuanced expressions in standardized corporate announcements.

To further address the concern that the dictionary-based measure may be relatively shallow, we added a robustness check using alternative sentiment measures generated by a Chinese transformer sentiment classifier motivated by the FinBERT framework. In this test, the original announcement sentiment variable (ann_sent) and investor forum sentiment variable (sent_mean) are replaced by transformer-generated sentiment measures, while the remaining structured variables, sample, labels, announcement texts, and fusion model architecture remain unchanged. The replacement affects only the structured sentiment variables and does not alter the announcement representation module of the fusion model.

The robustness results show that the fusion model retains comparable performance under this alternative sentiment specification, with an average AUC of 0.8813, a PR-AUC of 0.6853, Recall@10% of 0.4505, and Recall@20% of 0.6889. These results are close to the main specification reported in Table 3, suggesting that the empirical findings are not dependent on the lexicon used to construct the sentiment variables.

**Amendments in the Manuscript:**

In Section 3.2.1, we revised the explanation of the announcement sentiment variable to clarify the dictionary choice, the role of the sentiment score, and its limitation:

Methodologically, sentiment words are identified using a Chinese financial sentiment lexicon constructed with reference to prior Chinese financial sentiment dictionaries [40].

……

In addition, financial sentiment measurement is sensitive to language and domain context, which supports the use of Chinese financial sentiment resources when analyzing Chinese financial texts [41]. In this study, the sentiment score is used as a transparent and reproducible proxy for announcement tone. Given the inherent limits of word count measures in representing contextual meaning, the sensitivity of this measurement choice is further examined in Section 4.3 using alternative sentiment measures constructed with a transformer model. **(Page 10, Section 3.2.1, lines 220–232)**

**Additional references:**

40. Jiang F, Meng L, Tang G. Media Textual Sentiment and Chinese Stock Return Predictability. China Economic Quarterly. 2021;21(4):1323–1344. doi:10.13821/j.cnki.ceq.2021.04.10.

41. Du Z, Huang AG, Wermers R, Wu W. Language and domain specificity: A Chinese financial sentiment dictionary. Review of Finance. 2022;26: 673–719. doi:10.1093/rof/rfab036

We also added Section 4.3.1 to compare the lexicon-based sentiment construction with an alternative sentiment measurement using a Chinese transformer sentiment classifier:

4.3.1	Alternative Sentiment Measurement

The sentiment score in Eq (1) is constructed from a Chinese financial sentiment lexicon and serves as an interpretable proxy for announcement tone. Measures based on lexicons are transparent and reproducible, but their representation of contextual meaning depends on dictionary coverage and word usage. To assess whether the empirical results are sensitive to this measurement choice, we construct alternative sentiment variables using a Chinese transformer sentiment classifier motivated by the FinBERT framework [47].

The classifier produces sentiment class probabilities, and the alternative sentiment score is defined as P(positive) − P(negative). The resulting sentiment scores are aggregated to the firm–month level and used to replace the original announcement sentiment variable (ann_sent) and investor forum sentiment variable (sent_mean). All remaining structured variables, as well as the sample, labels, announcement texts, and fusion model architecture, remain unchanged. The replacement affects only the structured sentiment variables and does not alter the announcement representation module of the fusion model.

As reported in Table 6, the fusion model retains comparable performance under the alternative sentiment specification. Across five random seeds, the alternative specification achieves an average AUC of 0.8813, a PR-AUC of 0.6853, Recall@10% of 0.4505, and Recall@20% of 0.6889. These results are close to those of the main specification reported in Table 3, indicating that the empirical findings are not materially driven by the specific lexicon-based construction of the sentiment variables. **(Pages 27–28, Section 4.3.1, lines 623–641)**

Table 6 was also added to report the robustness results under the alternative sentiment measurement.

**Table 6. Robustness check using alternative sentiment measurement.**

| **Specification** | **Sentiment measurement** | **AUC** | **PR-AUC** | **Recall@10%** | **Recall@20%** |
| --- | --- | --- | --- | --- | --- |
| **Main specification** | Chinese financial sentiment lexicon | 0.8811±0.0063 | 0.6943±0.0158 | 0.4545±0.0294 | 0.6788±0.0166 |
| **Alternative specification** | Chinese transformer sentiment classifier | 0.8813±0.0088 | 0.6853±0.0591 | 0.4505±0.0477 | 0.6889±0.0330 |

**Note:** The main specification is repeated from Table 3 for reference. In the alternative specification, ann_sent and sent_mean are replaced by sentiment measures generated using a Chinese transformer sentiment classifier. Other structured variables, labels, announcement texts, and fusion model architecture remain unchanged.

**Additional reference:**

47. Huang AH, Wang H, Yang Y. FinBERT: A large language model for extracting information from financial text. Contemporary Accounting Research. 2023;40(2):806–841. doi:10.1111/1911-3846.12832.

**Comment 19:**

*The readability index (formula 2) is an adaptation of the Fog Index (209–218). The authors do not discuss its validity for Chinese: the Fog Index was developed for English and is based on a syllabic structure incompatible with Chinese morphology. There is no justification for choosing this measure for Chinese text—this is a serious methodological flaw.*

**Response 19:**

Thank you for pointing out this important methodological issue. We agree that the original Gunning Fog Index was developed for English texts and that its syllable-based definition of complex words is not directly applicable to Chinese. We also acknowledge that the original manuscript did not explain this linguistic adaptation clearly enough, which may have created the impression that the English Fog Index was directly applied to Chinese announcement texts.

In the revised manuscript, we have clarified that the measure used in Equation (2) is not a direct application of the original English Gunning Fog Index, but a Chinese-adapted textual complexity index. Drawing on Chinese financial-text readability studies, particularly Li et al. [44], we construct the index using average sentence length and the complex-word ratio. Specifically, the complex-word ratio is calculated as the proportion of segmented Chinese words containing three or more Chinese characters, rather than using syllable counts. This adaptation is more appropriate for Chinese financial texts because Chinese word complexity cannot be measured using the same syllable-based criterion as English.

We have revised the explanation of Equation (2) to make this adaptation explicit. We have also revised Table 1 by changing the indicator name from “Announcement readability (Fog index)” to “Chinese-adapted textual complexity index” and by defining it as “Textual complexity measured by average sentence length and complex-word ratio.” These revisions clarify the linguistic basis of the measure and address the applicability of the measure in the Chinese context.

**Amendments in the Manuscript:**

The original Gunning Fog Index was developed for English texts and identifies complex words based on syllable structure, which is not directly applicable to Chinese. Drawing on Chinese financial-text readability studies, particularly Li et al. [44], this study constructs the index based on average sentence length and the complex-word ratio:

$${\text{ann}\text{\_f}\text{og}}_{\text{i}}\text{=}\text{ASL}_{\text{i}}\text{+}\text{CWR}_{\text{i}}$$

(2)

where $\text{ASL}_{\text{i}}\text{ }$denotes the average sentence length, calculated as the total number of segmented words divided by the number of sentences, and $\text{CWR}_{\text{i}}\text{ }$ represents the complex-word ratio, calculated as the proportion of segmented words containing three or more Chinese characters. A higher value of ann_fog indicates greater textual complexity and lower readability. **(Pages 10-11, Section 3.2.1, lines 239–245)**

**Additional reference:**

44. Li C, Jia H, Zhao G, Fu H. Credit Risk Warning of Listed Companies Based on Information Disclosure Text: Empirical Evidence from Management Discussion and Analysis of the Chinese Annual Report. Chinese Journal of Management Science. 2023;31(2):18-29. doi:10.16381/j.cnki.issn1003-207x.2020.2263

In Table 1, we revised the indicator name and definition of ann_fog as follows:

ann_fog — “Chinese-adapted textual complexity index” — “Textual complexity measured by average sentence length and complex-word ratio.” **(Page 14, Table 1)**

**Comment 20:**

*The public sentiment variables (post_cnt, user_cnt, etc.) are taken from the Eastmoney Guba platform (219–243). The authors do not discuss how representative this platform is of the entire investor community. Guba is known to be primarily focused on retail investors, which may systematically bias sentiment estimates relative to institutional participants.*

**Response 20:**

Thank you for this important and helpful comment. We agree that the original manuscript did not sufficiently clarify the representativeness of the Eastmoney Guba data source. Eastmoney Guba is a retail-oriented online forum and should not be interpreted as representing the sentiment of the entire investor community.

In the revised manuscript, we have clarified the role of the Guba-based variables. These variables are not used as comprehensive measures of market-wide investor sentiment. Instead, they are used as proxies for retail-facing public opinion dynamics, investor attention, and informal information dissemination. This interpretation is more consistent with the data source and with the screening-oriented objective of this study.

We consider these retail-facing online signals relevant because attention concentration, discussion intensity, and sentiment fluctuations in public forums may reflect the diffusion of information and expectations among individual investors. At the same time, we acknowledge that this data source has representativeness limitations. Therefore, the sentiment-related results should be interpreted as reflecting observable online public opinion dynamics rather than the sentiment of all market participants.

Accordingly, we revised the data source description in Section 4.2.1 to make this limitation and interpretation clearer.

**Amendments in the Manuscript:**

Regarding data sources, this study integrates multi-source heterogeneous data to characterize market manipulation risk from disclosure, sentiment, and trading dimensions. Corporate announcement data are obtained from the Choice Financial Terminal (Choice) and include both periodic reports and ad hoc disclosures released during the study period, reflecting firms’ formal information disclosure activities. Investor sentiment data are collected from the Eastmoney Guba platform, a retail-oriented online forum, and are used as proxies for retail-facing public opinion dynamics, investor attention, and informal information dissemination. Market trading data are sourced from the China Stock Market and Accounting Research Database (CSMAR), covering indicators such as monthly returns, turnover rates, and market capitalization changes to describe firms’ market trading behavior.  **(Page 20, Section 4.2.1, lines 443–450)**

**Comment 21:**

*When describing the exchange variables (244–272), the authors do not justify the choice of a six-month window for calculating volatility (265–266). Why six months, and not three or twelve? The sensitivity of the results to the choice of window is not discussed.*

**Response 21:**

Thank you for this helpful methodological comment. We agree that the original manuscript did not sufficiently explain the rationale for using a six-month window to calculate volatility.

In the revised manuscript, we have clarified the measurement logic in Section 3.2.3. Since the empirical analysis is conducted at the firm–month level, the volatility variable is intended to capture medium-horizon price instability rather than very short-term fluctuations. A three-month window contains relatively few monthly observations and may be strongly affected by one-off price movements or temporary market noise. By contrast, a twelve-month window may smooth recent abnormal price instability and mix the current risk state with earlier market conditions. The six-month rolling window is therefore used as a balanced specification: it provides more stable dispersion information than a very short window while remaining sufficiently responsive to recent abnormal price movements relevant to manipulation risk assessment.

We have revised the manuscript to make this rationale explicit. Specifically, we now state that volatility is computed as the rolling standard deviation of Mretwd over the most recent six observed months, including the current month, with at least three valid monthly observations required, and that the six-month window is used as a medium-horizon volatility proxy at the monthly frequency.

**Amendments in the Manuscript:**

In this study, volatility is computed as the rolling standard deviation of Mretwd over the most recent six observed months, including the current month, with at least three valid monthly observations required. The six-month window is used as a medium-horizon volatility proxy at the monthly frequency, balancing the need to smooth transitory fluctuations while retaining recent abnormal price instability relevant to manipulation risk. **(Page 13, Section 3.2.3, lines 299–302)**

**Comment 22:**

*The authors use monthly data for the dataset:*

*- this time period does not specify whether manipulation occurred every trading day or just a few days, or even just one day within the period.*

*- regarding investor sentiment: over the monthly observation period, if manipulation occurred and prices rose, positive sentiment and active discussions may be a consequence of the price increase; this correlates with manipulation, but is not directly related to it.*

**Response 22:**

Thank you for this important comment. We agree that monthly data cannot identify whether manipulation occurred on every trading day, only on a few days, or even on a single day within the month. In this study, the monthly label should therefore be interpreted carefully: a violating firm–month indicates that the month overlaps with the violation interval disclosed in the regulatory penalty document, not that manipulative behavior necessarily occurred continuously throughout the entire month.

The firm–month frequency is adopted because the objective of this study is screening-oriented risk assessment rather than intraday or event-level manipulation detection. Corporate announcements, online public sentiment variables, and market trading indicators are aligned at the monthly level to form a unified multi-source panel suitable for periodic risk ranking and candidate review. We acknowledge, however, that this temporal aggregation may smooth short-horizon manipulation behaviors that occur within a few trading days.

Regarding investor sentiment, we recognize that sentiment activity during a month may partly reflect market reactions after price increases, rather than a purely ex ante driver of manipulation. For this reason, the sentiment variables are not interpreted as causal leading indicators. Instead, they are treated as observable public opinion dynamics associated with manipulation-related risk within the same firm–month risk window. This interpretation is consistent with the screening-oriented goal of the study, where the model captures predictive associations among disclosure, sentiment, and trading signals rather than establishing a causal sequence.

In the revised manuscript, we clarified these limitations in the Conclusion. We now state that the model primarily captures statistical associations rather than directly identifying specific manipulative actions, and that future research could incorporate higher-frequency trading records and event-level annotations to better capture short-horizon manipulation patterns.

**Amendments in the Manuscript:**

In Section 4.2.1, we clarified the retrospective nature of the labeling strategy:

The sanction records are used ex post to define supervised learning labels and are not used as explanatory variables in model training. The model is therefore interpreted as learning observable public-data patterns associated with retrospectively identified violation months and producing periodic manipulation-risk scores. **(Page 19, Section 4.2.1, lines 431-433)**

In the Conclusion, we expanded the limitation discussion as follows:

Despite these findings, the current model has several limitations. First, constrained by public data availability and labels constructed from ex post regulatory penalty documents, the model does not incorporate transaction-level order or trade information and therefore primarily captures statistical associations rather than directly identifying specific manipulative actions. Accordingly, the model output should be interpreted as a probabilistic risk score for candidate review and prioritization, and should be considered together with regulatory judgment and contextual verification. Legitimate information shocks, market-wide events, or sector-level fluctuations may also generate abnormal returns, sentiment bursts, and trading fluctuations, thereby increasing false positive risk in screening applications. Second, although the model exhibits overall stability within the current sample, its adaptability to extreme market conditions, emerging manipulation patterns, undetected manipulation cases, and non-Chinese market settings warrants further examination. Future research could extend this framework by incorporating higher-frequency trading records, event-level annotations, and richer investor-structure information. Cross-market evidence would also help assess the generalizability of the framework beyond China’s A-share market. **(Page 33, Section 5, lines 704–714)**

**Comment 23:**

*The authors base their study on a random 80/20 split of the sample and five repetitions (301–302). However, when working with panel data at the firm-month level, random splitting creates data leakage: observations from the same firm in different months appear in both the training and test samples. A firm-level split or a time split is necessary to eliminate this effect.*

*The training and test samples are split randomly (80/20), without regard to the time structure. It would be more methodologically sound to use the same periods as the training sample (2015–2021 = 70% of observations and 2022–2023 (30% of observations) or to split them as proposed 80%/20%, but in chronological order.*

**Response 23:**

Thank you for this important methodological comment. We agree that random splitting may allow observations from the same firm and different months to appear in both training and test samples, and may therefore affect the assessment of temporal generalizability.

In the revised manuscript, we have clarified the role of the repeated stratified random split results and added a chronological validation. Given the uneven distribution of positive observations across calendar years, the repeated random split results are retained to provide a comparable average-performance benchmark across models, while the chronological validation is added to address temporal generalizability under the panel structure. To reduce the influence of a particular random partition, all random split experiments are repeated using five random seeds, and model performance is now reported as mean ± standard deviation. The same set of random partitions is used across models to ensure comparability.

To address the reviewer’s concern regarding temporal structure, we further added a chronological validation in Section 4.3.3. In this additional test, the models are trained on observations from 2015 to 2020, validated on observations from 2021, and evaluated on observations from 2022 to 2023. Under this setting, all test observations occur after the training and validation periods, thereby preserving the calendar order of the sample.

The temporal validation results show that the BERT + MIL + MLP fusion model continues to perform best overall. It achieves an AUC of 0.8903, a PR-AUC of 0.7096, and the highest Recall@20% of 0.7273. These results indicate that the proposed multi-source fusion framework retains effective risk ranking ability when evaluated on later market periods. Therefore, the revised manuscript now reports both repeated random split results for average model comparison and chronological validation results for temporal generalizability.

**Amendments in the Manuscript:**

In Section 4.1, we revised the experimental design description to clarify the repeated random split setting and the additional chronological validation:

During training and testing, the main model comparison is based on stratified random splits, with 80% of the sample used for training and 20% for testing. To reduce the influence of a particular random partition, all random split experiments are repeated using five random seeds, and model performance is reported as mean ± standard deviation. The same set of random partitions is used across models to ensure comparability. Because the dataset is organized at the firm–month level, Section 4.3.3 further reports a chronological validation to assess temporal generalizability under the panel time structure. **(Page 15, Section 4.1, lines 337–341)**

We added Section 4.3.3 to report the chronological validation results:

4.3.3	Temporal Validation

To complement the repeated random split evaluation with a chronological design, this study further conducts a temporal validation. The models are trained on observations from 2015 to 2020, validated on observations from 2021, and evaluated on observations from 2022 to 2023. By preserving the calendar order of the sample, this design assesses the temporal generalizability of the risk assessment framework.

Table 8 reports the temporal validation results. The fusion model achieves the highest AUC and PR-AUC, with values of 0.8903 and 0.7096, respectively. It also obtains the highest Recall@20% of 0.7273, while its Recall@10% is equal to that of the Logit model. Although CatBoost attains a competitive AUC, its lower PR-AUC and Recall@K indicate weaker performance in prioritizing observations with elevated risk. The BERT + MIL model using only announcement text performs less favorably under the chronological split, suggesting that announcement semantics alone are more sensitive to temporal changes than the fused representation.

Overall, the temporal validation provides complementary evidence for the stability of the multi-source framework. While the repeated random split results evaluate average model performance under alternative sample partitions, the chronological split examines whether the learned risk patterns remain informative when the evaluation period moves forward in time. The results show that combining announcement semantics, public sentiment, and market trading signals preserves effective risk ranking ability in this temporal setting. Thus, the chronological validation should be interpreted as a complementary assessment of temporal generalizability, whereas the repeated random split results in Table 3 are reported as an average-performance benchmark for model comparison. **(Pages 29-30, Section 4.3.3, lines 663–680)**

We further added Table 8 to report the temporal validation results.

**Table 8. Temporal validation results.**

| **Model** | **Input** | **AUC** | **PR-AUC** | **Recall@10%** | **Recall@20%** |
| --- | --- | --- | --- | --- | --- |
| **Logit** | Numerical features | 0.8308 | 0.5767 | 0.3939 | 0.6364 |
| **CatBoost** | Numerical features | 0.8693 | 0.5452 | 0.2727 | 0.5758 |
| **BERT + MIL** | Announcement text | 0.7144 | 0.4261 | 0.2121 | 0.3333 |
| **BERT + MIL + MLP** | Announcement text + numerical features | 0.8903 | 0.7096 | 0.3939 | 0.7273 |

**Note:** The chronological split uses 2015–2020 as the training period, 2021 as the validation period, and 2022–2023 as the test period. Metrics are reported on the temporal test period.

**Comment 24:**

*The methodology is insufficiently described: it doesn't describe how the authors calculate CAR; which BERT model is used; which MLP model parameters are used; or how the texts were preprocessed. The lack of study details that could be included in an appendix makes the results difficult to reproduce and complicates their interpretation.*

**Response 24:**

Thank you for this important comment. We agree that the original manuscript did not provide sufficient methodological details regarding the construction of CAR, the specific BERT model, the MLP structure, and the preprocessing of announcement texts. In the revised manuscript, we have added concise implementation details in the relevant methodological subsections to improve reproducibility while keeping the main text focused.

First, in Section 3.2.3, we clarified the calculation of CAR and Vol. CAR is now defined as the within-stock cumulative sum of monthly abnormal returns, where the monthly abnormal return equals Mretwd minus the market return weighted by circulating market capitalization in the same month. We also clarified that Vol is computed as the rolling standard deviation of Mretwd over the most recent six observed months, including the current month, with at least three valid monthly observations required.

Second, in Section 4.1.2, we specified the BERT model and text preprocessing procedure. The revised manuscript clarifies that the implementation uses Chinese RoBERTa-wwm-ext, a general-purpose Chinese pretrained model with whole-word masking. Downloaded announcement PDFs are converted into cleaned plain text, and each announcement is represented using the [CLS] embedding.

Third, in Section 4.1.3, we added compact details of the fusion model architecture. Specifically, the structured numerical branch maps the 15 variables into a 64-dimensional representation, which is concatenated with the 768-dimensional BERT–MIL text representation and passed through a 128-dimensional hidden layer before the final sigmoid output. We also reported the optimizer and learning rate used in the fusion model training.

These revisions improve the reproducibility of the methodology while keeping the main text concise.

**Amendments in the Manuscript:**

CAR is computed as the within-stock cumulative sum of monthly abnormal returns, where monthly abnormal return equals Mretwd minus the market return weighted by circulating market capitalization in the same month. **(Page 12, Section 3.2.3, lines 280-282)**

…

In this study, volatility is computed as the rolling standard deviation of Mretwd over the most recent six observed months, including the current month, with at least three valid monthly observations required. **(Page 13, Section 3.2.3, lines 299-300)**

In the empirical implementation, we use Chinese RoBERTa-wwm-ext, a general-purpose Chinese pretrained model with whole-word masking, and extract the [CLS] embedding from each cleaned announcement text. Given the limited size of sanction-based labeled samples, the BERT encoder is frozen, while the MIL and fusion layers learn task-specific risk representations. (**Page 17, Section 4.1.2, lines 389-392)**

In the empirical implementation, the 15 structured variables are projected into a 64-dimensional ReLU-activated representation and concatenated with the 768-dimensional BERT–MIL text representation. The fused vector is passed through a 128-dimensional ReLU-activated hidden layer, followed by a sigmoid output layer, and the model is trained for 20 epochs using AdamW with a learning rate of 2e-3. **(Page 19, Section 4.1.3, lines 417-420)**

**Comment 25:**

*The paper doesn't mention excluding observation periods from the sample during which market factors could have influenced significant price changes. Are there plans to factor these into the model? Without this refinement, it's difficult to apply the model in practice, as the authors propose, as the number of false positives could increase significantly.*

**Response 25:**

Thank you for this important and practical comment. We agree that broader market factors, macroeconomic shocks, or sector-level events may generate significant price changes and public discussion that are not necessarily related to manipulation, and may therefore increase false positives in screening applications.

In the revised manuscript, we have clarified the practical interpretation of the model output. The proposed framework is not intended to directly establish manipulation or replace regulatory investigation. Instead, it generates probabilistic risk scores to support candidate review and prioritization under limited regulatory resources. Rather than mechanically excluding such periods, we interpret high-risk scores as candidate signals that require contextual verification, because broad market movements may coexist with firm-specific manipulation-related signals.

We also revised the Conclusion to explicitly acknowledge that legitimate information shocks, market-wide events, or sector-level fluctuations may generate abnormal returns, sentiment bursts, and trading fluctuations, thereby increasing false positive risk. In addition, the newly added screening utility analysis reports false positives and false discovery rates under Top 5%, Top 10%, and Top 20% review thresholds, providing a more concrete assessment of the trade-off between risk coverage and false positive burden.

**Amendments in the Manuscript:**

In Section 4.2.2, we added a screening utility analysis under capacity-based review thresholds. Table 5 reports the number of reviewed observations, true positives, false positives, precision, recall, and false discovery rate under Top 5%, Top 10%, and Top 20% review thresholds. This analysis provides a quantitative assessment of the trade-off between review burden, risk coverage, and false positive burden.

To complement the aggregate performance metrics, we further examine the screening utility of the final fusion model under capacity-based review thresholds. Test observations are ranked by risk scores assigned by the model, and the top 5%, 10%, and 20% are treated as candidate review sets. This analysis reports the number of firm–month observations selected for review, the number of true and false positives, precision, recall, and the false discovery rate. In this way, the model output is evaluated in terms of the practical trade-off among review burden, risk coverage, and false positive burden. **(Pages 24-25, Section 4.2.2, lines 549–553)**

**Table 5. Screening utility under capacity-based review thresholds**

| **Screening threshold** | **Reviewed observations** | **True positives** | **False positives** | **Precision** | **Recall** | **False discovery rate** |
| --- | --- | --- | --- | --- | --- | --- |
| **Top 5%** | 29 | 26.4 ± 0.9 | 2.6 ± 0.9 | 0.9103 ± 0.0308 | 0.2667 ± 0.0090 | 0.0897 ± 0.0308 |
| **Top 10%** | 59 | 45.0 ± 2.9 | 14.0 ± 2.9 | 0.7627 ± 0.0494 | 0.4545 ± 0.0294 | 0.2373 ± 0.0494 |
| **Top 20%** | 118 | 67.2 ± 1.6 | 50.8 ± 1.6 | 0.5695 ± 0.0139 | 0.6788 ± 0.0166 | 0.4305 ± 0.0139 |

**Note:** Except for reviewed observations, values are mean ± SD across five random seeds. Reviewed observations are firm–month observations. False discovery rate = false positives / reviewed observations.

Table 5 shows that the screening threshold materially affects the balance between risk coverage and false positive burden. A stricter Top 5% threshold yields high precision and a low false discovery rate, whereas expanding the review set to Top 20% substantially improves recall but also increases the false discovery rate. The Top 10% threshold provides an intermediate setting, capturing nearly half of the true risk observations while keeping the false discovery rate below 25%. These results provide a quantitative basis for review prioritization under limited regulatory resources using the risk scores assigned by the model. **(Page 25, Section 4.2.2, lines 558–563)**

In the Conclusion, we clarified the practical interpretation and limitations of the model output:

Accordingly, the model output should be interpreted as a probabilistic risk score for candidate review and prioritization, and should be considered together with regulatory judgment and contextual verification. Legitimate information shocks, market-wide events, or sector-level fluctuations may also generate abnormal returns, sentiment bursts, and trading fluctuations, thereby increasing false positive risk in screening applications. Second, although the model exhibits overall stability within the current sample, its adaptability to extreme market conditions, emerging manipulation patterns, undetected manipulation cases, and non-Chinese market settings warrants further examination. Future research could extend this framework by incorporating higher-frequency trading records, event-level annotations, and richer investor-structure information. Cross-market evidence would also help assess the generalizability of the framework beyond China’s A-share market. **(Page 31, Section 5, lines 707–714)**

**Comment 26:**

*The matching method (1:5, nearest-neighbor by month, industry, and market capitalization) is described briefly. The authors do not provide diagnostics for the quality of the matching: a covariate balancing table or a test for statistical equivalence of the groups after matching. Without these, it is impossible to assess whether systematic selection bias has been eliminated.*

**Response 26:**

Thank you for this important methodological comment. We agree that the original manuscript described the matching procedure too briefly and did not provide sufficient diagnostic evidence for assessing the quality of the matched sample. We also agree that, without such diagnostics, readers cannot adequately evaluate whether the violating and matched non-violating observations are comparable in terms of the observed matching covariates.

In the revised manuscript, we have expanded the description of the matching procedure and added covariate balance diagnostics in Panel B of Table 2. Specifically, each violating firm–month observation is matched with five non-violating firm–month observations using nearest-neighbor matching within the same month and industry, with matching distance calculated based on log market capitalization. Since month and industry are exactly matched by construction, the balance diagnostics focus on the size-related covariates. Panel B reports group means, standardized mean differences, and mean-difference tests for log market capitalization and raw market capitalization.

The results show that the absolute standardized mean differences are 0.0320 for log market capitalization and 0.0206 for raw market capitalization, both below the conventional 0.1 threshold. The corresponding mean-difference tests are also statistically insignificant. These results indicate satisfactory balance between the violating and matched non-violating observations in the observed size-related matching covariates.

At the same time, we have revised the wording cautiously. We do not claim that the matching procedure eliminates all possible selection bias, especially bias arising from unobserved factors. Instead, we state that the procedure improves sample comparability with respect to the observable matching dimensions used in this study.

**Amendments in the Manuscript:**

To ensure sample comparability, each violating firm–month observation is matched with five non-violating firm–month observations using nearest-neighbor matching within the same month and industry. Industry categories follow the CSRC classification standards, and nearest-neighbor distance is calculated based on log market capitalization. The 1:5 ratio is adopted to retain sufficient comparable control observations while keeping class imbalance at a manageable level for the supervised risk assessment task. Matching quality is assessed through covariate balance diagnostics at the matched-sample construction stage. Since month and industry are exactly matched by construction, the diagnostics focus on firm size. As shown in Panel B of Table 2, the absolute standardized mean differences are 0.0320 for log market capitalization and 0.0206 for raw market capitalization, both below the conventional 0.1 threshold, indicating good balance on the observed size-related matching covariates. **(Pages 19-20, Section 4.2.1, lines 434-442)**

We also added Panel B to Table 2 to report covariate balance diagnostics for the matched sample:

**Panel B. Covariate balance diagnostics for matched-sample construction.**

| **Covariate** | **Violating mean** | **Matched control mean** | **SMD** | **p-value** |
| --- | --- | --- | --- | --- |
| **Log market capitalization** | 14.8456 | 14.8750 | -0.0320 | 0.4408 |
| **Market capitalization** | 4,646,140 | 4,783,079 | -0.0206 | 0.6124 |

**Note:** Panel B reports covariate balance diagnostics at the matched-sample construction stage. Matching is performed within the same month and industry, and the nearest-neighbor distance is calculated based on log market capitalization. Since month and industry are exactly matched by construction, the diagnostics focus on size-related covariates. SMD denotes standardized mean difference. The p-values test mean differences between violating and matched control observations.

**Comment 27:**

*A 1:5 offender-to-nonoffender ratio creates a class imbalance of approximately 16–17% of positive observations (according to Table 2, (383–388)). The authors mention PR-AUC as a metric for imbalanced classes, but do not describe the use of any resampling techniques (SMOTE, oversampling) or class weighting during model training. This omission is particularly critical for Logit and CatBoost.*

**Response 27:**

Thank you for this important comment. We agree that the original manuscript did not clearly describe how the class imbalance resulting from the 1:5 matched sample design was handled during model training, particularly for the Logit and CatBoost baselines.

In the revised manuscript, we have clarified the imbalance-aware training settings in Section 4.1.1. The reported experiments retain the matched firm–month sample composition and use a weighting-based strategy for the structured-feature baselines. Specifically, the Logit model is estimated with balanced class weights, while CatBoost uses a training-split scale_pos_weight, calculated as the ratio of negative to positive observations within each training set. Because the weight is computed within each training split, the imbalance adjustment is aligned with the repeated random-split design.

We have also refined the evaluation description in Section 4.1. Since the matched firm–month sample remains imbalanced and the objective of the study is screening-oriented risk prioritization, model performance is evaluated from two perspectives: overall discriminative ability and high-risk coverage. PR-AUC is used to emphasize predictive quality for the minority high-risk class, while Recall@10% and Recall@20% assess whether truly sanctioned observations are captured within the top-ranked review sets.

These revisions make the training and evaluation protocol clearer. The Logit and CatBoost comparisons now explicitly report their class-weighting settings, while the use of PR-AUC and Recall@K is tied to the imbalanced and screening-oriented nature of the task.

**Amendments in the Manuscript:**

Given the imbalanced and screening-oriented nature of the matched firm–month sample, model performance is assessed from two perspectives: overall discriminative ability and high-risk coverage. (**Page 15, Section 4.1, lines 343–345)**

In the implementation, missing numerical values are imputed using training-set medians. Logit is estimated with standardized inputs, L2 regularization, and balanced class weights, while CatBoost uses a fixed configuration with 500 iterations, depth = 4, learning rate = 0.03, Logloss, AUC, and a training-split scale_pos_weight. (**Page 17, Section 4.1.1, lines 376–379)**

**Comment 28:**

*The use of pre-trained BERT for Chinese requires clarification: which specific version is used (Google's Chinese BERT base, MacBERT, FinBERT-zh, or another)? (337–356) The financial domain differs significantly from the general-purpose corpora on which the standard BERT is trained. The lack of domain-specific fine-tuning (finetuning on financial texts) or its justification reduces the credibility of the results.*

**Response 28:**

Thank you for this important comment. We agree that the original manuscript did not clearly specify the exact Chinese pretrained language model used in the text module or the fine-tuning strategy, which could make the role of the BERT encoder ambiguous.

In the revised manuscript, we have clarified that the text encoder used in the main experiments is Chinese RoBERTa-wwm-ext, a general-purpose Chinese pretrained model with whole-word masking. The model is used to obtain contextual announcement-level representations, and the [CLS] embedding of each cleaned announcement text is extracted as the input representation for the MIL module.

We also clarified that the BERT encoder is frozen in the main experiments. This implementation choice is motivated by the limited size of sanction-based labeled samples. Directly fine-tuning a large pretrained encoder on a relatively small firm–month supervision task may increase overfitting risk and reduce training stability. Therefore, the pretrained encoder is used as a stable semantic representation layer, while task-specific learning is performed by the MIL and fusion layers.

This clarification helps readers accurately understand the role of BERT in our framework. BERT is used to encode announcement texts, and the final fusion model combines the resulting announcement-text representation with 15 structured variables covering disclosure characteristics, public sentiment, and market trading behavior. Thus, domain-relevant information is incorporated through both contextual text representations and supervised multi-source fusion.

**Amendments in the Manuscript:**

In the empirical implementation, we use Chinese RoBERTa-wwm-ext, a general-purpose Chinese pretrained model with whole-word masking, and extract the [CLS] embedding from each cleaned announcement text. Given the limited size of sanction-based labeled samples, the BERT encoder is frozen, while the MIL and fusion layers learn task-specific risk representations. (**Page 17, Section 4.1.2, lines 389-392)**

**Comment 29:**

*The architecture of the final fusion BERT+MIL+MLP is described at a conceptual level (358–375), but technical details are completely missing: the dimensionality of the hidden layers of the MLP, activation functions other than sigmoid, dropout parameters, optimization algorithm, learning rate, and number of epochs. Without these, reproducibility of the study is impossible.*

**Response 29:**

Thank you for this important and specific comment. We agree that the original description of the final BERT + MIL + MLP fusion model remained at a relatively conceptual level and did not provide enough implementation details for reproducibility.

In the revised manuscript, we have added a compact description of the implemented fusion architecture in Section 4.1.3. Specifically, the 15 structured numerical variables are projected into a 64-dimensional representation with ReLU activation and then concatenated with the 768-dimensional BERT–MIL text representation. The fused vector is passed through a 128-dimensional ReLU-activated hidden layer before the final sigmoid output layer. We have also specified the main training configuration: the model is trained for 20 epochs using AdamW with a learning rate of 2e-3.

To avoid ambiguity, the revised description reports the actual implemented MLP configuration rather than a general architectural description. The implemented fusion MLP is fully specified by the feed-forward layers described above, with no additional dropout hyperparameter to tune. These revisions make the model architecture and training procedure more transparent while keeping the methodological description concise.

**Amendments in the Manuscript:**

In the empirical implementation, the 15 structured variables are projected into a 64-dimensional ReLU-activated representation and concatenated with the 768-dimensional BERT–MIL text representation. The fused vector is passed through a 128-dimensional ReLU-activated hidden layer, followed by a sigmoid output layer, and the model is trained for 20 epochs using AdamW with a learning rate of 2e-3. We then systematically compare this fusion model with the Logit baseline, the CatBoost classifier, and the text-only BERT + MIL model, evaluating the practical value of multi-source information fusion in manipulation risk assessment in terms of predictive performance and stability. **(Page 19, Section 4.1.3, lines 417-422)**

**Comment 30:**

*The explanation for the concentration of violations in 2017–2020 by the "retrospective labeling" mechanism (lines 414–419) is logically correct, but raises an unsolved problem: is there a survivorship bias—a systematic underrepresentation of earlier violations for which the statute of limitations expired before 2022? The authors do not discuss this source of bias.*

**Response 30:**

Thank you for this important and thoughtful comment. We agree that the retrospective labeling mechanism may introduce survivorship or sample-coverage bias. Earlier manipulation activities that were not publicly sanctioned within the 2022–2024 enforcement window may be underrepresented in the sample.

In the revised manuscript, we have provided a more cautious interpretation of Figure 2 and Table 2. The concentration of violating observations between 2017 and 2020 should not be interpreted as the market-wide incidence of manipulation. It reflects the temporal coverage of sanctioned cases in an enforcement-based sample. Such sample-coverage limitations are inherent to publicly available regulatory penalty data, which are affected by detection lag, disclosure lag, and regulatory enforcement windows.

This sample-coverage issue does not change the screening-oriented objective of the study. This study does not aim to recover the complete population of all manipulation activities in China’s A-share market. Instead, it uses CSRC-sanctioned manipulation cases as a reliable publicly available source of supervised labels and learns the multi-source risk patterns associated with regulatory-recognized and sanctioned manipulation periods. The revised text therefore avoids treating the sample pattern as evidence of the true market-wide frequency of manipulation.

**Amendments in the Manuscript:**

We begin by examining the annual distribution of violating firm–month observations, as presented in Figure 2 and Table 2. It is important to clarify that the reported violation counts are measured at the firm–month level rather than by the number of manipulation cases. The sample is constructed based on administrative penalty announcements issued by the CSRC during 2022–2024. Using the violation intervals disclosed in the penalty documents, manipulation behaviors are retrospectively mapped to the months in which they occurred. Consequently, a single case spanning multiple calendar months corresponds to multiple violating observations. From a temporal perspective, violating observations are relatively concentrated between 2017 and 2020. This concentration reflects the enforcement-based sample design rather than the market-wide incidence of manipulation. Sanctioned cases often involve persistent, stage-like conduct, with violation intervals spanning multiple months. Since the sample is defined by CSRC penalty announcements issued during 2022–2024 and backdated to the disclosed violation periods, the observed distribution represents the temporal coverage of sanctioned cases within this sample. **(Page 21, Section 4.2.2, lines 469-478)**

**Comment 31:**

*The use of Cohen's d to characterize the separating power of variables (422–435) is methodologically acceptable; however, the authors interpret negative d values for ann_sent as evidence that "manipulative firms use a less positive tone" (pp. 204–208), without conducting statistical tests for the significance of the differences. P-values or confidence intervals for each Cohen's d should be included.*

**Response 31:**

Thank you for this important comment. We agree that the original interpretation of the negative Cohen’s d value for ann_sent was too direct and was not sufficiently supported by uncertainty estimates. Although Cohen’s d is useful for describing standardized between-group differences, the original figure did not show whether these differences were statistically distinguishable from zero.

In the revised manuscript, we have updated Figure 3 to report Cohen’s d together with bootstrap 95% confidence intervals for all 15 numerical variables. This allows readers to assess not only the direction and magnitude of each standardized mean difference, but also the statistical uncertainty around the estimate. We have also revised the accompanying text to state explicitly that confidence intervals excluding zero indicate statistically distinguishable group differences.

Importantly, we have softened the interpretation of ann_sent and ann_fog. Instead of claiming that manipulative firms “use a less positive tone,” we now describe the result as lower standardized means among violating observations and interpret it as evidence that disclosure-related signals differ across risk states. We further emphasize that these disclosure and sentiment signals may become more informative when evaluated jointly with trading features, rather than acting as standalone predictors. This revision avoids over-interpreting the descriptive Cohen’s d results as a direct behavioral or causal mechanism.

**Amendments in the Manuscript:**

**Fig 3. Standardized mean differences with 95% confidence intervals.**

To characterize the direction, magnitude, and statistical uncertainty of group differences, Figure 3 reports standardized mean differences measured by Cohen’s d with bootstrap 95% confidence intervals. This metric removes the influence of scale and allows the 15 variables to be compared on a common basis. Positive values indicate higher feature means among violating observations, whereas negative values indicate lower means. Confidence intervals that exclude zero indicate statistically distinguishable differences between the two groups.

As shown in Figure 3, market trading variables provide the strongest univariate contrasts. CAR has the largest positive standardized difference, followed by MCCR, Mretwd, and Vol, indicating that violating observations are more strongly associated with abnormal return movements, market value changes, and price fluctuations. Announcement and sentiment variables show more moderate marginal differences. The negative differences in ann_sent and ann_fog indicate lower standardized means among violating observations, suggesting that disclosure-related signals differ across risk states even when their univariate magnitudes are smaller. This pattern implies that these disclosure and sentiment signals may emerge more prominently through interactions with trading features rather than as standalone predictors. This interpretation is further supported by the performance gains observed in the fusion model and by the SHapley Additive exPlanations (SHAP) results discussed later. **(Pages 21-22, Section 4.2.2, lines 481-494)**

**Comment 32:**

*Table 3 is the key result of the article. However, the authors (448–479) do not conduct statistical significance tests of the differences between the models (e.g., the Deming–McNemar test for AUC or bootstrap tests). The gap between BERT+MIL and the final model in AUC (0.8680 vs. 0.8767) is small, and without a significance test, it cannot be concluded that the increase is not due to chance.*

**Response 32:**

Thank you for this important comment. We agree that Table 3 is a key empirical result of the manuscript and that the original version did not provide sufficient statistical evidence to support the differences between models. We also agree that the comparison between the pure-text BERT + MIL model and the final fusion model requires particular caution, because the performance gap may appear modest if only point estimates are considered.

In the revised manuscript, all model comparisons are reported consistently under the same five-seed repeated random-split protocol. Table 3 now presents model performance as mean ± SD, and Table 4 reports paired significance tests based on the same repeated partitions. Specifically, for each metric and each random seed, we calculate the performance difference between the final BERT + MIL + MLP fusion model and each comparison model. We then conduct seed-level paired t-tests on these paired differences. This design allows us to examine whether the observed improvements are statistically supported under matched experimental conditions.

The revised results show that the final fusion model achieves higher average performance than the BERT + MIL model. For the specific comparison emphasized by the reviewer, the AUC difference between the final fusion model and the pure-text BERT + MIL model is statistically significant in the paired test. The paired test also shows significant improvements in PR-AUC and Recall@20%. However, the improvement in Recall@10% is not statistically significant. We have therefore revised the interpretation to avoid claiming uniform improvement across all metrics. Instead, we now state more cautiously that multi-source fusion mainly improves overall ranking quality and broader high-risk coverage, while its additional contribution is less pronounced within the most restrictive Top 10% screening threshold.

**Amendments in the Manuscript:**

**Table 3. Comparative Evaluation of Model Performance.**

| **Model** | **Input** | **AUC** | **PR-AUC** | **Recall@10%** | **Recall@20%** |
| --- | --- | --- | --- | --- | --- |
| **Logit** | Numerical features | 0.6984±0.0268 | 0.3549±0.0329 | 0.2646±0.0374 | 0.4202±0.0332 |
| **CatBoost** | Numerical features | 0.7768±0.0389 | 0.5130±0.0562 | 0.3394±0.0382 | 0.4949±0.0647 |
| **BERT + MIL** | Announcement text | 0.8296±0.0286 | 0.6464±0.0464 | 0.4384±0.0444 | 0.6323±0.0402 |
| **BERT + MIL + MLP** | Announcement text + numerical features | 0.8811±0.0063 | 0.6943±0.0158 | 0.4545±0.0294 | 0.6788±0.0166 |

**Note:** Values are reported as mean ± SD across five random seeds. The same random partitions are used across models.

**Table 4. Paired significance tests based on repeated random seeds.**

| **Comparison** | **AUC diff.** | **AUC p-value** | **PR-AUC diff.** | **PR-AUC p-value** | **Recall@10% diff.** | **Recall@10% p-value** | **Recall@20% diff.** | **Recall@20% p-value** |
| --- | --- | --- | --- | --- | --- | --- | --- | --- |
| **Fusion vs Logit** | 0.1827 | <0.001 | 0.3394 | <0.001 | 0.1899 | 0.0026 | 0.2586 | <0.001 |
| **Fusion vs CatBoost** | 0.1043 | 0.0028 | 0.1813 | 0.0018 | 0.1152 | 0.0079 | 0.1838 | 0.0023 |
| **Fusion vs BERT+MIL** | 0.0515 | 0.0091 | 0.0478 | 0.0388 | 0.0162 | 0.4382 | 0.0465 | 0.0303 |

**Note:** This table reports seed-level paired t-tests based on the same five repeated random-seed experiments used in Table 3. For each metric, the difference is calculated as the performance of the final fusion model minus that of the comparison model under the same seed. The reported diff. is the average paired difference, and the p-value tests whether the mean paired difference differs from zero.

In Section 4.2.2, we revised the interpretation of the model comparison results as follows:

To reduce dependence on a particular random partition, all random split experiments are repeated using five random seeds, and Table 3 reports model performance as mean ± standard deviation. The same random partitions are used across models, which makes the model comparisons paired at the seed level. Rather than relying only on point estimates, Table 4 further examines whether the performance differences between the final fusion model and each comparison model are statistically supported under this paired design. The relatively small standard deviations of the fusion model, particularly for AUC, PR-AUC, and Recall@20%, indicate that its performance is not highly sensitive to a particular random split. **(Page 23, Section 4.2.2, lines 516-521)**

……

Building on this foundation, the final BERT + MIL + MLP model combines the announcement-text representation with the 15 structured numerical indicators and achieves the highest average performance across all four metrics. The paired tests provide statistical support for the improvements over the two structured-feature models, suggesting that these gains are unlikely to be driven solely by random split variation. Relative to the pure-text BERT + MIL model, the evidence is more nuanced. The fusion model shows statistically significant improvements in AUC, PR-AUC, and Recall@20%, whereas the improvement in Recall@10% is not statistically significant. This pattern suggests that multi-source fusion mainly improves overall ranking quality and broader high-risk coverage, while its additional contribution is less pronounced within the most restrictive Top 10% screening threshold. **(Page 24, Section 4.2.2, lines 536-543)**

**Comment 33:**

*The authors report repeating the experiment five times and averaging the results (449–450), but do not provide standard deviations of the metrics across iterations. Without measures of variability, it is impossible to assess the stability of the results.*

**Response 33:**

Thank you for this important comment. We agree that reporting only averaged results across the five repeated experiments was insufficient for evaluating the stability of model performance.

In the revised manuscript, we have updated Table 3 to report all evaluation metrics as mean ± standard deviation across the five repeated random-seed experiments. The same random partitions are used across models to ensure that the comparison is based on matched experimental conditions. This revision enables readers to assess not only the average performance of each model, but also the variability of each metric across repeated iterations.

We have also revised the discussion in Section 4.2.2 to explicitly connect the reported standard deviations with result stability. The final BERT + MIL + MLP fusion model shows relatively small standard deviations, particularly for AUC, PR-AUC, and Recall@20%, suggesting that its performance is not highly sensitive to a particular random split. These revisions provide a clearer basis for assessing the stability and reliability of the model comparison results.

**Amendments in the Manuscript:**

**Table 3. Comparative Evaluation of Model Performance.**

| **Model** | **Input** | **AUC** | **PR-AUC** | **Recall@10%** | **Recall@20%** |
| --- | --- | --- | --- | --- | --- |
| **Logit** | Numerical features | 0.6984±0.0268 | 0.3549±0.0329 | 0.2646±0.0374 | 0.4202±0.0332 |
| **CatBoost** | Numerical features | 0.7768±0.0389 | 0.5130±0.0562 | 0.3394±0.0382 | 0.4949±0.0647 |
| **BERT + MIL** | Announcement text | 0.8296±0.0286 | 0.6464±0.0464 | 0.4384±0.0444 | 0.6323±0.0402 |
| **BERT + MIL + MLP** | Announcement text + numerical features | 0.8811±0.0063 | 0.6943±0.0158 | 0.4545±0.0294 | 0.6788±0.0166 |

**Note:** Values are reported as mean ± SD across five random seeds. The same random partitions are used across models.

In Section 4.2.2, we revised the relevant discussion as follows:

To reduce dependence on a particular random partition, all random split experiments are repeated using five random seeds, and Table 3 reports model performance as mean ± standard deviation. The same random partitions are used across models, which makes the model comparisons paired at the seed level. Rather than relying only on point estimates, Table 4 further examines whether the performance differences between the final fusion model and each comparison model are statistically supported under this paired design. The relatively small standard deviations of the fusion model, particularly for AUC, PR-AUC, and Recall@20%, indicate that its performance is not highly sensitive to a particular random split. **(Page 23, Section 4.2.2, lines 516–521)**

**Comment 34:**

*SHAP analysis is only performed for the final model (485–512). To make the interpretation more convincing, it would be worth comparing the feature importance between CatBoost and BERT+MIL+MLP to demonstrate how the addition of text features changes the contribution of trading variables.*

**Response 34:**

Thank you for this valuable suggestion. We agree that interpreting only the final fusion model was insufficient for showing how feature contributions change after incorporating announcement-text representations. In the revised manuscript, we have added a comparative SHAP analysis between CatBoost and the final BERT + MIL + MLP fusion model.

Specifically, we compare normalized SHAP importance shares between CatBoost and the structured numerical branch of the fusion model. The comparison is restricted to the 15 shared structured variables, because the BERT textual representation is encoded as a high-dimensional semantic vector and cannot be directly decomposed into the same financially interpretable variables as the structured features. This design allows us to examine whether the relative contribution pattern of structured variables changes after the model incorporates announcement-text representations. Therefore, this comparison is intended to examine the redistribution of importance among shared structured variables, rather than to interpret individual BERT semantic dimensions.

The revised results show that market trading features remain the largest structured feature group in both models, but their normalized importance share decreases from 0.504 in CatBoost to 0.469 in the fusion model. Announcement features show the largest relative increase, rising from 0.178 to 0.307, while public sentiment features decrease from 0.318 to 0.224. These results suggest that the fusion model does not simply reproduce the CatBoost importance structure. Instead, after announcement-text representations are incorporated, structured disclosure variables gain relative importance, whereas part of the contribution previously assigned to sentiment and trading variables is absorbed into a more balanced multi-source representation.

**Amendments in the Manuscript:**

To further examine whether the fusion model changes the contribution pattern of structured variables, we compare normalized SHAP importance shares between CatBoost and the structured numerical branch of the fusion model. The comparison is restricted to the 15 shared structured variables, because the BERT textual representation cannot be directly decomposed into financially interpretable variables. Market trading features remain the largest structured feature group in both models, although their normalized share decreases from 0.504 in CatBoost to 0.469 in the fusion model. Announcement features show the largest relative increase, rising from 0.178 to 0.307, while public sentiment features decrease from 0.318 to 0.224. This redistribution suggests that the fusion model does not simply reproduce the CatBoost importance structure. Instead, after announcement-text representations are incorporated, structured disclosure variables gain relative importance, whereas part of the contribution previously assigned to sentiment and trading variables is absorbed into a more balanced multi-source representation. **(Page 26, Section 4.2.2, lines 584–593)**

**Comment 35:**

*The dominance of CAR (cumulative abnormal returns) in the SHAP rating (487–489) raises a fundamental methodological question: CAR is effectively the outcome of manipulation, not its leading indicator. If the model relies primarily on this indicator, it essentially detects manipulation after the fact, rather than predicting risk. This contradicts the stated goal of "front-end risk screening" (32–33, 528–529) and requires serious discussion.*

**Response 35:**

Thank you for this important and rigorous comment. We agree that the high importance of cumulative abnormal returns (CAR) raises a fundamental methodological issue. CAR directly reflects realized abnormal price movements and therefore should not be interpreted as a purely leading indicator or as transaction-level evidence of manipulative behavior. We also agree that, if the model relied primarily or exclusively on CAR, the framework would risk being reduced to the detection of price anomalies after the fact, which would be inconsistent with a screening-oriented risk assessment objective.

In the revised manuscript, we have therefore clarified the scope of the study and refined the interpretation of the model output. The objective of the model is not to identify specific manipulative transactions or to provide transaction-level proof of manipulation. Rather, the study constructs a firm-month risk assessment framework based on publicly observable multi-source signals. In this setting, CAR is treated as a market response variable that captures the price manifestation of elevated risk within the observation period, not as a stand-alone leading indicator or direct evidence of manipulation. We have revised the relevant discussion to make clear that the model output should be interpreted as a probabilistic risk score for regulatory prioritization, rather than as transaction-level detection of manipulation.

We have also revised the SHAP interpretation accordingly. In the revised text, the dominance of CAR is no longer presented as evidence that the model has identified manipulation itself. Instead, we interpret CAR as indicating that abnormal return deviations provide substantial information about the market-level manifestation of risk. At the same time, we emphasize that the explanatory pattern is not confined to CAR. Announcement volume, announcement textual complexity, and sentiment-related variables also rank among the important features, suggesting that disclosure behavior and investor attention provide additional information beyond market trading variables.

To directly examine whether the model is driven mainly by CAR, we added a feature ablation analysis in Section 4.3.2. In this analysis, CAR is removed from the structured numerical feature set, while the sample, labels, announcement texts, remaining structured variables, and fusion model architecture remain unchanged. The results show that removing CAR reduces model performance: the average AUC decreases from 0.8811 to 0.8370, PR-AUC decreases from 0.6943 to 0.5743, Recall@10% decreases from 0.4545 to 0.3818, and Recall@20% decreases from 0.6788 to 0.6121. This indicates that CAR contains important information for manipulation risk assessment.

However, the CAR exclusion specification still retains meaningful risk ranking capacity, with an AUC of 0.8370 and a Recall@20% of 0.6121. This result indicates that announcement semantics, disclosure characteristics, sentiment variables, and the remaining trading indicators continue to provide useful screening information after CAR is removed. Therefore, the revised interpretation is more cautious: CAR materially improves model performance as an important market response signal, but the model’s risk assessment output is not reducible to accumulated abnormal returns alone. Accordingly, we have revised the SHAP discussion, clarified the interpretation of CAR and the model output, and added Section 4.3.2 and Table 7 to report the CAR ablation analysis.

**Amendments in the Manuscript:**

In the SHAP interpretation section, we revised the discussion of CAR to avoid interpreting it as direct evidence of manipulation:

As shown in Figure 5, the CAR variable ranks first in the global importance results, indicating that abnormal return deviations provide the strongest structured signal for the model’s risk scoring. This finding is consistent with the role of price responses in market manipulation cases, where abnormal returns often represent an observable market-level manifestation of risk. Announcement volume (ann_cnt) and announcement textual complexity (ann_fog) rank immediately after CAR, suggesting that formal disclosure behavior provides substantial information in addition to market trading variables. Sentiment-related indicators, including posting volume (post_cnt) and the daily variability of posting activity (post_cnt_std_day), also appear among the important features, suggesting that the model assigns greater importance to the intensity and temporal clustering of investor attention than to passive information exposure. These results indicate that the fusion model captures not only abnormal price movements, but also variation in corporate disclosure behavior and the concentration of investor attention. **(Page 26, Section 4.2.2, lines 575–583)**

We also added Section 4.3.2 to examine whether the fusion model is mainly driven by CAR:

4.3.2	Feature Ablation of CAR

The SHAP analysis identifies cumulative abnormal returns (CAR) as the most influential structured trading feature. To assess the incremental role of this price response variable, we conduct a feature ablation by removing CAR from the structured numerical feature set. The sample, labels, announcement texts, remaining structured variables, and model architecture are kept unchanged.

Table 7 reports the results of the CAR ablation analysis. After CAR is removed, model performance declines across all evaluation metrics. The average AUC decreases from 0.8811 to 0.8370, PR-AUC decreases from 0.6943 to 0.5743, Recall@10% decreases from 0.4545 to 0.3818, and Recall@20% decreases from 0.6788 to 0.6121. These results indicate that CAR provides substantial information for manipulation risk assessment and constitutes an important component of the market trading feature group.

Despite this decline, the CAR exclusion specification continues to show risk ranking capacity, with an AUC of 0.8370 and a Recall@20% of 0.6121. This suggests that announcement semantics, disclosure characteristics, sentiment variables, and the remaining trading indicators still contain useful screening information after CAR is removed. Therefore, the full model should be interpreted as a multi-source risk assessment framework in which abnormal return information improves model performance, while textual, sentiment, and other trading signals also contribute to the final risk assessment output. **(Pages 28-29, Section 4.3.2, lines 643–657)**

We further added Table 7 to report the feature ablation results excluding CAR.

**Table 7. Feature ablation analysis excluding CAR.**

| **Specification** | **Feature setting** | **AUC** | **PR-AUC** | **Recall@10%** | **Recall@20%** |
| --- | --- | --- | --- | --- | --- |
| **Main specification** | Full feature set | 0.8811±0.0063 | 0.6943±0.0158 | 0.4545±0.0294 | 0.6788±0.0166 |
| **CAR excluded specification** | CAR removed from structured numerical features | 0.8370±0.0236 | 0.5743±0.0591 | 0.3818±0.0522 | 0.6121±0.0382 |

**Note:** The main specification is repeated from Table 3 for reference. In the CAR exclusion specification, cumulative abnormal returns are removed from the structured numerical feature set. The sample, labels, announcement texts, remaining structured variables, and fusion model architecture remain unchanged.

**Comment 36:**

*There is no discussion in the article.*

*In conclusion, the finding that "manipulation manifests itself through the coordinated evolution of signals" is a descriptive observation, not a rigorously proven assertion. The study does not test causal relationships between disclosure, sentiment, and trading; it establishes predictive associations. The authors should clearly distinguish between these concepts.*

**Response 36:**

Thank you for this rigorous and constructive comment. We appreciate the reviewer’s concern regarding the distinction between predictive association and causal interpretation. The purpose of this study is not to test causal relationships among disclosure behavior, sentiment diffusion, and market trading responses. Rather, our aim is to examine whether these publicly observable signals jointly provide useful information for firm–month manipulation risk assessment.

In the revised manuscript, we have refined the wording of the Conclusion to make this scope clearer. The original intention was to describe a multi-source risk pattern reflected in the model results, not to claim a causal mechanism. To avoid possible overinterpretation, we now state that observations with elevated risk tend to exhibit joint deviations across disclosure behavior, sentiment diffusion, and market responses, and that such observations are assigned higher risk scores by the model. This revision preserves the substantive empirical finding while more accurately characterizing it as a predictive association and risk representation pattern.

We also revised the concluding interpretation to emphasize that manipulation risk is represented through a structured configuration of observable signals. This wording is consistent with the screening-oriented objective of the study: the model is designed to support risk ranking and candidate review, rather than to establish causal relationships among information disclosure, sentiment, and trading behavior.

**Amendments in the Manuscript:**

The findings suggest that manipulation risk is not fully reflected by a single anomalous factor; rather, observations with elevated risk tend to exhibit joint deviations across disclosure behavior, sentiment diffusion, and market responses. Firm–month observations showing such concurrent deviations are assigned higher risk scores by the model. The model demonstrates stable performance and screening utility in prioritizing observations with elevated risk, indicating that multi-source fusion can improve risk ranking when individual indicators are noisy or incomplete. Accordingly, manipulation risk is better represented as a structured configuration of observable signals rather than as a simple aggregation of isolated indicators. **(Pages 30-31, Section 5, lines 688–694)**

**Comment 37:**

*The authors acknowledge (531–532) that the model "captures statistical associations rather than causal mechanisms." This is a significant limitation, but its discussion takes only one sentence. It needs to be expanded: what specific conclusions for regulators might be erroneous if the associations are not causal?*

*(line 70): "**the CSRC" is written twice—a typo that must be corrected.*

**Response 37:**

Thank you for this important comment. We agree that the distinction between statistical association and causal mechanism is a substantive limitation and should be discussed more explicitly.

In the revised manuscript, we have expanded the limitation discussion in the Conclusion. We now clarify that, constrained by public data availability and labels constructed from ex post regulatory penalty documents, the model primarily captures statistical associations rather than directly identifying specific manipulative actions. Therefore, the model output should be interpreted as a probabilistic risk score for candidate review and prioritization, and should be considered together with regulatory judgment and contextual verification.

We have also added a more concrete discussion of the potential regulatory misinterpretation. If the associations captured by the model were treated as causal or evidentiary conclusions, regulators might incorrectly attribute abnormal returns, sentiment bursts, or disclosure-related signals to manipulation, even when these patterns arise from legitimate information shocks, market-wide events, or sector-level fluctuations. This could increase false positive risk in screening applications. The revised manuscript therefore emphasizes that the model is intended to support risk-based prioritization, not to provide stand-alone causal evidence or enforcement-level proof of manipulation.

The typographical error in line 70, where “the CSRC” was repeated, has also been corrected.

**Amendments in the Manuscript:**

Despite these findings, the current model has several limitations. First, constrained by public data availability and labels constructed from ex post regulatory penalty documents, the model does not incorporate transaction-level order or trade information and therefore primarily captures statistical associations rather than directly identifying specific manipulative actions. Accordingly, the model output should be interpreted as a probabilistic risk score for candidate review and prioritization, and should be considered together with regulatory judgment and contextual verification. Legitimate information shocks, market-wide events, or sector-level fluctuations may also generate abnormal returns, sentiment bursts, and trading fluctuations, thereby increasing false positive risk in screening applications. **(Page 31, Section 5, lines 704–710)**

We also corrected the duplicated “the CSRC” in the literature review. The revised sentence now reads:

In the Chinese regulatory context, Liu et al. used administrative penalty cases disclosed by the CSRC (China Securities Regulatory Commission) to develop machine learning models and showed that trading intensity, price volatility, and transaction-structure variables have discriminative value for identifying manipulation [5]. **(Page 4, Section 2.1, lines 78–81)**

**Comment 38:**

*The claim that the model is applicable to "risk stratification" and "tiered supervision" (524–530) is not supported by an analysis of threshold calibration or an assessment of the probability of false positives under real-world screening volumes. Practical applicability remains declarative.*

**Response 38:**

Thank you for this important comment. We agree that the original manuscript did not provide sufficient evidence to support the practical applicability of the model for risk stratification and tiered screening. In particular, the previous version reported aggregate metrics such as AUC, PR-AUC, and Recall@K, but did not explicitly quantify the review burden and the risk of false positives under different screening volumes.

In the revised manuscript, we have added a screening utility analysis under capacity-based review thresholds. Specifically, test observations are ranked by the risk scores assigned by the final fusion model, and the top 5%, 10%, and 20% are treated as candidate review sets. These thresholds approximate different review capacity scenarios and are used to evaluate screening utility rather than to define enforcement thresholds. For each threshold, we report the number of firm–month observations selected for review, the number of true and false positives, precision, recall, and the false discovery rate. This analysis allows the practical trade-off between risk coverage and review burden to be evaluated more directly.

The results show that different review thresholds imply different regulatory screening trade-offs. A stricter Top 5% threshold yields high precision and a low false discovery rate, whereas the Top 20% threshold improves recall but also increases the false discovery rate. The Top 10% threshold provides an intermediate setting, capturing nearly half of the true risk observations while keeping the false discovery rate below 25%. These results provide a quantitative basis for using the model’s risk scores to support review prioritization under limited regulatory resources.

We have also clarified the practical interpretation of the model output. Rather than presenting the model as a direct enforcement tool, we interpret it as a screening-oriented risk prioritization tool that helps regulators prioritize candidate firm–month observations under limited review resources.

**Amendments in the Manuscript:**

To complement the aggregate performance metrics, we further examine the screening utility of the final fusion model under capacity-based review thresholds. Test observations are ranked by risk scores assigned by the model, and the top 5%, 10%, and 20% are treated as candidate review sets. This analysis reports the number of firm–month observations selected for review, the number of true and false positives, precision, recall, and the false discovery rate. In this way, the model output is evaluated in terms of the practical trade-off among review burden, risk coverage, and false positive burden. **(Pages 24-25, Section 4.2.2, lines 549–553)**

**Table 5. Screening utility under capacity-based review thresholds**

| **Screening threshold** | **Reviewed observations** | **True positives** | **False positives** | **Precision** | **Recall** | **False discovery rate** |
| --- | --- | --- | --- | --- | --- | --- |
| **Top 5%** | 29 | 26.4 ± 0.9 | 2.6 ± 0.9 | 0.9103 ± 0.0308 | 0.2667 ± 0.0090 | 0.0897 ± 0.0308 |
| **Top 10%** | 59 | 45.0 ± 2.9 | 14.0 ± 2.9 | 0.7627 ± 0.0494 | 0.4545 ± 0.0294 | 0.2373 ± 0.0494 |
| **Top 20%** | 118 | 67.2 ± 1.6 | 50.8 ± 1.6 | 0.5695 ± 0.0139 | 0.6788 ± 0.0166 | 0.4305 ± 0.0139 |

**Note:** Except for reviewed observations, values are mean ± SD across five random seeds. Reviewed observations are firm–month observations. False discovery rate = false positives / reviewed observations.

Table 5 shows that the screening threshold materially affects the balance between risk coverage and false positive burden. A stricter Top 5% threshold yields high precision and a low false discovery rate, whereas expanding the review set to Top 20% substantially improves recall but also increases the false discovery rate. The Top 10% threshold provides an intermediate setting, capturing nearly half of the true risk observations while keeping the false discovery rate below 25%. These results provide a quantitative basis for review prioritization under limited regulatory resources using the risk scores assigned by the model. **(Page 25, Section 4.2.2, lines 558–563)**

**Comment 39:**

*In several places, the article uses the terms "manipulation risk" and "manipulation detection" interchangeably, although conceptually these are different tasks: the former involves probabilistic assessment before an event, while the latter involves post-hoc classification. The authors should consistently distinguish between these concepts throughout the paper.*

**Response 39:**

Thank you for raising this important conceptual issue. We recognize that the distinction between assessing manipulation risk and detecting manipulation behavior is not merely a wording issue, but directly affects how the model output should be interpreted and used. The revised manuscript now makes clear that our objective is manipulation risk assessment at the firm–month level, rather than manipulation detection in the sense of identifying confirmed manipulative transactions or actors, or classifying observations as confirmed manipulation cases.

In the revised manuscript, we have clarified this conceptual boundary throughout the paper. We have restricted the use of “manipulation detection” to discussions of prior studies or tasks that aim to identify manipulative behavior after the fact, often using transaction-level or enforcement-confirmed evidence. By contrast, the present study develops a screening-oriented risk assessment framework based on publicly observable disclosure, sentiment, and trading signals. The model output is therefore interpreted as a probabilistic risk score used for risk ranking, candidate review, and tiered screening, rather than as direct evidence of manipulation.

Following this clarification, we revised the wording in the Introduction, Section 3.1, Section 4.1, the empirical interpretation, and the Conclusion. When describing the objective, model output, empirical findings, and practical implications of this study, we now consistently use terms such as risk assessment, risk score, risk ranking, screening, and candidate review. This revision aligns the terminology with the methodological design and prevents the model from being overinterpreted as a transaction-level manipulation detection system.

**Amendments in the Manuscript:**

To address these limitations, this study aims to assess stock market manipulation risk by developing a multi-source analytical framework that integrates corporate disclosures, investor sentiment, and market trading behavior. **(Page 3, Section 1, lines 52–53)**

……

Third, it formulates stock market manipulation analysis as a probabilistic risk assessment problem that supports risk ranking and tiered screening in regulatory practice, and further enhances interpretability through SHAP (SHapley Additive exPlanations). **(Page 3, Section 1, lines 65–67)**

……

Based on this perspective, the present study examines whether manipulation risk can be systematically captured by observable joint deviations across disclosure behavior, sentiment diffusion, and market responses. This study does not aim to identify specific manipulative transactions or actors at the transaction level, but instead focuses on assessing manipulation risk based on observable multi-source signals. **(Page 8, Section 3.1, lines 189–193)**

……

Across all models, the output is interpreted as a probabilistic risk score reflecting manipulation-related risk. This interpretation is consistent with the screening-oriented objective of the study, where the model is used to prioritize high-risk observations. **(Page 15, Section 4.1, lines 341–343)**

……

From a practical perspective, the value of the framework lies in converting heterogeneous public signals into a ranked list of firm–month observations for further review. **(Page 31, Section 5, lines 698–700)**

**Comment 40:**

*The generalizability of the results beyond the Chinese market is not discussed at all. This limits the theoretical value of the article for an international audience and should be clearly reflected in the limitations section.*

**Response 40:**

Thank you for this important comment. We recognize that the generalizability of the findings to other markets should be discussed more explicitly.

In the revised manuscript, we have added this issue to the limitations and future research discussion. The proposed framework is built on publicly observable signals, including disclosure behavior, online public sentiment, and market trading characteristics. This general logic may be relevant to other markets where manipulation risk is also reflected in information release, investor attention, and trading responses. However, the empirical performance and practical interpretation of the model may vary across institutional environments. Differences in disclosure regulation, enforcement procedures, investor composition, market microstructure, and online discussion platforms may affect how these signals are generated and how they should be interpreted.

Therefore, we now state more clearly that the current empirical evidence should be interpreted in the context of China’s A-share market and CSRC-sanctioned cases. We also acknowledge that the adaptability of the framework to non-Chinese market settings requires further examination, and that cross-market evidence would help assess the generalizability of the proposed framework.

**Amendments in the Manuscript:**

Second, although the model exhibits overall stability within the current sample, its adaptability to extreme market conditions, emerging manipulation patterns, undetected manipulation cases, and non-Chinese market settings warrants further examination. Future research could extend this framework by incorporating higher-frequency trading records, event-level annotations, and richer investor-structure information. Cross-market evidence would also help assess the generalizability of the framework beyond China’s A-share market. **(Page 31, Section 5, lines 710–714)**

Response to Reviewer 3:

**Comment 1:** *Conceptual Framework and Theoretical Depth*

*The authors effectively argue that modern manipulation is a "multi-source coupling" phenomenon. However, the transition from the theoretical drivers discussed in Section 3.1 to the specific variables selected in Section 3.2 could be more explicitly linked. For instance, while the paper mentions "nonlinear feedback loops" in manipulation risk, the current supervised learning framework primarily treats these features as parallel inputs. A more detailed discussion on how the model specifically captures the interaction or temporal lag between an announcement (information) and the subsequent sentiment surge (reaction) would strengthen the "chain" argument.*

**Response 1:**

Thank you for this thoughtful comment. The reviewer’s point helped us recognize that the original manuscript needed to explain more clearly how the conceptual chain discussed in Section 3.1 is translated into the variable system and the empirical model. We have therefore revised the manuscript to make clear that the framework is not a simple parallel collection of indicators. Rather, our purpose is to represent manipulation risk through observable signals from three connected dimensions: information disclosure, public sentiment diffusion, and market trading response.

In the revised manuscript, we strengthened this connection from theory to variables. Section 3.1 now explains that the framework is built on three complementary information dimensions. Formal disclosures reflect how listed firms release and frame information; public sentiment captures investor attention, discussion intensity, and expectation diffusion; and market trading variables describe how these signals are reflected in returns, trading activity, volatility, and market value changes. Section 3.2 then maps these theoretical dimensions directly to the three feature groups: announcement-text features, public sentiment features, and market trading features. This revision makes clear that the variables are not intended as isolated predictors, but as observable components of a multi-source risk representation framework.

We also clarified how the model operationalizes this framework. The current supervised learning design does not estimate a structural causal lag from a specific announcement to a subsequent sentiment surge and then to a trading response. Instead, it evaluates whether these heterogeneous signals jointly improve firm–month risk ranking. In the final fusion model, the BERT–MIL branch aggregates multiple announcements into a firm–month textual representation, while the structured-feature branch processes disclosure indicators, public sentiment variables, and market trading variables. These representations are then concatenated and passed through the MLP prediction layer, allowing the model to learn nonlinear combinations among announcement semantics, sentiment diffusion indicators, and market response variables within the same firm–month risk window.

Accordingly, we refined the interpretation of the “information disclosure–sentiment reaction–trading behavior” chain. In the revised manuscript, this chain is presented as a risk representation logic for screening-oriented assessment, rather than as a formally estimated event-level causal process. We also acknowledge that more granular lead–lag mechanisms between announcements, sentiment bursts, and trading responses would require higher-frequency or event-level data, which we now identify as an important direction for future research.

**Amendments in the Manuscript:**

In Section 3.1, we strengthened the theoretical link between the conceptual framework and the variable system by clarifying the complementary roles of the three information dimensions:

These three dimensions correspond to the main feature groups used in this study: announcement-text features, public sentiment features, and market trading features. They do not represent isolated hypotheses for individual variables, but jointly define a risk representation framework. From a dynamic perspective, manipulation risk may be reflected when disclosure patterns, sentiment diffusion, and market responses reinforce one another within the same risk process. This multi-source perspective therefore provides the theoretical basis for the variable system constructed in the following section. **(Page 9, Section 3.1, lines 202–206)**

In Section 3.2, we revised the variable construction discussion to clarify how each feature group corresponds to the theoretical dimensions of disclosure behavior, sentiment diffusion, and market trading response. **(Pages 9–13, Sections 3.2.1–3.2.3, lines 209–306)**

In Section 4.1, we clarified how the model fuses the textual and structured representations:

In the final fusion step, the BERT–MIL branch aggregates announcement embeddings into a firm–month textual representation, while the structured-feature branch processes structured announcement indicators, public sentiment variables, and market trading variables in parallel. The two representations are then concatenated and passed to a Multi-Layer Perceptron (MLP) prediction layer, yielding the BERT + MIL + MLP fusion model. **(Page 15, Section 4.1, lines 331–334)**

In the Conclusion, we further clarified that the model represents manipulation risk through observable joint deviations rather than establishing an event-level causal sequence:

The findings suggest that manipulation risk is not fully reflected by a single anomalous factor; rather, observations with elevated risk tend to exhibit joint deviations across disclosure behavior, sentiment diffusion, and market responses. Firm–month observations showing such concurrent deviations are assigned higher risk scores by the model. The model demonstrates stable performance and screening utility in prioritizing observations with elevated risk, indicating that multi-source fusion can improve risk ranking when individual indicators are noisy or incomplete. Accordingly, manipulation risk is better represented as a structured configuration of observable signals rather than as a simple aggregation of isolated indicators. **(Pages 30-31, Section 5, lines 688–694)**

**Comment 2:** *Methodological Rigor and BERT Implementation*

*The use of BERT for extracting features from corporate announcements is a modern and appropriate choice. To improve transparency, the authors should provide more details on the fine-tuning process of the BERT model. Specifically, it is important to clarify if the BERT model was pre-trained on a general Chinese corpus or a specialized financial corpus, as the latter significantly impacts the capture of nuanced financial sentiment and "fog" (complexity) indices. Additionally, since the "announcement fog" is a key variable, the authors should detail how semantic complexity is numerically derived from the BERT embeddings to ensure replicability.*

**Response 2:**

Thank you for this thoughtful and constructive comment. We agree that the original manuscript did not sufficiently clarify the implementation of the BERT text encoder or the relationship between the BERT-based text representation and the manually constructed announcement complexity indicator.

In the revised manuscript, we have clarified the BERT implementation in Section 4.1.2. The text encoder used in the main experiments is Chinese RoBERTa-wwm-ext, a general-purpose Chinese pretrained model with whole-word masking. For each cleaned announcement text, the [CLS] embedding is extracted as the announcement-level representation. Given the limited size of sanction-based labeled samples, the BERT encoder is frozen in the main experiments, while the MIL and fusion layers learn task-specific risk representations.

We also clarified that announcement textual complexity is not numerically derived from BERT embeddings. Instead, ann_fog is constructed as an interpretable structured readability indicator in Section 3.2.1. Specifically, the manuscript defines ann_fog using a Chinese-adapted readability formulation based on average sentence length and the complex-word ratio, where the complex-word ratio is measured as the proportion of segmented words containing three or more Chinese characters. This formulation follows the linguistic adaptation already described in the variable construction section and is separate from the BERT-based semantic representation.

This distinction clarifies the complementary roles of the two text-related components. The BERT–MIL branch learns contextual semantic representations directly from announcement texts, whereas ann_fog provides a transparent and reproducible measure of textual complexity. In the final fusion model, the BERT–MIL announcement representation and the structured variables, including ann_fog, are combined to support manipulation risk assessment.

**Amendments in the Manuscript:**

In the empirical implementation, we use Chinese RoBERTa-wwm-ext, a general-purpose Chinese pretrained model with whole-word masking, and extract the [CLS] embedding from each cleaned announcement text. Given the limited size of sanction-based labeled samples, the BERT encoder is frozen, while the MIL and fusion layers learn task-specific risk representations. (**Page 17, Section 4.1.2, lines 389-392)**

The original Gunning Fog Index was developed for English texts and identifies complex words based on syllable structure, which is not directly applicable to Chinese. Drawing on Chinese financial-text readability studies, particularly Li et al. [44], this study constructs the index based on average sentence length and the complex-word ratio:

$${\text{ann}\text{\_f}\text{og}}_{\text{i}}\text{=}\text{ASL}_{\text{i}}\text{+}\text{CWR}_{\text{i}}$$

(2)

where $\text{ASL}_{\text{i}}\text{ }$denotes the average sentence length, calculated as the total number of segmented words divided by the number of sentences, and $\text{CWR}_{\text{i}}\text{ }$ represents the complex-word ratio, calculated as the proportion of segmented words containing three or more Chinese characters. A higher value of ann_fog indicates greater textual complexity and lower readability. **(Pages 10-11, Section 3.2.1, lines 239–245)**

**Comment 3:** *Data Labeling and Potential Bias*

*The study relies on "retrospectively labeling violation periods" based on CSRC-sanctioned cases. While this is a standard approach, it introduces a "survivorship bias" where the model only learns from manipulators who were caught. The authors should include a "Limitations" section discussing how the features of detected manipulation might differ from undetected manipulation. Furthermore, the firm-month granularity is practical for regulation but might smooth over high-frequency "pump and dump" schemes that occur within days; a justification for the monthly frequency over weekly or daily windows would be beneficial.*

**Response 3:**

Thank you for this important and thoughtful comment. We agree that using CSRC-sanctioned cases to retrospectively label violation periods may introduce a detected-case bias. Although administrative penalty cases provide legally documented manipulation intervals and are therefore a practical and credible source for supervised labeling, they only cover manipulation cases that were detected, investigated, and sanctioned by regulators. Undetected manipulation may differ from sanctioned cases in terms of trading intensity, information channels, duration, and observability. We have therefore clarified this limitation in the revised manuscript.

In the revised Conclusion, we now explicitly state that the labels are constructed from ex post regulatory penalty documents and that the model primarily captures statistical associations between observable public signals and retrospectively identified violation periods. We also acknowledge that the adaptability of the model to undetected manipulation cases requires further examination. This revision is intended to prevent overgeneralizing the model from sanctioned cases to all possible manipulation behaviors.

Regarding the firm–month granularity, we agree that this temporal scale may smooth short-horizon manipulation patterns, especially pump-and-dump schemes that unfold within a few trading days. In this study, the monthly frequency is used because the objective is screening-oriented risk assessment under a unified multi-source data structure. Corporate disclosures, online public sentiment variables, and market trading indicators are aligned at the firm–month level to support periodic risk ranking and candidate review. This design is suitable for front-end regulatory prioritization, but it is not intended to identify intraday or event-level manipulation mechanisms.

We have therefore clarified that the monthly setting is a practical regulatory screening unit rather than a high-frequency detection window. We also note in the limitations that future research could incorporate higher-frequency trading records and event-level annotations to better capture short-horizon manipulation patterns.

**Amendments in the Manuscript:**

In Section 4.2.1, we clarified the retrospective nature of the labeling strategy:

The sanction records are used ex post to define supervised learning labels and are not used as explanatory variables in model training. The model is therefore interpreted as learning observable public-data patterns associated with retrospectively identified violation months and producing periodic manipulation-risk scores. **(Page 19, Section 4.2.1, lines 431-433)**

In the Conclusion, we expanded the limitation discussion as follows:

Despite these findings, the current model has several limitations. First, constrained by public data availability and labels constructed from ex post regulatory penalty documents, the model does not incorporate transaction-level order or trade information and therefore primarily captures statistical associations rather than directly identifying specific manipulative actions. Accordingly, the model output should be interpreted as a probabilistic risk score for candidate review and prioritization, and should be considered together with regulatory judgment and contextual verification. Legitimate information shocks, market-wide events, or sector-level fluctuations may also generate abnormal returns, sentiment bursts, and trading fluctuations, thereby increasing false positive risk in screening applications. Second, although the model exhibits overall stability within the current sample, its adaptability to extreme market conditions, emerging manipulation patterns, undetected manipulation cases, and non-Chinese market settings warrants further examination. Future research could extend this framework by incorporating higher-frequency trading records, event-level annotations, and richer investor-structure information. Cross-market evidence would also help assess the generalizability of the framework beyond China’s A-share market. **(Page 31, Section 5, lines 704–714)**

**Comment 4:** *Interpretability and SHAP Analysis*

*The application of SHAP to provide "actionable insights" is a highlight of the paper. The authors should expand Figure 5 and the related text to discuss not just the global importance of features like "CAR" (Cumulative Abnormal Return) or "ann_cnt," but also the direction of their influence. For example,* *does a high "ann_fog" consistently increase or decrease the risk probability in the model’s view? Providing a case study of a specific "Risk (1)" firm and its SHAP waterfall plot would bridge the gap between abstract probability and regulatory practice.*

**Response 4:**

Thank you for this constructive and very helpful comment. We agree that the original SHAP analysis relied too heavily on global feature importance and did not sufficiently explain the direction, heterogeneity, and case-level meaning of feature effects. We also agree that a local explanation is important for linking an abstract model score to practical review prioritization.

In the revised manuscript, we expanded the SHAP analysis in three ways. First, we retained the global importance analysis in Figure 5 but revised the interpretation to avoid treating feature importance as directional influence. Second, we expanded Figure 6 and the accompanying text to discuss the direction and heterogeneity of SHAP effects. The revised analysis shows that CAR has a relatively clear positive association with the model’s risk score: observations with higher CAR values are mainly distributed on the positive SHAP side. By contrast, ann_fog and ann_sent show more conditional patterns. Higher values of these disclosure-derived indicators are more often associated with negative SHAP contributions, but their distributions are not strictly one-sided. We therefore interpret textual complexity and announcement tone as context-dependent signals rather than mechanical risk amplifiers or risk reducers.

Third, we added Figure 7, a local SHAP waterfall plot for a representative true-positive firm–month observation. This observation receives a predicted risk probability of 0.976. The local explanation shows how multiple variables jointly raise the risk score, including announcement volume, CAR, market capitalization change, announcement sentiment, posting volume, monthly return, and trading value relative to circulating market capitalization. It also shows offsetting contributions from other variables. This case-level explanation helps translate the global SHAP results into an observation-level risk profile and illustrates how the model output can support screening-oriented review prioritization.

We also clarified in the manuscript that the SHAP analysis is applied to the structured numerical branch of the final BERT + MIL + MLP fusion model. This clarification helps avoid conflating structured-feature explanations with token-level interpretation of the BERT announcement-text encoder.

**Amendments in the Manuscript:**

**Fig 7. Local SHAP explanation for a high-risk observation.**

To examine how the structured numerical variables contribute to the final fusion model, this study applies SHapley Additive exPlanations (SHAP) to interpret the numerical branch of the BERT + MIL + MLP model. Figure 5 reports the global feature importance based on the mean absolute SHAP value; Figure 6 illustrates the direction and heterogeneity of feature effects; and Figure 7 provides a local explanation for a representative high-risk observation.

As shown in Figure 5, the CAR variable ranks first in the global importance results, indicating that abnormal return deviations provide the strongest structured signal for the model’s risk scoring. This finding is consistent with the role of price responses in market manipulation cases, where abnormal returns often represent an observable market-level manifestation of risk. Announcement volume (ann_cnt) and announcement textual complexity (ann_fog) rank immediately after CAR, suggesting that formal disclosure behavior provides substantial information in addition to market trading variables. Sentiment-related indicators, including posting volume (post_cnt) and the daily variability of posting activity (post_cnt_std_day), also appear among the important features, suggesting that the model assigns greater importance to the intensity and temporal clustering of investor attention than to passive information exposure. These results indicate that the fusion model captures not only abnormal price movements, but also variation in corporate disclosure behavior and the concentration of investor attention.

……

Figure 6 further illustrates the directionality and heterogeneity of feature effects. Along the CAR dimension, observations with high CAR values are mainly distributed on the positive SHAP side, indicating that larger abnormal return deviations tend to increase the risk score assigned by the model. By contrast, announcement textual complexity (ann_fog) and announcement sentiment (ann_sent) show more conditional patterns. Higher values of these disclosure-derived indicators are more often associated with negative SHAP contributions, but their distributions are not strictly one-sided and the effects can vary across observations. This suggests that textual complexity and announcement tone should not be interpreted as mechanical risk amplifiers or risk reducers. Their contributions depend on whether changes in readability or tone coincide with abnormal trading activity and sentiment diffusion. Sentiment variables also display nonlinear effects, with extreme observations contributing more strongly to the risk score in some cases. This pattern indicates that abrupt shifts in information diffusion may serve as risk-relevant cues in the model’s assessment.

Figure 7 illustrates this logic at the observation level. The selected true-positive firm–month observation receives a high predicted risk probability of 0.976. Its local explanation shows that the risk score is raised by features from multiple dimensions, including announcement volume, CAR, market capitalization change, announcement sentiment, posting volume, monthly return, and trading value relative to circulating market capitalization. At the same time, several variables contribute in the opposite direction, indicating that the final risk score results from the balance of reinforcing and offsetting signals rather than from a single feature. This local explanation helps translate the global SHAP results into an observation-level risk profile and illustrates how the model can support review prioritization in a screening context.

Overall, the SHAP results provide three implications for manipulation risk assessment. First, price response variables remain important, but the model’s risk assessment output is not limited to a single market indicator. Second, disclosure behavior and sentiment diffusion provide complementary information when they evolve jointly with market trading patterns. Third, the local explanation shows how a high-risk score can be traced back to a structured combination of feature contributions. From a regulatory perspective, this suggests that risk screening should not rely only on price anomalies, but should also monitor whether abnormal returns coincide with unusual disclosure intensity, shifts in disclosure tone and readability, and concentrated investor attention. Given the high importance of CAR in the SHAP results, Section 4.3.2 further examines the robustness of the fusion model after excluding CAR from the structured numerical feature set. **(Pages 25-27, Section 4.2.2, lines 564–618)**

**Comment 5:** *Comparative Performance and Baseline Robustness*

*The empirical results show the fusion model outperforms single-source baselines. To make this more robust, the authors should compare their fusion approach against other state-of-the-art ensemble methods or graph-based models (like GCNs) which are increasingly used to capture "interconnected price reactions" mentioned in the introduction. This would place the BERT-fusion framework within the current frontier of RegTech research.*

**Response 5:**

Thank you for this insightful and constructive comment. We appreciate the reviewer’s suggestion that the proposed BERT-fusion framework should be positioned more clearly against advanced modeling approaches in current RegTech research.

In the revised manuscript, we strengthened the robustness of the comparative evaluation in several ways. Model performance is now reported as mean ± standard deviation across five random seeds, paired significance tests are added to examine whether the performance differences between models are statistically supported under the same repeated partitions, and a chronological validation is included to assess temporal generalizability. These revisions provide a more reliable basis for evaluating the comparative performance of the fusion framework. The existing comparison also includes CatBoost as a nonlinear gradient-boosting ensemble baseline for the structured numerical features.

Regarding graph-based models such as GCNs, we agree that they are an important frontier for modeling interconnected market reactions when suitable relational data are available. However, the dataset used in this study is organized as firm–month observations constructed from public disclosures, online public opinion, and market trading variables. It does not contain an explicit relational structure, such as firm–firm links, investor–firm networks, ownership connections, fund-flow networks, transaction-counterparty relations, or social interaction networks. Therefore, applying a GCN in the current setting would require imposing an artificial graph structure rather than learning from an observed economic or informational network.

Constructing such a graph from return comovement or price correlations could embed market outcomes directly into the model structure and introduce additional endogeneity, especially given the reviewer’s related concern that price-response variables may already contain outcome-related information. For this reason, we did not add a GCN baseline in the current study. Instead, we revised the interpretation of the comparative results more cautiously: the reported results show that the fusion model improves risk ranking relative to the selected structured-feature, nonlinear ensemble, and text-only baselines under the same firm–month risk assessment setting, rather than demonstrating superiority over all possible advanced architectures. We also identify graph-based extensions as an important direction for future research when suitable relational data become available.

**Amendments in the Manuscript:**

To reduce dependence on a particular random partition, all random split experiments are repeated using five random seeds, and Table 3 reports model performance as mean ± standard deviation. The same random partitions are used across models, which makes the model comparisons paired at the seed level. Rather than relying only on point estimates, Table 4 further examines whether the performance differences between the final fusion model and each comparison model are statistically supported under this paired design. The relatively small standard deviations of the fusion model, particularly for AUC, PR-AUC, and Recall@20%, indicate that its performance is not highly sensitive to a particular random split. **(Page 23, Section 4.2.2, lines 516–521)**

We added Section 4.3.3 to report the chronological validation results:

4.3.3	Temporal Validation

To complement the repeated random split evaluation with a chronological design, this study further conducts a temporal validation. The models are trained on observations from 2015 to 2020, validated on observations from 2021, and evaluated on observations from 2022 to 2023. By preserving the calendar order of the sample, this design assesses the temporal generalizability of the risk assessment framework.

Table 8 reports the temporal validation results. The fusion model achieves the highest AUC and PR-AUC, with values of 0.8903 and 0.7096, respectively. It also obtains the highest Recall@20% of 0.7273, while its Recall@10% is equal to that of the Logit model. Although CatBoost attains a competitive AUC, its lower PR-AUC and Recall@K indicate weaker performance in prioritizing observations with elevated risk. The BERT + MIL model using only announcement text performs less favorably under the chronological split, suggesting that announcement semantics alone are more sensitive to temporal changes than the fused representation.

Overall, the temporal validation provides complementary evidence for the stability of the multi-source framework. While the repeated random split results evaluate average model performance under alternative sample partitions, the chronological split examines whether the learned risk patterns remain informative when the evaluation period moves forward in time. The results show that combining announcement semantics, public sentiment, and market trading signals preserves effective risk ranking ability in this temporal setting. Thus, the chronological validation should be interpreted as a complementary assessment of temporal generalizability, whereas the repeated random split results in Table 3 are reported as an average-performance benchmark for model comparison. **(Pages 29-30, Section 4.3.3, lines 663–680)**

We further added Table 8 to report the temporal validation results.

**Table 8. Temporal validation results.**

| **Model** | **Input** | **AUC** | **PR-AUC** | **Recall@10%** | **Recall@20%** |
| --- | --- | --- | --- | --- | --- |
| **Logit** | Numerical features | 0.8308 | 0.5767 | 0.3939 | 0.6364 |
| **CatBoost** | Numerical features | 0.8693 | 0.5452 | 0.2727 | 0.5758 |
| **BERT + MIL** | Announcement text | 0.7144 | 0.4261 | 0.2121 | 0.3333 |
| **BERT + MIL + MLP** | Announcement text + numerical features | 0.8903 | 0.7096 | 0.3939 | 0.7273 |

**Note:** The chronological split uses 2015–2020 as the training period, 2021 as the validation period, and 2022–2023 as the test period. Metrics are reported on the temporal test period.

In the Conclusion, we clarified future extensions of the framework:

Future research could extend this framework by incorporating higher-frequency trading records, event-level annotations, and richer investor-structure information. Cross-market evidence would also help assess the generalizability of the framework beyond China’s A-share market. **(Page 31, Section 5, lines 712–714)**

Sincerely,

Yuting Luo

On behalf of all authors
