# Supplementary material for: Risk assessment of stock market manipulation through the fusion of multi-source textual and trading data: Evidence from China’s A-share market
Source: PLoS One. 2026 Aug 7;21(8):e0343442. doi: 10.1371/journal.pone.0343442 (PMC13450784; doi:10.1371/journal.pone.0343442)
Supplement: Supplementary file 4 [file pone.0343442.s002.docx]

**«Risk Assessment of Stock Market Manipulation through the Fusion of Multi-Source Textual and Trading Data»**

The paper contains significant methodological problems, is poorly formatted, and cannot be accepted for publication in its current form. The potential for further revision is questionable. Conclusion: Reject.

Reason for rejection:

Manipulation is stated as the subject of the study, but the type of manipulation is not specified: what specific violations were committed, and what manipulation strategies are used? For example, a pump and dump strategy or a strategy of sequentially accumulating positions over several months are significantly different from one another and, therefore, will exhibit different indicators of changes in trading performance. A single model cannot effectively detect all types of manipulation simultaneously.

The most important and fundamental point if we're going to discuss manipulation is that stock price manipulation involves very specific transactions and actions by an individual or group of individuals. The current methodology doesn't address this at all, and this important nuance is overlooked. The authors examine stock prices, not individual transactions, which are what constitute manipulative trading.

To reliably identify manipulation, it's necessary to have access to the order and trade register, research them, and learn to identify specific suspicious transactions performed by specific individuals. If this isn't available in the work, then one can say something like, "With a probability of XX%, during a certain observation period, there were suspicious changes in the exchange characteristics of a specific asset, accompanied by increased sentiment on social media and other factors."

A section with formal research hypotheses is missing. For a study using supervised learning, the research questions should at least be formulated as testable propositions.

Robustness checks are missing: changing the training period, alternative label binarization thresholds, excluding certain industries. Without these, it is difficult to assess the generalizability of the results.

The assertion that traditional methods "have become insufficient" is formulated declaratively (lines 11–12 in parentheses here and below), without supporting specific references. This thesis requires substantiation in the abstract—either a reference or a brief indication of the scale of the problem (e.g., the volume of identified manipulations or investor losses).

The model's superiority in terms of AUC, PR-AUC, and Recall@10%/20% metrics is claimed, but specific numerical values are not provided. This is a significant drawback—the reader cannot assess the practical significance of the authors' contribution.

The term "multi-source coupling nature" (21) is the author's own neologism, which has not gained currency in the literature. It is necessary to either provide a clear definition or replace the term with a standard one.

The introduction too quickly moves from the problem statement to the gaps in the literature, bypassing the rationale for the practical significance of the study for regulators (26–33). The question remains unanswered: how great is the damage from manipulation in the Class A share market? Without this, the motivation for the study appears declarative.

The description of existing work in the introduction (lines 35–46) duplicates the content of Section 2 (literature review). This creates structural redundancy. It is recommended to limit the introduction to the problem statement and the gap, moving the detailed literature review to the appropriate section.

The three stated "contributions" (lines 53–60) are not clearly stated. In particular, the second contribution ("incorporates deep semantic features") is applicable to any work using BERT. It is necessary to specify the novelty: how exactly does this architecture outperform its predecessors when applied to the task of manipulation detection?

In the literature review, the authors mechanically list works in each subsection (62–90) without providing a critical discussion. There is no comparison of competing approaches, no indication of contradictions between the results of different authors, and no explanation of why existing solutions are insufficient for the given context (Chinese Market A).

Citing Allen & Gorton (1992)—a work more than 30 years old—as a key theoretical framework without discussing more recent theoretical developments diminishes the relevance of the review (77–83). It is necessary to demonstrate how theoretical perspectives have evolved.

Section 2.2 (91–122) is devoted to the diffusion of public sentiment, but its connection to the main objective of the study (assessing the risk of manipulation) is weak. The authors cite studies on the prediction of returns and volatility (97–99, 102–103), but do not explain why these mechanisms are relevant specifically for detecting manipulative behavior, rather than simply information shocks.

Section 2.3, on multi-source data fusion (123–159), is primarily descriptive. It lacks a critique of the methodological approaches used in the cited studies. The authors should clearly indicate which methodological limitations of previous studies this article addresses.

Section 3.1 purports to provide a theoretical justification (162–186), but in reality represents a narrative description of the manipulation process without a formal model or testable hypotheses. If the study is empirical in nature, it is necessary to explicitly formulate at least testable working hypotheses (H1, H2, etc.) for each group of variables.

The ad sentiment index (formula 1) is calculated using the CFSD dictionary (199–208).

However, the authors do not justify the choice of this dictionary, do not compare it with alternatives (for example, financially-oriented versions of LIWC or specialized dictionaries for the Chinese stock market), and do not discuss its limitations when applied to corporate announcements.

The readability index (formula 2) is an adaptation of the Fog Index (209–218). The authors do not discuss its validity for Chinese: the Fog Index was developed for English and is based on a syllabic structure incompatible with Chinese morphology. There is no justification for choosing this measure for Chinese text—this is a serious methodological flaw.

The public sentiment variables (post_cnt, user_cnt, etc.) are taken from the Eastmoney Guba platform (219–243). The authors do not discuss how representative this platform is of the entire investor community. Guba is known to be primarily focused on retail investors, which may systematically bias sentiment estimates relative to institutional participants.

When describing the exchange variables (244–272), the authors do not justify the choice of a six-month window for calculating volatility (265–266). Why six months, and not three or twelve? The sensitivity of the results to the choice of window is not discussed.

The authors use monthly data for the dataset:

- this time period does not specify whether manipulation occurred every trading day or just a few days, or even just one day within the period.

- regarding investor sentiment: over the monthly observation period, if manipulation occurred and prices rose, positive sentiment and active discussions may be a consequence of the price increase; this correlates with manipulation, but is not directly related to it.

The authors base their study on a random 80/20 split of the sample and five repetitions (301–302). However, when working with panel data at the firm-month level, random splitting creates data leakage: observations from the same firm in different months appear in both the training and test samples. A firm-level split or a time split is necessary to eliminate this effect.

The training and test samples are split randomly (80/20), without regard to the time structure. It would be more methodologically sound to use the same periods as the training sample (2015–2021 = 70% of observations and 2022–2023 (30% of observations) or to split them as proposed 80%/20%, but in chronological order.

The methodology is insufficiently described: it doesn't describe how the authors calculate CAR; which BERT model is used; which MLP model parameters are used; or how the texts were preprocessed. The lack of study details that could be included in an appendix makes the results difficult to reproduce and complicates their interpretation.

The paper doesn't mention excluding observation periods from the sample during which market factors could have influenced significant price changes. Are there plans to factor these into the model? Without this refinement, it's difficult to apply the model in practice, as the authors propose, as the number of false positives could increase significantly.

The matching method (1:5, nearest-neighbor by month, industry, and market capitalization) is described briefly. The authors do not provide diagnostics for the quality of the matching: a covariate balancing table or a test for statistical equivalence of the groups after matching. Without these, it is impossible to assess whether systematic selection bias has been eliminated.

A 1:5 offender-to-nonoffender ratio creates a class imbalance of approximately 16–17% of positive observations (according to Table 2, (383–388)). The authors mention PR-AUC as a metric for imbalanced classes, but do not describe the use of any resampling techniques (SMOTE, oversampling) or class weighting during model training. This omission is particularly critical for Logit and CatBoost.

The use of pre-trained BERT for Chinese requires clarification: which specific version is used (Google's Chinese BERT base, MacBERT, FinBERT-zh, or another)? (337–356) The financial domain differs significantly from the general-purpose corpora on which the standard BERT is trained. The lack of domain-specific fine-tuning (finetuning on financial texts) or its justification reduces the credibility of the results.

The architecture of the final fusion BERT+MIL+MLP is described at a conceptual level (358–375), but technical details are completely missing: the dimensionality of the hidden layers of the MLP, activation functions other than sigmoid, dropout parameters, optimization algorithm, learning rate, and number of epochs. Without these, reproducibility of the study is impossible.

The explanation for the concentration of violations in 2017–2020 by the "retrospective labeling" mechanism (lines 414–419) is logically correct, but raises an unsolved problem: is there a survivorship bias—a systematic underrepresentation of earlier violations for which the statute of limitations expired before 2022? The authors do not discuss this source of bias.

The use of Cohen's d to characterize the separating power of variables (422–435) is methodologically acceptable; however, the authors interpret negative d values for ann_sent as evidence that "manipulative firms use a less positive tone" (pp. 204–208), without conducting statistical tests for the significance of the differences. P-values or confidence intervals for each Cohen's d should be included.

Table 3 is the key result of the article. However, the authors (448–479) do not conduct statistical significance tests of the differences between the models (e.g., the Deming–McNemar test for AUC or bootstrap tests). The gap between BERT+MIL and the final model in AUC (0.8680 vs. 0.8767) is small, and without a significance test, it cannot be concluded that the increase is not due to chance.

The authors report repeating the experiment five times and averaging the results (449–450), but do not provide standard deviations of the metrics across iterations. Without measures of variability, it is impossible to assess the stability of the results.

SHAP analysis is only performed for the final model (485–512). To make the interpretation more convincing, it would be worth comparing the feature importance between CatBoost and BERT+MIL+MLP to demonstrate how the addition of text features changes the contribution of trading variables.

The dominance of CAR (cumulative abnormal returns) in the SHAP rating (487–489) raises a fundamental methodological question: CAR is effectively the outcome of manipulation, not its leading indicator. If the model relies primarily on this indicator, it essentially detects manipulation after the fact, rather than predicting risk. This contradicts the stated goal of "front-end risk screening" (32–33, 528–529) and requires serious discussion.

There is no discussion in the article

In conclusion, the finding that "manipulation manifests itself through the coordinated evolution of signals" is a descriptive observation, not a rigorously proven assertion. The study does not test causal relationships between disclosure, sentiment, and trading; it establishes predictive associations. The authors should clearly distinguish between these concepts.

The claim that the model is applicable to "risk stratification" and "tiered supervision" (524–530) is not supported by an analysis of threshold calibration or an assessment of the probability of false positives under real-world screening volumes. Practical applicability remains declarative.

The authors acknowledge (531–532) that the model "captures statistical associations rather than causal mechanisms." This is a significant limitation, but its discussion takes only one sentence. It needs to be expanded: what specific conclusions for regulators might be erroneous if the associations are not causal?

(line 70): "the CSRC" is written twice—a typo that must be corrected.

In several places, the article uses the terms "manipulation risk" and "manipulation detection" interchangeably, although conceptually these are different tasks: the former involves probabilistic assessment before an event, while the latter involves post-hoc classification. The authors should consistently distinguish between these concepts throughout the paper.

The generalizability of the results beyond the Chinese market is not discussed at all. This limits the theoretical value of the article for an international audience and should be clearly reflected in the limitations section.
