# Supplementary material for: Risk assessment of stock market manipulation through the fusion of multi-source textual and trading data: Evidence from China’s A-share market
Source: PLoS One. 2026 Aug 7;21(8):e0343442. doi: 10.1371/journal.pone.0343442 (PMC13450784; doi:10.1371/journal.pone.0343442)
Supplement: Supplementary file 5 [file pone.0343442.s005.docx]

**Response to Reviewers**

Manuscript number: PONE-D-26-06350

Title: Risk assessment of stock market manipulation through the fusion of multi-source textual and trading data: Evidence from China’s A-share market

**Dear** **Editor and Reviewers,**

Thank you very much for the opportunity to revise our manuscript entitled “Risk assessment of stock market manipulation through the fusion of multi-source textual and trading data: Evidence from China’s A-share market” (PONE-D-26-06350). We sincerely appreciate the editor’s and reviewers’ careful evaluation, constructive comments, and valuable suggestions.

In this second revision, we have focused on the remaining substantive concern regarding the manuscript’s theoretical foundation and discussion. Accordingly, we revised the conceptual framing of the study and added a standalone Discussion section, while also addressing the final clarification suggestions raised by Reviewer 3.

We are grateful for the time and effort that the editor and reviewers have devoted to evaluating our work. We hope that the revised manuscript and the accompanying responses adequately address the concerns raised, and we respectfully submit the revised manuscript for further consideration.

**Response to Editor:**

Thank you for the editorial guidance and for the opportunity to revise our manuscript again. We have carefully addressed the remaining concern raised by Reviewer 2 and the final suggestions provided by Reviewer 3.

In response to the editor’s instruction that the remaining comments of Reviewer 2 should be further addressed, we made targeted structural revisions to the manuscript. Specifically, we revised Section 3 and Section 3.1 to make the conceptual foundation of the study more explicit. Section 3 has been retitled “Multi-source Risk Representation and Variable Construction,” and Section 3.1 has been retitled “Conceptual Basis of Multi-source Risk Representation.” In the revised Section 3.1, we clarify how manipulation-related risk can be represented through observable public signals across formal disclosure behavior, public sentiment diffusion, and market trading responses.

We also added a standalone Discussion section as the new Section 5. This section discusses the implications of the study from three perspectives: conceptual and theoretical implications, methodological and empirical implications, and regulatory implications and boundary conditions. The new Discussion section explains how the findings contribute to a multi-source conceptualization of manipulation risk, how the empirical results support the complementary value of textual and structured information, and how the proposed framework should be interpreted in regulatory screening practice.

In addition, we addressed the final suggestions of Reviewer 3 by checking acronym definitions in the main text, clarifying the first appearance of BERT–Multiple Instance Learning (BERT–MIL), explicitly highlighting the CAR ablation result in the Discussion section, and adding a sentence acknowledging that temporal performance differences may reflect changes in market conditions, information environments, and regulatory enforcement patterns over time.

We have prepared the required revision files, including this point-by-point response letter, a marked-up revised manuscript, and a clean revised manuscript, in accordance with the journal’s submission requirements. We hope that these revisions address the remaining concerns and further improve the theoretical clarity, discussion depth, and practical interpretability of the manuscript.

Response to Reviewer 2:

**Comment:**

*This study still needs a theoretical foundation and a discussion parts. It was suggested to you in my first review.*

**Response:**

We sincerely thank the reviewer for raising this important concern again. We fully understand that, although we strengthened the literature review and empirical interpretation in the previous revision, the manuscript still needed to present its theoretical foundation and discussion section more explicitly and visibly. We appreciate this suggestion, as it helped us further improve the structure, conceptual clarity, and scholarly presentation of the manuscript.

In the present revision, we made two main revisions in direct response to this concern.

First, we revised Section 3 and Section 3.1 to make the conceptual foundation of the study clearer. Section 3 has been retitled “Multi-source Risk Representation and Variable Construction,” and Section 3.1 has been retitled “Conceptual Basis of Multi-source Risk Representation.” In the revised Section 3.1, we clarify how manipulation-related risk can be represented through observable public signals across formal disclosure behavior, public sentiment diffusion, and market trading responses. We also further explain that this study adopts a screening-oriented risk assessment perspective rather than a transaction-level manipulation detection perspective. Because the study is designed as a supervised machine-learning risk assessment framework rather than a traditional causal hypothesis-testing study, we do not formulate H1/H2-style hypotheses. Instead, we now present a clearer conceptual basis for understanding manipulation-related risk as a multi-source signal representation problem at the firm–month level.

Second, we added a standalone Discussion section. The new Section 5 discusses the implications of the study from three perspectives: conceptual and theoretical implications, methodological and empirical implications, and regulatory implications and boundary conditions. This section explains how the findings contribute to a multi-source conceptualization of manipulation risk, how the empirical results support the complementary value of textual and structured information, and how the proposed framework should be interpreted in regulatory screening practice. We also clarify the boundary conditions of the model, including the use of ex post CSRC penalty labels, the firm–month data structure, and the absence of transaction-level order and trade records.

We hope that these revisions adequately address the reviewer’s concern by making both the theoretical foundation and the discussion of the study more explicit, systematic, and consistent with the screening-oriented objective of the manuscript.

**Amendments in the Manuscript:**

The main amended text is reproduced below. We revised the title of Section 3 and Section 3.1 as follows:

3	Multi-source Risk Representation and Variable Construction

3.1	Conceptual Basis of Multi-source Risk Representation

In modern stock markets, price formation reflects the interaction between formal corporate disclosures, online public sentiment, and market trading responses. Information diffusion can influence investors’ beliefs and expectations, thereby affecting price adjustment, volatility, and trading activity [36]. Recent evidence further suggests that manipulation risk may increase in opaque or uncertain information environments, where investors face greater difficulty distinguishing fundamental price movements from strategically induced market signals [37]. This implies that manipulation-related risk should not be assessed solely through isolated trading outcomes, because abnormal market behavior may be accompanied by changes in disclosure patterns and information diffusion. A multi-source information perspective therefore provides the conceptual basis for representing manipulation-related risk through observable public signals and guides the construction of the firm–month risk variables used in the empirical model.

In practice, stock market manipulation may arise through heterogeneous channels. Some strategies are mainly trade-based and are reflected in abnormal price or volume movements, whereas others are more information-based and involve misleading disclosure, rumor diffusion, or sentiment amplification. In many cases, trading and information channels may also interact, forming combined manipulation patterns. These strategies differ in their underlying mechanisms and observable manifestations. Given such heterogeneity, this study does not seek to identify or classify specific manipulation strategies, but instead adopts a screening-oriented risk assessment perspective. The objective is to assess whether common observable risk patterns across heterogeneous manipulation forms can be represented through the joint dynamics of disclosure behavior, sentiment diffusion, and market responses. Accordingly, the model output should be interpreted as a firm–month manipulation-risk score for candidate review, rather than as transaction-level evidence of specific manipulative actions or actors.

Within this conceptual framework, manipulation-related risk can be represented through the complementary roles of three observable information dimensions. First, formal disclosures issued by listed firms reflect how firms release, frame, and communicate information to the market. Disclosure frequency, tone, textual complexity, and reporting patterns may influence investor interpretation and provide observable signals of the firm’s information environment [6,30,38]. Second, public sentiment generated by market participants captures investor attention, discussion intensity, and the diffusion of expectations in online information environments. Prior studies suggest that investor sentiment, rumors, and platform-based interactions are associated with market reactions and abnormal information diffusion [21,22,25]. Third, market responses capture the market-level manifestation of risk signals through returns, volatility, trading intensity, and market value changes. Evidence on public information manipulation and trading-based manipulation further indicates that such risks may be reflected in asset prices through market feedback and abnormal trading responses [20,39]. These three dimensions correspond to the main feature groups used in this study: announcement-text features, public sentiment features, and market trading features. They are not intended to support isolated variable-level hypotheses; rather, they jointly define the observable signal space for multi-source risk representation. From a dynamic perspective, manipulation risk may be reflected when unusual disclosure patterns, concentrated sentiment diffusion, and abnormal market responses co-occur within the same firm–month risk window. The following section operationalizes this conceptual framework by constructing variables that capture each of these three dimensions. **(Pages 8–9, Section 3.1, lines 170–206)**

We also added a new standalone Discussion section:

5	Discussion

5.1	Conceptual and Theoretical Implications

The results support a multi-source conceptualization of stock market manipulation risk at the firm–month level. Manipulation-related risk is not fully captured by isolated trading anomalies, nor is it adequately represented by disclosure or sentiment indicators alone. Rather, elevated risk appears as a configuration of observable public signals spanning formal disclosure behavior, public sentiment diffusion, and market trading responses. This perspective moves the analysis beyond single-source abnormality detection and toward a structured representation of risk across multiple information channels.

This view is especially relevant for screening-oriented regulatory settings. Manipulation strategies may differ substantially in their transaction-level mechanisms and observable manifestations, and trade-based, information-based, and combined manipulation patterns are unlikely to generate identical empirical signatures. A single indicator is therefore unlikely to capture their common risk features. By organizing disclosure, sentiment, and trading variables within a unified firm–month framework, this study treats manipulation-related risk as a probabilistic and multidimensional state rather than as the deterministic outcome of one causal channel. The relevant question is whether multiple public signals jointly form a risk profile that warrants further review.

At the conceptual level, the study frames manipulation-risk assessment as a problem of public-signal representation. This does not diminish the importance of transaction-level evidence for enforcement decisions. Instead, it clarifies the complementary role of multi-source risk scoring in the earlier stages of regulatory monitoring. The proposed framework connects the information-based logic of disclosure, sentiment diffusion, and market response with the regulatory need to prioritize attention under limited review capacity.

5.2	Methodological and Empirical Implications

Methodologically, the results show the value of combining textual representations with structured numerical indicators. The comparison among Logit, CatBoost, BERT + MIL, and the final BERT + MIL + MLP model indicates that different information representations capture different aspects of manipulation-related risk. Structured-feature models provide interpretable signals from disclosure characteristics, public sentiment, and market trading variables, whereas the text-only BERT + MIL model captures semantic information embedded in corporate announcements. The stronger performance of the fusion model suggests that announcement semantics and structured indicators are complementary rather than redundant for firm–month risk ranking.

The paired significance tests further refine this interpretation. The fusion model shows consistent improvements over the structured-feature baselines across AUC, PR-AUC, Recall@10%, and Recall@20%. Compared with the text-only model, the fusion model shows clearer gains in AUC, PR-AUC, and Recall@20% than in the most restrictive Recall@10% setting. This pattern suggests that multi-source fusion contributes more strongly to overall ranking quality and broader high-risk coverage than to incremental gains within the narrowest review capacity. This is consistent with the screening objective of the study, where model utility depends not only on identifying the very highest-risk observations, but also on improving prioritization across different review capacities.

The additional validation analyses further clarify the robustness and interpretation of the framework. The alternative sentiment measurement indicates that the main findings are not materially driven by the lexicon-based construction of sentiment variables. The CAR ablation is particularly informative because CAR is the strongest price-response variable in the structured feature set. Although removing CAR reduces model performance, the model still achieves an AUC of 0.8370 and a Recall@20% of 0.6121, indicating that the framework retains meaningful risk-ranking capacity even when the most influential abnormal-return signal is excluded. This result suggests that the model is not reducible to a simple price-momentum or abnormal-return detector. The chronological validation further shows that the multi-source framework remains informative in later sample periods, although temporal performance differences may reflect changes in market conditions, information environments, and regulatory enforcement patterns over time.

5.3	Regulatory Implications and Boundary Conditions

The results also indicate how multi-source risk scores can support risk-based financial supervision. In regulatory practice, supervisory resources are limited and cannot be allocated uniformly across all listed firms and time periods. A firm–month risk score can serve as a front-end screening tool by converting heterogeneous public signals into a ranked list of observations for further review. The screening utility analysis shows how different review thresholds correspond to different combinations of review burden, risk coverage, precision, and false discovery rates. In this sense, the model helps make the trade-off between coverage and review burden explicit, rather than replacing regulatory investigation.

The model output should be interpreted as a candidate-review signal rather than as direct evidence of manipulation. A high predicted risk score indicates that a firm–month observation exhibits a combination of disclosure, sentiment, and trading patterns similar to those observed in sanctioned manipulation periods. Such a signal can help allocate regulatory attention, but it should be assessed together with firm-specific events, industry conditions, market-wide shocks, contextual information, and expert judgment. This distinction is important because legitimate information shocks or sector-level fluctuations may also generate abnormal returns, sentiment bursts, and trading activity. Practical use of the model should therefore emphasize tiered review and human verification rather than automatic enforcement decisions.

Several boundary conditions should be recognized. First, the labels are constructed from ex post CSRC penalty documents, so the model learns patterns associated with detected and sanctioned manipulation cases rather than the full population of manipulation activities. Second, the firm–month structure is suitable for periodic screening but may smooth short-horizon manipulation episodes that occur within days or around specific events. Third, the model does not incorporate transaction-level order or trade records and therefore cannot identify specific manipulative actions or actors. These limitations do not undermine the screening-oriented objective of the study, but they define the appropriate scope of interpretation. Future research could extend the framework by incorporating higher-frequency trading records, event-level annotations, investor-structure information, and cross-market evidence to further evaluate the generalizability and operational value of multi-source manipulation-risk assessment. **(Pages 30–33, Section 5, lines 685–750)**

Response to Reviewer 3:

**Comment:**

*The authors investigate an increasingly critical issue in financial oversight: the automated risk assessment of stock market manipulation by fusing unstructured textual parameters with traditional structured trading data. Shifting the analytical objective from binary detection to a probabilistic tiered screening matrix provides strong, actionable utility for market regulators managing resource constraints. The revision is commendable; the inclusion of extensive sensitivity metrics—specifically the alternative Chinese transformer sentiment model, the feature ablation benchmarks in Table 7, and the 2015–2023 chronological out-of-time validation in Table 8—greatly increases the reliability and generalizability of the empirical findings. To optimize the manuscript for final publication, the authors should ensure that all acronyms (such as CSRC, MIL, and SHAP) are consistently defined upon their first appearance in the main text body, explicitly highlight in the text discussion how the model remains highly informative even when relying less on strong price-momentum signals (the CAR ablation), and add a brief sentence acknowledging that the temporal performance gap reflects structural evolutions in China's regulatory enforcement parameters over time.*

**Response:**

We sincerely thank the reviewer for the positive assessment of our revised manuscript and for the helpful suggestions for final improvement. In the present revision, we checked the acronym definitions throughout the manuscript, clarified the first appearance of BERT–Multiple Instance Learning (BERT–MIL), and ensured that key acronyms are defined in the main text.

We also revised the newly added Discussion section to address the reviewer’s two substantive suggestions. Specifically, Section 5.2 now highlights that the model remains informative after CAR is removed, with an AUC of 0.8370 and a Recall@20% of 0.6121, indicating that the framework is not reducible to a simple price-momentum or abnormal-return detector. We also added a sentence noting that temporal performance differences may reflect changes in market conditions, information environments, and regulatory enforcement patterns over time.

**Amendments in the Manuscript:**

In the Introduction, we revised the contribution statement to define BERT–MIL upon first appearance:

Second, it introduces a BERT–Multiple Instance Learning (BERT–MIL) architecture that models multiple announcements within the same period as a set and captures heterogeneous and unevenly distributed information signals that are often diluted in conventional semantic analysis. **(Pages 3–4, Section 1, lines 63–65)**

In the newly added Section 5.2, we highlighted the implication of the CAR ablation analysis:

The CAR ablation is particularly informative because CAR is the strongest price-response variable in the structured feature set. Although removing CAR reduces model performance, the model still achieves an AUC of 0.8370 and a Recall@20% of 0.6121, indicating that the framework retains meaningful risk-ranking capacity even when the most influential abnormal-return signal is excluded. This result suggests that the model is not reducible to a simple price-momentum or abnormal-return detector. **(Page 32, Section 5.2, lines 721–725)**

In Section 5.2, we also added a sentence to acknowledge the interpretation of temporal performance differences:

The chronological validation further shows that the multi-source framework remains informative in later sample periods, although temporal performance differences may reflect changes in market conditions, information environments, and regulatory enforcement patterns over time. **(Page 32, Section 5.2, lines 725–727)**

Sincerely,

Yuting Luo

On behalf of all authors
