# Supplementary material for: Risk assessment of stock market manipulation through the fusion of multi-source textual and trading data: Evidence from China’s A-share market
Source: PLoS One. 2026 Aug 7;21(8):e0343442. doi: 10.1371/journal.pone.0343442 (PMC13450784; doi:10.1371/journal.pone.0343442)
Supplement: Peer review history

# Peer review: Decision Letter 0

**DOI:** 10.1371/journal.pone.0343442.r001

**Authors:** Stefan Cristian Gherghina

7 Apr 2026

PONE-D-26-06350

Risk assessment of stock market manipulation through the fusion of multi-source textual and trading data: Evidence from China’s A-share market

PLOS One

Dear Dr. Luo,

Thank you for submitting your manuscript to PLOS ONE. After careful consideration, we feel that it has merit but does not fully meet PLOS ONE’s publication criteria as it currently stands. Therefore, we invite you to submit a revised version of the manuscript that addresses the points raised during the review process.

The submission requires further revisions with reference to the theoretical framework, as well as the empirical methodology.

Please submit your revised manuscript by May 22 2026 11:59PM. If you will need more time than this to complete your revisions, please reply to this message or contact the journal office at plosone@plos.org. When you're ready to submit your revision, log on to https://www.editorialmanager.com/pone/ and select the 'Submissions Needing Revision' folder to locate your manuscript file.

Please include the following items when submitting your revised manuscript:

- A letter that responds to each point raised by the academic editor and reviewer(s). You should upload this letter as a separate file labeled 'Response to Reviewers'.
- A marked-up copy of your manuscript that highlights changes made to the original version. You should upload this as a separate file labeled 'Revised Manuscript with Track Changes'.
- An unmarked version of your revised paper without tracked changes. You should upload this as a separate file labeled 'Manuscript'.

If you would like to make changes to your financial disclosure, please include your updated statement in your cover letter. Guidelines for resubmitting your figure files are available below the reviewer comments at the end of this letter.

If applicable, we recommend that you deposit your laboratory protocols in protocols.io to enhance the reproducibility of your results. Protocols.io assigns your protocol its own identifier (DOI) so that it can be cited independently in the future. For instructions see: https://journals.plos.org/plosone/s/submission-guidelines#loc-laboratory-protocols. Additionally, PLOS ONE offers an option for publishing peer-reviewed Lab Protocol articles, which describe protocols hosted on protocols.io. Read more information on sharing protocols at https://plos.org/protocols?utm_medium=editorial-email&utm_source=authorletters&utm_campaign=protocols.

As the corresponding author, your ORCID iD is verified in the submission system and will appear in the published article. PLOS supports the use of ORCID, and we encourage all coauthors to register for an ORCID iD and use it as well. Please encourage your coauthors to verify their ORCID iD within the submission system before final acceptance, as unverified ORCID iDs will not appear in the published article. *Only*  the individual author can complete the verification step; PLOS staff *cannot*  verify ORCID iDs on behalf of authors.

We look forward to receiving your revised manuscript.

Kind regards,

Stefan Cristian Gherghina, PhD. Habil.

Academic Editor

PLOS One

Journal Requirements:

When submitting your revision, we need you to address these additional requirements.

1.Please ensure that your manuscript meets PLOS ONE's style requirements, including those for file naming. The PLOS ONE style templates can be found at

https://journals.plos.org/plosone/s/file?id=wjVg/PLOSOne_formatting_sample_main_body.pdf and

https://journals.plos.org/plosone/s/file?id=ba62/PLOSOne_formatting_sample_title_authors_affiliations.pdf

2. Please note that PLOS One has specific guidelines on code sharing for submissions in which author-generated code underpins the findings in the manuscript. In these cases, we expect all author-generated code to be made available without restrictions upon publication of the work. Please review our guidelines at https://journals.plos.org/plosone/s/materials-and-software-sharing#loc-sharing-code and ensure that your code is shared in a way that follows best practice and facilitates reproducibility and reuse.

3. Thank you for stating the following financial disclosure:

“This research was funded by the Project of National Key Research and Development Program Project (2024YFC3307904).”

Please state what role the funders took in the study.  If the funders had no role, please state: "The funders had no role in study design, data collection and analysis, decision to publish, or preparation of the manuscript."

If this statement is not correct you must amend it as needed.

Please include this amended Role of Funder statement in your cover letter; we will change the online submission form on your behalf.

4. We note that you have indicated that there are restrictions to data sharing for this study. PLOS only allows data to be available upon request if there are legal or ethical restrictions on sharing data publicly. For more information on unacceptable data access restrictions, please see http://journals.plos.org/plosone/s/data-availability#loc-unacceptable-data-access-restrictions.

Before we proceed with your manuscript, please address the following prompts:

a) If there are ethical or legal restrictions on sharing a de-identified data set, please explain them in detail (e.g., data contain potentially identifying or sensitive patient information, data are owned by a third-party organization, etc.) and who has imposed them (e.g., a Research Ethics Committee or Institutional Review Board, etc.). Please also provide contact information for a data access committee, ethics committee, or other institutional body to which data requests may be sent.

b) If there are no restrictions, please upload the minimal anonymized data set necessary to replicate your study findings to a stable, public repository and provide us with the relevant URLs, DOIs, or accession numbers. For a list of recommended repositories, please see

https://journals.plos.org/plosone/s/recommended-repositories. You also have the option of uploading the data as Supporting Information files, but we would recommend depositing data directly to a data repository if possible.

We will update your Data Availability statement on your behalf to reflect the information you provide.

5. Please include captions for your Supporting Information files at the end of your manuscript, and update any in-text citations to match accordingly. Please see our Supporting Information guidelines for more information: http://journals.plos.org/plosone/s/supporting-information.

6. If the reviewer comments include a recommendation to cite specific previously published works, please review and evaluate these publications to determine whether they are relevant and should be cited. There is no requirement to cite these works unless the editor has indicated otherwise.

[Note: HTML markup is below. Please do not edit.]

Reviewers' comments:

Reviewer's Responses to Questions

**Comments to the Author**

1. Is the manuscript technically sound, and do the data support the conclusions?

The manuscript must describe a technically sound piece of scientific research with data that supports the conclusions. Experiments must have been conducted rigorously, with appropriate controls, replication, and sample sizes. The conclusions must be drawn appropriately based on the data presented.

Reviewer #1: Partly

Reviewer #2: Partly

Reviewer #3: Yes

**********

2. Has the statistical analysis been performed appropriately and rigorously?

Reviewer #1: No

Reviewer #2: I Don't Know

Reviewer #3: Yes

**********

3. Have the authors made all data underlying the findings in their manuscript fully available?

The PLOS Data policy requires authors to make all data underlying the findings described in their manuscript fully available without restriction, with rare exception (please refer to the Data Availability Statement in the manuscript PDF file). The data should be provided as part of the manuscript or its supporting information, or deposited to a public repository. For example, in addition to summary statistics, the data points behind means, medians and variance measures should be available. If there are restrictions on publicly sharing data—e.g. participant privacy or use of data from a third party—those must be specified.

Reviewer #1: No

Reviewer #2: Yes

Reviewer #3: Yes

**********

4. Is the manuscript presented in an intelligible fashion and written in standard English?

PLOS ONE does not copyedit accepted manuscripts, so the language in submitted articles must be clear, correct, and unambiguous. Any typographical or grammatical errors should be corrected at revision, so please note any specific errors here.

Reviewer #1: Yes

Reviewer #2: Yes

Reviewer #3: Yes

**********

5. Review Comments to the Author

Please use the space provided to explain your answers to the questions above. You may also include additional comments for the author, including concerns about dual publication, research ethics, or publication ethics. (Please upload your review as an attachment if it exceeds 20,000 characters)

Reviewer #1: Dear Authors,

As an analyst, I have read very carefully your paper when reviewing “Risk assessment of stock market manipulation by fusion of textual and trading data from China’s A-share market” (PONE-D-26-06350). It presents a very interesting and timely research with content/trade data for manipulation detection. The current paper is informative on an extremely salient part, but is clearly lacking theoretical foundation, operational transparency and methodological understanding as far as I can see. And I will make comments below. In particular, I also have to get a summary.

1- In the introduction although it recognizes that with the increasing complexity of stock manipulation issues you get too generic to have so many conversations about these issues. And you should definitely explain different manipulation types (e.g. trade-based vs. information-based) and to position your research in that perspective.

2 -Your claimed contribution of “multi-source data fusion” fails to distinguish from that already considered in the literature. Similar approaches have been widely examined in financial analytics and regulatory technology. It’s important that you understand on a more concrete level you need to come for new features to be brought in your framework.

3- Equation (1), used for sentiment scoring, depends on a rather superficial lexicon -based approach. Since NLP with more sophisticated frameworks (for instance transformer-based models) is now the most popular, this seems outdated. A stronger argument or better comparison with a more advanced technique must be included here.

4- Equation (2), the Fog Index, is presented as a proxy for the manipulation risk in terms of textual complexity. Nevertheless, the argument for readability related to manipulation seems not to have developed effectively. An empirical agreement with readability should be made for such a strong theoretical motivation.

5- In Table we use several variables (eg, announcement sentiment, post count) without any good theoretical basis. The evidence for each of these factors should be established within prior studies (e.g.,) or clearly justified for their relevance to manipulation detection.

6- The overall framework is shown in Figure but the interaction between modules is not well-defined. It is not clear whether textual feature and trading feature are processed separately and fused together or separately throughout the pipeline and subsequently combined in any way.

7- In Section 4.1.1 we employ CatBoost to be a baseline model. Unfortunately we do not add details of hyperparameter tuning or validation strategy. It does not lead us to conclude that model comparisons are fair from the statistical perspective.

8- Table 3 reports the model performance but no statistical significance tests are given. As no formal testing is provided (be it paired t-tests or bootstrapping procedures) these improvements are not yet obvious.

9 : Fig. 4 (radar chart) does not display nicely labeled axes and numerical scales; this drastically reduces interpretation. The figure should have value and to have more quantitative meaning.

10- The 1:5 matching ratio used in sample construction isn't justified. Explain on class imbalance and model performance reasons.

11- The use of regulatory penalties as labels can create a degree of look-ahead bias, even to the extent violations are identified ex post. This issue should be acknowledged and addressed.

12- According to Section 4.1.3, we cannot precisely characterize the architecture of the fusion model. The MLP structure (number of layers, number of neurons, activation functions) must be defined in a compact way in order for reproducibility to be ensured.

13- SHAP analysis (Figure 5) finds CAR to be the most important variable. CAR is a factor that directly receives price changes so the model is, in reality, generating price anomalies and not only manipulating them so much. We say that this could potentially be a problem.

The evaluation metric in Table 3 does not measure variability (e.g. standard deviation or confidence intervals). Without them, the stability of the results are meaningless.

15- The discussion section is short and relatively generalizing and not sufficient in detail of the literature. You need to be more critical in describing and assessing your observations with reference to other studies and to better understand the benefits and dangers in theory and regulatory practice.

16- The manuscript is lacking greater clarity, especially on practical applications. In particular: with regulation, market participants can see a role of your model in real monitoring systems? This idea is currently still unappreciated.

Reviewer #2: The subject of the study is original and valuable. However, it can be clearly stated that the study suffers from a significant lack of literature review. A research of this level should have incorporated far more sources and provided a much more in-depth discussion. In fact, due to the scarcity of the literature, the discussion section has been entirely overlooked and remains unwritten. The conclusion section is too brief, and the need for a proper 'implications' section is evident. Frankly speaking, these deficiencies are sufficient for the manuscript to receive a major revision. I believe the study must be revised so that a more thorough evaluation can be conducted in the second round.

Reviewer #3: The manuscript addresses a critical and timely challenge in financial regulation: the increasing concealment of stock market manipulation. The authors propose a supervised learning framework that shifts the paradigm from simple binary classification to a tiered risk assessment approach. By fusing structured trading data with unstructured textual data—specifically corporate announcements and investor sentiment—the study constructs a "firm-month" level panel dataset based on Chinese enforcement cases. Methodologically, the work leverages BERT for semantic feature extraction and SHAP for model interpretability, demonstrating that multi-source fusion significantly outperforms single-source baselines in metrics like AUC and Recall. This research provides both a theoretical contribution to understanding the "information disclosure-sentiment reaction-trading behavior" chain and a practical tool for regulatory tiered supervision.

Comments for the Authors.

1. Conceptual Framework and Theoretical Depth: The authors effectively argue that modern manipulation is a "multi-source coupling" phenomenon. However, the transition from the theoretical drivers discussed in Section 3.1 to the specific variables selected in Section 3.2 could be more explicitly linked. For instance, while the paper mentions "nonlinear feedback loops" in manipulation risk, the current supervised learning framework primarily treats these features as parallel inputs. A more detailed discussion on how the model specifically captures the interaction or temporal lag between an announcement (information) and the subsequent sentiment surge (reaction) would strengthen the "chain" argument.

2. Methodological Rigor and BERT Implementation

The use of BERT for extracting features from corporate announcements is a modern and appropriate choice. To improve transparency, the authors should provide more details on the fine-tuning process of the BERT model. Specifically, it is important to clarify if the BERT model was pre-trained on a general Chinese corpus or a specialized financial corpus, as the latter significantly impacts the capture of nuanced financial sentiment and "fog" (complexity) indices. Additionally, since the "announcement fog" is a key variable, the authors should detail how semantic complexity is numerically derived from the BERT embeddings to ensure replicability.

3. Data Labeling and Potential Bias

The study relies on "retrospectively labeling violation periods" based on CSRC-sanctioned cases. While this is a standard approach, it introduces a "survivorship bias" where the model only learns from manipulators who were caught. The authors should include a "Limitations" section discussing how the features of detected manipulation might differ from undetected manipulation. Furthermore, the firm-month granularity is practical for regulation but might smooth over high-frequency "pump and dump" schemes that occur within days; a justification for the monthly frequency over weekly or daily windows would be beneficial.

4. Interpretability and SHAP Analysis

The application of SHAP to provide "actionable insights" is a highlight of the paper. The authors should expand Figure 5 and the related text to discuss not just the global importance of features like "CAR" (Cumulative Abnormal Return) or "ann_cnt," but also the direction of their influence. For example, does a high "ann_fog" consistently increase or decrease the risk probability in the model’s view? Providing a case study of a specific "Risk (1)" firm and its SHAP waterfall plot would bridge the gap between abstract probability and regulatory practice.

5. Comparative Performance and Baseline Robustness

The empirical results show the fusion model outperforms single-source baselines. To make this more robust, the authors should compare their fusion approach against other state-of-the-art ensemble methods or graph-based models (like GCNs) which are increasingly used to capture "interconnected price reactions" mentioned in the introduction. This would place the BERT-fusion framework within the current frontier of RegTech research.

**********

6. PLOS authors have the option to publish the peer review history of their article (what does this mean?). If published, this will include your full peer review and any attached files.

If you choose “no”, your identity will remain anonymous but your review may still be made public.

**Do you want your identity to be public for this peer review?**  For information about this choice, including consent withdrawal, please see our Privacy Policy.

Reviewer #1: **Yes:** Seyed Pendar Toufighi

Reviewer #2: No

Reviewer #3: **Yes:** Prof.(Dr.) Santhosh Kumar P K

**********

[NOTE: If reviewer comments were submitted as an attachment file, they will be attached to this email and accessible via the submission site. Please log into your account, locate the manuscript record, and check for the action link "View Attachments". If this link does not appear, there are no attachment files.]

To ensure your figures meet our technical requirements, please review our figure guidelines: https://journals.plos.org/plosone/s/figures

You may also use PLOS’s free figure tool, NAAS, to help you prepare publication quality figures: https://journals.plos.org/plosone/s/figures#loc-tools-for-figure-preparation.

NAAS will assess whether your figures meet our technical requirements by comparing each figure against our figure specifications.

Attachment Submitted filename: 22 02 26 PlosOne REV.docx

# Author comment: Author response to Decision Letter 1

**DOI:** 10.1371/journal.pone.0343442.r002

14 May 2026

We have uploaded a detailed point-by-point response to the editor’s and reviewers’ comments in the file “Response to Reviewers.docx”.

Attachment Submitted filename: Response to Reviewers.docx

# Peer review: Decision Letter 1

**DOI:** 10.1371/journal.pone.0343442.r003

**Authors:** Stefan Cristian Gherghina

5 Jun 2026

PONE-D-26-06350R1

Risk assessment of stock market manipulation through the fusion of multi-source textual and trading data: Evidence from China’s A-share market

PLOS One

Dear Dr. Luo,

Thank you for submitting your manuscript to PLOS ONE. After careful consideration, we feel that it has merit but does not fully meet PLOS ONE’s publication criteria as it currently stands. Therefore, we invite you to submit a revised version of the manuscript that addresses the points raised during the review process.

Please submit your revised manuscript by Jul 20 2026 11:59PM. If you will need more time than this to complete your revisions, please reply to this message or contact the journal office at plosone@plos.org. When you're ready to submit your revision, log on to https://www.editorialmanager.com/pone/ and select the 'Submissions Needing Revision' folder to locate your manuscript file.

Please include the following items when submitting your revised manuscript:

- A letter that responds to each point raised by the academic editor and reviewer(s). You should upload this letter as a separate file labeled 'Response to Reviewers'.
- A marked-up copy of your manuscript that highlights changes made to the original version. You should upload this as a separate file labeled 'Revised Manuscript with Track Changes'.
- An unmarked version of your revised paper without tracked changes. You should upload this as a separate file labeled 'Manuscript'.

If you would like to make changes to your financial disclosure, please include your updated statement in your cover letter. Guidelines for resubmitting your figure files are available below the reviewer comments at the end of this letter.

If applicable, we recommend that you deposit your laboratory protocols in protocols.io to enhance the reproducibility of your results. Protocols.io assigns your protocol its own identifier (DOI) so that it can be cited independently in the future. For instructions see: https://journals.plos.org/plosone/s/submission-guidelines#loc-laboratory-protocols. Additionally, PLOS ONE offers an option for publishing peer-reviewed Lab Protocol articles, which describe protocols hosted on protocols.io. Read more information on sharing protocols at https://plos.org/protocols?utm_medium=editorial-email&utm_source=authorletters&utm_campaign=protocols.

As the corresponding author, your ORCID iD is verified in the submission system and will appear in the published article. PLOS supports the use of ORCID, and we encourage all coauthors to register for an ORCID iD and use it as well. Please encourage your coauthors to verify their ORCID iD within the submission system before final acceptance, as unverified ORCID iDs will not appear in the published article. *Only*  the individual author can complete the verification step; PLOS staff *cannot*  verify ORCID iDs on behalf of authors.

We look forward to receiving your revised manuscript.

Kind regards,

Stefan Cristian Gherghina, PhD. Habil.

Academic Editor

PLOS One

Journal Requirements:

If the reviewer comments include a recommendation to cite specific previously published works, please review and evaluate these publications to determine whether they are relevant and should be cited. There is no requirement to cite these works unless the editor has indicated otherwise.

**Additional Editor Comments:**

The remaing comments of the second referee should be further addressed.

[Note: HTML markup is below. Please do not edit.]

Reviewers' comments:

Reviewer's Responses to Questions

**Comments to the Author**

1. If the authors have adequately addressed your comments raised in a previous round of review and you feel that this manuscript is now acceptable for publication, you may indicate that here to bypass the “Comments to the Author” section, enter your conflict of interest statement in the “Confidential to Editor” section, and submit your "Accept" recommendation.

Reviewer #1: All comments have been addressed

Reviewer #2: All comments have been addressed

Reviewer #3: All comments have been addressed

**********

2. Is the manuscript technically sound, and do the data support the conclusions?

The manuscript must describe a technically sound piece of scientific research with data that supports the conclusions. Experiments must have been conducted rigorously, with appropriate controls, replication, and sample sizes. The conclusions must be drawn appropriately based on the data presented.

Reviewer #1: Yes

Reviewer #2: Yes

Reviewer #3: Yes

**********

3. Has the statistical analysis been performed appropriately and rigorously?

Reviewer #1: Yes

Reviewer #2: I Don't Know

Reviewer #3: Yes

**********

4. Have the authors made all data underlying the findings in their manuscript fully available?

The PLOS Data policy requires authors to make all data underlying the findings described in their manuscript fully available without restriction, with rare exception (please refer to the Data Availability Statement in the manuscript PDF file). The data should be provided as part of the manuscript or its supporting information, or deposited to a public repository. For example, in addition to summary statistics, the data points behind means, medians and variance measures should be available. If there are restrictions on publicly sharing data—e.g. participant privacy or use of data from a third party—those must be specified.

Reviewer #1: Yes

Reviewer #2: Yes

Reviewer #3: Yes

**********

5. Is the manuscript presented in an intelligible fashion and written in standard English?

PLOS ONE does not copyedit accepted manuscripts, so the language in submitted articles must be clear, correct, and unambiguous. Any typographical or grammatical errors should be corrected at revision, so please note any specific errors here.

Reviewer #1: Yes

Reviewer #2: Yes

Reviewer #3: Yes

**********

6. Review Comments to the Author

Please use the space provided to explain your answers to the questions above. You may also include additional comments for the author, including concerns about dual publication, research ethics, or publication ethics. (Please upload your review as an attachment if it exceeds 20,000 characters)

Reviewer #1: (No Response)

Reviewer #2: This study still needs a theoretical foundation and a discussion parts. It was suggested to you in my first review.

Reviewer #3: The authors investigate an increasingly critical issue in financial oversight: the automated risk assessment of stock market manipulation by fusing unstructured textual parameters with traditional structured trading data. Shifting the analytical objective from binary detection to a probabilistic tiered screening matrix provides strong, actionable utility for market regulators managing resource constraints. The revision is commendable; the inclusion of extensive sensitivity metrics—specifically the alternative Chinese transformer sentiment model, the feature ablation benchmarks in Table 7, and the 2015–2023 chronological out-of-time validation in Table 8—greatly increases the reliability and generalizability of the empirical findings. To optimize the manuscript for final publication, the authors should ensure that all acronyms (such as CSRC, MIL, and SHAP) are consistently defined upon their first appearance in the main text body, explicitly highlight in the text discussion how the model remains highly informative even when relying less on strong price-momentum signals (the CAR ablation), and add a brief sentence acknowledging that the temporal performance gap reflects structural evolutions in China's regulatory enforcement parameters over time.

**********

7. PLOS authors have the option to publish the peer review history of their article (what does this mean?). If published, this will include your full peer review and any attached files.

If you choose “no”, your identity will remain anonymous but your review may still be made public.

**Do you want your identity to be public for this peer review?**  For information about this choice, including consent withdrawal, please see our Privacy Policy.

Reviewer #1: **Yes:** Seyed Pendar Toufighi

Reviewer #2: No

Reviewer #3: No

**********

[NOTE: If reviewer comments were submitted as an attachment file, they will be attached to this email and accessible via the submission site. Please log into your account, locate the manuscript record, and check for the action link "View Attachments". If this link does not appear, there are no attachment files.]

To ensure your figures meet our technical requirements, please review our figure guidelines: https://journals.plos.org/plosone/s/figures

You may also use PLOS’s free figure tool, NAAS, to help you prepare publication quality figures: https://journals.plos.org/plosone/s/figures#loc-tools-for-figure-preparation.

NAAS will assess whether your figures meet our technical requirements by comparing each figure against our figure specifications.

# Author comment: Author response to Decision Letter 2

**DOI:** 10.1371/journal.pone.0343442.r004

15 Jun 2026

We sincerely thank the Academic Editor and reviewers for their constructive comments. We have revised the manuscript accordingly, and a detailed point-by-point response to all comments is provided in the uploaded “Response to Reviewers” file.

Attachment Submitted filename: Response_to_Reviewers_auresp_2.docx

# Peer review: Decision Letter 2

**DOI:** 10.1371/journal.pone.0343442.r005

**Authors:** Stefan Cristian Gherghina

10 Jul 2026

Risk assessment of stock market manipulation through the fusion of multi-source textual and trading data: Evidence from China’s A-share market

PONE-D-26-06350R2

Dear Dr. Luo,

We’re pleased to inform you that your manuscript has been judged scientifically suitable for publication and will be formally accepted for publication once it meets all outstanding technical requirements.

Within one week, you’ll receive an e-mail detailing the required amendments. When these have been addressed, you’ll receive a formal acceptance letter and your manuscript will be scheduled for publication.

An invoice will be generated when your article is formally accepted. Please note, if your institution has a publishing partnership with PLOS and your article meets the relevant criteria, all or part of your publication costs will be covered. Please make sure your user information is up-to-date by logging into Editorial Manager at Editorial Manager® and clicking the ‘Update My Information' link at the top of the page. For questions related to billing, please contact billing support.

If your institution or institutions have a press office, please notify them about your upcoming paper to help maximize its impact. If they’ll be preparing press materials, please inform our press team as soon as possible -- no later than 48 hours after receiving the formal acceptance. Your manuscript will remain under strict press embargo until 2 pm Eastern Time on the date of publication. For more information, please contact onepress@plos.org.

Kind regards,

Stefan Cristian Gherghina, PhD. Habil.

Academic Editor

PLOS One

Additional Editor Comments (optional): The remaining comments of the third reviewer should be implemented.

Reviewers' comments:

Reviewer's Responses to Questions

**Comments to the Author**

1. If the authors have adequately addressed your comments raised in a previous round of review and you feel that this manuscript is now acceptable for publication, you may indicate that here to bypass the “Comments to the Author” section, enter your conflict of interest statement in the “Confidential to Editor” section, and submit your "Accept" recommendation.

Reviewer #2: All comments have been addressed

Reviewer #3: All comments have been addressed

**********

2. Is the manuscript technically sound, and do the data support the conclusions?

The manuscript must describe a technically sound piece of scientific research with data that supports the conclusions. Experiments must have been conducted rigorously, with appropriate controls, replication, and sample sizes. The conclusions must be drawn appropriately based on the data presented.

Reviewer #2: Yes

Reviewer #3: Yes

**********

3. Has the statistical analysis been performed appropriately and rigorously?

Reviewer #2: Yes

Reviewer #3: Yes

**********

4. Have the authors made all data underlying the findings in their manuscript fully available?

The PLOS Data policy requires authors to make all data underlying the findings described in their manuscript fully available without restriction, with rare exception (please refer to the Data Availability Statement in the manuscript PDF file). The data should be provided as part of the manuscript or its supporting information, or deposited to a public repository. For example, in addition to summary statistics, the data points behind means, medians and variance measures should be available. If there are restrictions on publicly sharing data—e.g. participant privacy or use of data from a third party—those must be specified.

Reviewer #2: No

Reviewer #3: No

**********

5. Is the manuscript presented in an intelligible fashion and written in standard English?

PLOS ONE does not copyedit accepted manuscripts, so the language in submitted articles must be clear, correct, and unambiguous. Any typographical or grammatical errors should be corrected at revision, so please note any specific errors here.

Reviewer #2: Yes

Reviewer #3: Yes

**********

6. Review Comments to the Author

Please use the space provided to explain your answers to the questions above. You may also include additional comments for the author, including concerns about dual publication, research ethics, or publication ethics. (Please upload your review as an attachment if it exceeds 20,000 characters)

Reviewer #2: The authors have made all corrections according to my suggestions. Theoretical framework and discussion parts are now sound.

Reviewer #3: Reviewer Comments

Thank you for the opportunity to review the revised manuscript. The authors have effectively addressed the previous concerns, particularly by providing a robust theoretical foundation and a comprehensive discussion section. The addition of chronological validation and feature ablation analysis significantly strengthens the empirical contributions of this study.

I have a few minor remaining suggestions to finalize the manuscript:

1. Clarity on "Regulatory Enforcement Patterns"

In the Discussion section (Section 5.2), you mention that temporal performance differences may reflect "changes in market conditions, information environments, and regulatory enforcement patterns over time." To make this more specific for the reader, please briefly clarify what kind of changes in enforcement patterns are implied (e.g., shifts in CSRC focus, changes in enforcement intensity, or changes in how cases are disclosed).

2. Terminology and Formatting

Consistency: Please conduct a final thorough check to ensure all acronyms are defined upon their first mention in the main text. While most are defined, ensuring consistency across all sections—including table captions and figures—will improve readability.

Table 7: Ensure the formatting for the "Full feature set" row is consistent and error-free, specifically addressing the formatting issue in the AUC column Equation 1: Please verify the formatting for Equation (1). The denominator currently appears as Pos i+Neg_i+1 which is correct, but ensure that the formatting remains consistent with standard LaTeX rendering throughout the document.

3. Data Availability Statement

You have correctly noted that original announcement texts cannot be shared due to licensing restrictions. Please ensure the instructions for researchers to access the data via the Choice database are as clear as possible. If there is a specific contact email or a direct link for a "Choice Data Access FAQ" that could be added to the Data Availability Statement, it would be highly beneficial for future reproducibility.

Overall, the manuscript is well-structured, methodologically sound, and provides clear practical value for financial regulatory technology. I recommend that it be accepted for publication after these minor technical corrections are addressed.

**********

7. PLOS authors have the option to publish the peer review history of their article (what does this mean?). If published, this will include your full peer review and any attached files.

If you choose “no”, your identity will remain anonymous but your review may still be made public.

**Do you want your identity to be public for this peer review?**  For information about this choice, including consent withdrawal, please see our Privacy Policy.

Reviewer #2: No

Reviewer #3: **Yes:** Prof.(Dr.). Santhosh Kumar P K

**********

# Editor report: Acceptance letter

**DOI:** 10.1371/journal.pone.0343442.r006

**Authors:** Stefan Cristian Gherghina

PONE-D-26-06350R2

PLOS One

Dear Dr. Luo,

I'm pleased to inform you that your manuscript has been deemed suitable for publication in PLOS One. Congratulations! Your manuscript is now being handed over to our production team.

At this stage, our production department will prepare your paper for publication. This includes ensuring the following:

* All references, tables, and figures are properly cited

* All relevant supporting information is included in the manuscript submission,

* There are no issues that prevent the paper from being properly typeset

You will receive further instructions from the production team, including instructions on how to review your proof when it is ready. Please keep in mind that we are working through a large volume of accepted articles, so please give us a few days to review your paper and let you know the next and final steps.

Lastly, if your institution or institutions have a press office, please let them know about your upcoming paper now to help maximize its impact. If they'll be preparing press materials, please inform our press team within the next 48 hours. Your manuscript will remain under strict press embargo until 2 pm Eastern Time on the date of publication. For more information, please contact onepress@plos.org.

You will receive an invoice from PLOS for your publication fee after your manuscript has reached the completed accept phase. If you receive an email requesting payment before acceptance or for any other service, this may be a phishing scheme. Learn how to identify phishing emails and protect your accounts at https://explore.plos.org/phishing.

If we can help with anything else, please email us at customercare@plos.org.

Thank you for submitting your work to PLOS ONE and supporting open access.

Kind regards,

PLOS ONE Editorial Office Staff

on behalf of

Dr. Stefan Cristian Gherghina

Academic Editor

PLOS One
